# Promoter engineering for microbial bio-alkane gas production

**DOI:** 10.1093/synbio/ysaa022

**Published:** 2020-10-27

**Authors:** Duangthip Trisrivirat, John M X Hughes, Robin Hoeven, Matthew Faulkner, Helen Toogood, Pimchai Chaiyen, Nigel S Scrutton

**Affiliations:** y1 Department of Chemistry, School of Natural Sciences, EPSRC/BBSRC Future Biomanufacturing Research Hub, BBSRC/EPSRC Synthetic Biology Research Centre SYNBIOCHEM Manchester Institute of Biotechnology, The University of Manchester, Manchester M1 7DN, UK; y2 School of Biomolecular Science and Engineering, Vidyasirimedhi Inistitute of Science and Technology (VISTEC), Rayong 21210, Thailand; y3 Department of Biochemistry, Faculty of Science, Mahidol University, Bangkok 10400, Thailand

**Keywords:** constitutive promoter engineering, bio-propane, *Halomonas*, *Escherichia coli*

## Abstract

Successful industrial biotechnological solutions to biofuels and other chemicals production rely on effective competition with existing lower-cost natural sources and synthetic chemistry approaches enabled by adopting low-cost bioreactors and processes. This is achievable by mobilizing *Halomonas* as a next generation industrial chassis, which can be cultivated under non-sterile conditions. To increase the cost effectiveness of an existing sustainable low carbon bio-propane production strategy, we designed and screened a constitutive promoter library based on the known strong porin promoter from *Halomonas*. Comparative studies were performed between *Escherichia coli* and *Halomonas* using the reporter gene red fluorescent protein (RFP). Later studies with a fatty acid photodecarboxylase-RFP fusion protein demonstrated tuneable propane production in *Halomonas* and *E. coli*, with an ∼8-fold improvement in yield over comparable isopropyl-β-D-thiogalactoside-inducible systems. This novel set of promoters is a useful addition to the synthetic biology toolbox for future engineering of *Halomonas* to make chemicals and fuels.

## 1. Introduction

Industrial biotechnology seeks to answer the increasing demands for sustainable and renewable fine chemicals, materials and biofuels production. The ultimate aim is to reduce the dependence on diminishing reserves of fossil fuels and decrease the overall carbon footprint from production to utilization (1). However, successful implementation of scaled biotechnology solutions requires cost-effective process and capital investment strategies to compete with existing natural sources and synthetic chemistry technologies (2). Major hurdles to successful bioprocess commercialization are the high energy consumption and other associated capital and running expenses. For example, high costs arise from the need for equipment and medium sterilization, stainless steel fermentation equipment, control systems for culture maintenance and sterility, downstream processing for target chemical purification and the consumption of fresh water (3, 4).

Major cost savings in scaled bioprocesses can be achieved by implementing contamination free continuous fermentations under non-sterile conditions (4, 5). This is only possible by utilizing a microbial ‘chassis’ that grows under conditions incompatible for growth of competing organisms. Halophilic and alkaliphilic microorganisms, such as *Halomonas* species, are suited for this purpose as they grow optimally at high pH and salt concentrations in waste water or seawater under non-sterile conditions (4, 6). This allows continuous cultures to be maintained in low-cost bioreactors (e.g. plastic or cement) fed on waste biomass, with little to no requirement for fresh water. Successful utilization of halophiles as microbial chassis has been demonstrated in the production of compounds such as polyhydroxyalkanoates (6), ectoine (7), hydrolytic enzymes (8, 9), biosurfactants (10) and more recently bio-propane and butane (11). Therefore, utilizing *Halomonas* as a host is a potential game-changer, leading us into the next generation of industrial biotechnology of cost-effective bio-platforms for chemicals production (3–5, 12). 

Recent interest in *Halomonas* bio-propane production is fuelled by acknowledgement that a transition towards a clean-burning renewable and sustainable fuel would contribute towards achieving current global greenhouse gas emissions reduction targets and reducing the overall carbon footprint compared to fossil fuels (3, 11, 13–17). This is in line with current predictions that gaseous biofuels will make up a significant proportion of transport and energy generation fuels by 2030, with the current market reaching 20 million tonnes propane per annum (18, 19). In the simplest case, bioengineered alkane gas production in *Halomonas* can be achieved by the incorporation of a fatty acid photodecarboxylase variant from *Chlorella variabilis* (CvFAP_G462I_), which catalyzes the decarboxylation of butyric acid to propane (3, 11, 20, 21). Technoeconomic analysis of proposed scaled bio-propane ‘hubs’ based on this technology suggested it could become commercially competitive if further cost-cutting strategies were employed (3). This included eliminating the need for expensive and toxic additives, such as chemical inducers of recombinant protein expression (isopropyl-β-D-thiogalactoside or IPTG) and antibiotics for plasmid-borne pathway maintenance. Both were achieved by the genomic integration of CvFAP_G462I_, which was placed under the control of a constitutive promoter, which successfully led to propane production in the absence of any induction or selection agents (3).

Further application of this approach for other pathways to bio-alkane gases, or the production of other biochemicals, would be enhanced by the availability of extensive libraries of *Halomonas* constitutive promoters to enable titratable protein expression. This was first demonstrated by the engineering of the variable promoter region of the *Halomonas* endogenous constitutive *P*_porin_ promoter (22). A constitutive promoter library was obtained with a 310-fold variation in transcriptional activity, which was tested with the biosynthetic pathway to poly-3-hydroxybutyrate (PHB) in *Halomonas* TD01. However, the efficacy of promoter strength on protein expression is dependent on factors such as enzyme and ribosomal binding site (RBS) DNA sequences (23, 24). Therefore, the relative promoter strength can vary from one gene (or pathway) to another, or between different microbial genera/species. We investigated this phenomenon by generating libraries of variant *P*_porin_ constitutive promoters in *Escherichia coli* and *Halomonas*, and compared the relative expression of the reporter gene red fluorescent protein (RFP) and biocatalytic CvFAP, within plasmid systems specific for each organism. This will ultimately lead to determining the ideal constitutive promoter system suitable for bio-LPG production, and allow the application within other biotechnological solutions in *Halomonas*.

## 2. Materials and methods

### 2.1 Materials and equipment

All chemicals, solvents and reagents were purchased from commercial suppliers and were of analytical grade or better. Propane gas standard (99.95%) was obtained from Sigma Aldrich. Media components were obtained from Formedium (Norfolk, UK). Polymerase chain reaction (PCR) amplification reactions were performed using the CloneAmp premix (Takara, Japan), while In-Fusion cloning was used for plasmid re-circularization (Takara, Japan). The *E. coli* strain used for propagating plasmids and *in vivo* production was NEB5α (New England Biolabs, USA). A modified *Halomonas* TD01 strain (TQ10) was used as described previously (3). Gene sequencing and oligonucleotide synthesis were performed by Eurofins MWG (Ebersberg, Germany). Details of all the sequence-verified plasmids used in this study can be found in Supplementary Table S1, and the sequences of the oligonucleotides used in cloning and mutagenesis in Supplementary Table S2. The BglBrick series of vectors were obtained from Addgene (25). The DNA sequences and accession numbers of the constructs/plasmids used for data collection are found in the Supplementary data.

### 2.2 Assembly of a *P*_porin_-like constitutive expression construct for RFP

An *E. coli* and *Halomonas*-compatible vector expressing CvFAP_G462V_ (pHal2-CvFAP_G462V_) with a *Halomonas* only IPTG-inducible promoter was constructed as described previously (3). A minimal 40-bp truncated *P*_porin_-like (22) constitutive promoter variant with its own Shine Dalgarno sequence (see Supplementary Table S3) was incorporated into pHal2-CvFAP_G462V_ between the MmP1 T7-like promoter (26) and the start codon of CvFAP_G462V_. This was performed by overlap extension PCR (27) employing two pairs of overlapping primers (see Supplementary Table S2), followed by In-Fusion cloning (28) for plasmid re-circularization, according to the manufacturers’ protocols. Attempts to clone either the native minimal 40-bp truncated *P*_porin_ promoter or its P85 variant (22) upstream of CvFAP_G462V_ were unsuccessful, as only mutated forms were obtained. Instead, an initial round of random mutagenesis was performed on the variable 14 bp region between the −35 and −10 boxes to generate a stable initial constitutive promoter (P7) for screening purposes (see library construction method below).

This construct (pHalT7P7-CvFAP_G462V_) was modified further in three stages, beginning with the replacement of CvFAP_G462V_ with RFP from the BioBrick vector pBbE1c-RFP (25) to generate pHalT7P7-RFP. This was followed by the elimination of the now obsolete T7-like promoter (pHal7-RFP-STag) and subsequent C-terminal S-Tag removal to generate a constitutive expression construct for RFP (pHal7-RFP). A control IPTG-inducible RFP-expressing plasmid (pHal2-RFP) was assembled by PCR linearization of pHal2-CvFAP_G462V_ (elimination of CvFAP_G462V_) and ligation to RFP from pBbE1c-RFP. A second control plasmid pHal7 was constructed by the elimination of the RFP gene from pHal7-RFP by PCR. Additional controls were two BioBrick vectors composed of RFP downstream of IPTG-inducible *pTrc* (pBbA1a) or *placUV5* (pBbA5a) promoters (25). In each case, plasmid linearization and gene amplification steps were performed by PCR followed by In-Fusion cloning to ligate the constructs. Clones were introduced into *E. coli* strain NEB5α and cultivated in Luria broth (LB; 10 g/l tryptone, 5 g/l yeast extract and 5 g/l NaCl) containing 50 µg/ml kanamycin overnight at 37°C. Each construct was confirmed by DNA sequencing.

### 2.3 Construction of a *P*_porin_-like library of promoters expressing RFP

Random mutagenesis was performed using the Q5^®^ site-directed mutagenesis kit (New England Biolabs, USA) on the variable 14 bp region (13 bp in P7) between the −35 and −10 boxes of the *P*_porin_ promoter in pHal7-RFP. PCR primers were fully randomized within the 14 bp variable region, generating a library of RFP-expressing clones (pHalV-RFP). Following In-fusion cloning, the library was transformed into *E. coli* strain NEB5α and cultivated as above. Selected library clones underwent gene sequencing in the promoter variable region (2–111 bp) to identify the sequence variations. DNA sequences of the variant *P*_porin_-like promoters can be found in Supplementary Table S3.

### 2.4 Construction of a *P*_porin_-like library expressing CvFAP_G462V_RFP fusion protein

PCR linearization of pHalT7P7-CvFAP_G462V_ was performed between the end of the gene and the terminator region, maintaining the stop codon. An RFP gene was amplified from pHal7-RFP, and ligated to the vector to generate the dual construct pHal7-FAP_G462V_RFP. To generate the fusion protein (pHal7-FAP_G462V_RFP), the stop codon of CvFAP_G462V_ was eliminated and a linker sequence (GGTTCTGCGGGTTCTGCGGCCGGTTCTGGCGAATTT) was inserted by PCR. Finally, a library of constitutively expressed pHalV-FAP_G462V_RFP fusion clones was assembled by the linearization of pHalV-RFP, eliminating the RFP gene and ligation to a FAP_G462V_RFP PCR product (from pHal7-FAP_G462V_RFP).

### 2.5 Promoter library screening in *E. coli* and *Halomonas* TQ10 by RFP fluorescence

Three independent rounds of pHalV-RFP library screening were performed in *E. coli* strain NEB5α, with each of the randomly selected 129 colonies tested in triplicate. A limited selection of 22 variant *P*_porin_ promoter clones active in *E. coli* was introduced into *Halomonas* strain TQ10 by the conjugation method described previously (3) and screened in triplicate. Cultures (1 ml) were grown in the appropriate media (LB for *E. coli* or YTN6 for *Halomonas*: 5 g/l yeast extract, 10 g/l tryptone, 60 g/l NaCl, pH 9) containing 50 µg/ml of either kanamycin (*E. coli*) or spectinomycin (*Halomonas*) in 2 ml Axygen^®^ 96-deep well plates, sealed with sterile gas permeable adhesive seals. The plates were incubated overnight at 30°C with 850 rpm agitation. Control wells contained either LB media only or cultures of the empty vector pHal7 or the IPTG-inducible clones pBbA1a-RFP, pBbA5a-RFP or pHal2-RFP in *E. coli*. Replicate cultures (200 µl; starting OD_600 nm_ ∼0.1) were set up in the same medium with the required antibiotic in 96-well microtiter plates sealed with a moisture barrier seal. Cultures were incubated at 30°C with 300 rpm agitation in a CLARIOstar^®^ Plus Plate Reader. IPTG (0.1 mM) was added to the inducible cultures once OD_600 nm_ reached 0.55, and the incubation was continued overnight as before. Both the culture optical density and relative RFP fluorescence intensity (RFI) were monitored every 5 min, the latter with excitation and emission wavelengths of 584 and 607 nm, respectively. Results are expressed as the mean of the relative fluorescence units (RFU), which is fluorescence intensity per OD_600 nm_, with error bars representing one standard deviation of the data. Statistically relevant screening data are defined as having one standard deviation of less than 30% the average RFU. Numerical data can be found in Supplementary Table S4, including that of clones excluded from the screen for having a standard deviation of more than 30% the average RFU.

### 2.6 Promoter library screening of CvFAP_G462V_RFP fusion protein in *E. coli* and *Halomonas* TQ10 by propane production and fluorescence

The production and activity of CvFAP_G462V_RFP fusion protein were screened with 10 different *P*_porin_-like promoters in both *E. coli* NEB5α and *Halomonas*. Colonies of each construct were used to inoculate LB or YTN6 medium (5 ml) containing 50 µg/ml kanamycin or spectinomycin for *E. coli* and *Halomonas*, respectively. The cultures were incubated overnight at 37°C with 190 rpm agitation. Four aliquots of each culture (1 ml) were dispensed into 4 ml glass screw cap vials with rubber seals containing 10 mM butyric acid (pH adjusted to 6.8). For inducible control clones, culture aliquots were dispensed into the same glass vials containing IPTG (0.1 mM), and incubated at 30°C with 190 rpm agitation for 2 h prior to the addition of butyric acid. Each culture was subsequently incubated overnight at 30°C with 190 rpm agitation under a blue light panel (11). At the end of the incubation, manual headspace sampling was performed for propane concentration determination, followed by measurement of culture growth (OD_600 nm_) and RFP fluorescence (FI). Results are expressed as RFU and propane production (mg propane/g cells wet weight), with error bars representing one standard deviation of the data. Cell mass (wet weight) was calculated using the conversion factor of 1.7 g/l and 2.4 g/l wet weight per OD_600 nm_ of 1.0 for *E. coli* and *Halomonas* TQ10, respectively.

### 2.7 Fermentation of *Halomonas* expressing constitutive and inducible CvFAP_G462V_

A culture of *Halomonas* TQ10 expressing the fusion protein CvFAP_G462V_RFP was cultivated in a thermostatic flat panel photobioreactor (PBR) FMT 150 (Photon Systems Instruments, Czech Republic). This contained integral culture monitoring (OD 680 nm), pH and feeding control and an LED blue light panel (465 nm; maximum photosynthetic photon flux density or PPFD = 1648 µE photons). The PBR was set up in batch mode with high salt glycerol medium at pH 6.8 (5 g/l yeast extract, 1 g/l glycerol, 60 g/l NaCl, 50 µg/ml spectinomycin and 0.5 ml/l antifoam; 400 ml), pre-equilibrated at 30°C with 60–100% stirring. An overnight starter culture (10 ml) of *Halomonas* TQ10 expressing FAP_G462V_RFP, controlled by the T7-like inducible or constitutive p102 or p69 promoters, was added and the culture was maintained at 30°C with an airflow rate of 1.21 l/min. Culture maintenance was performed with automated pH adjustment (sodium acetate), culture optical density monitoring and ambient room lighting until mid-log phase (OD_680_ ∼ 0.55). Butyric acid was added (60 mM; adjusted to pH 7.0) and the culture was illuminated with blue light (1625 µE), and maintained for ∼48–72 h. For inducible cultures, IPTG (0.2 mM) was added immediately prior to butyric acid addition. Propane production was monitored at 20 min intervals by automated headspace sampling using a Micro GC. Fermenter runs with the p59 promoter were performed as above, except the air flow was stopped for 45 minutes prior to manual headspace sampling to allow the propane levels to accumulate. Propane concentration was determined by manual headspace injection into a Micro GC. The concentrations of butyric acid, glycerol and acetate were monitored by HPLC. 

### 2.8 Analytical techniques

Propane levels from the headspace of microbial cultures in 4 ml vials were determined by manual headspace injection using an Agilent 490 Micro GC, containing an Al_2_O_3_/KCl column and a thermal conductivity detector. Headspace samples were manually introduced by syringe (0.5–2.0 ml) through a heated injector (110°C), with an injection time of 100 ms, and helium as the carrier gas (10.2 psi). During the continuous monitoring mode, fermenter exhaust gases were passed through a cooling condenser (0°C; water vapor removal) prior to flowing through the Micro GC cell, with periodic sampling (15–20 min intervals). Compounds were separated isothermally at 100°C for 120 s under static pressure conditions, with a sampling frequency of 100 Hz. Propane concentrations were calculated by comparing the peak areas to a standard curve generated using the same analytical conditions (3). The concentration of aqueous carbon sources and other metabolites were determined by HPLC using an Agilent 1260 Infinity HPLC with a 1260 refractive index detector and an Agilent Hi-Plex H column (300 × 7.7 mm; 5 mM H_2_SO_4_) as described previously (3). Analyte concentrations were calculated by comparing the peak areas to a standard curve generated under the same analytical method.

## 3. Results and discussion

### 3.1 *P*_porin_-like constitutive library screening in *E. coli*

Prior proteomic analysis of *Halomonas* TD01 identified that a major protein expressed was an outer membrane porin protein (22), which functions as a channel for the passive diffusion of nutrients (29). This gene was expressed by a strong constitutive promoter *P*_porin_, and the key elements (the −35, −10 and extended −10 sequences) and minimal 40-bp sequence were determined (22). This promoter has been reported to be one of the strongest promoters in *Halomonas bluephagenesis* when used for chromosomal gene insertions (30), and has previously been manipulated for improved production of polyhydroxyalkanoates (31). Randomization of the 14 bp between the −35 and −10 regions led to the development of a tuneable constitutive promoter library for the production of PHB (22).

We performed similar randomization of the variable region of the *P*_porin_ promoter to see if tuneable libraries could be used as a general gram-negative bacterial constitutive expression system for non-native gene (or pathway) incorporation. This is because the correlation of promoter strengths between different bacterial species is poorly understood and difficult to predict. We selected *E. coli* NEB5α as a general microorganism for promoter library construction and screening. The *Halomonas* strain chosen for comparison was TQ10, as it has undergone genomic alterations to eliminate the pathway for PHB production (3, 11) and to incorporate the T7-like system MmP1 for control IPTG-inducible recombinant protein expression (26). Initial screening was performed using RFP as the reporter gene within a pSEVA-based *Halomonas*- and *E. coli*-compatible plasmid (pHal2 (3, 26)).

The construct assembled for randomization (pHal7-RFP) contained a variant of the minimal *P*_porin_ promoter (40 bp), where the native variable 14 bp region (TCACTGGAATCCCA (22)) was substituted for an alternative 13 bp sequence (ACAACCGATAAAG). Wild-type minimal *P*_porin_ promoter was not used as the initial construct as repeated attempts to generate it in *E. coli* led to only variant forms being produced. However, this alternative 1 bp truncated construct displayed significant RFP fluorescence within *E. coli*. In addition, it bears some resemblance to variant P5 of the original study (ACACAACCGAATAT), which displayed a 2.1-fold increase in RFI compared to wild type (22).

**Figure 1.**
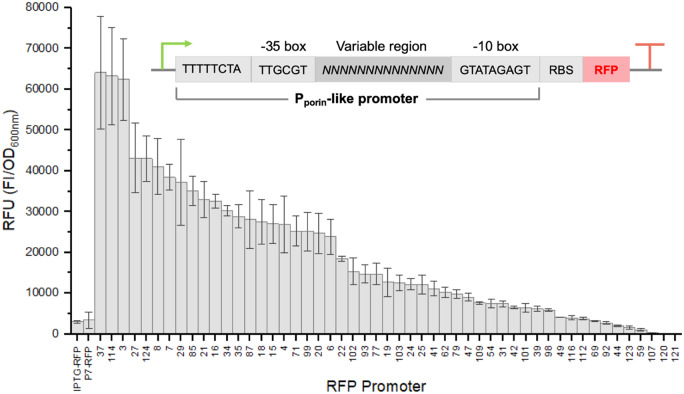
Screening of a library of *P*_porin_-like promoters expressing RFP in *E. coli* strain NEB5α. Cultures (200 µl) in LB medium containing 50 µg/ml kanamycin were incubated in 96-well microtiter plates overnight at 30°C with 300 rpm agitation in a microtiter plate reader. IPTG (0.1 mM) was added to the inducible cultures once OD_600 nm_ reached 0.55. Culture OD_600 nm_ and relative RFI were monitored every 5 min (excitation and emission wavelengths of 584 and 607 nm, respectively). Results are expressed as the mean of the RFU (RFI/OD_600 nm_) with error bars of one standard deviation. Data for additional constructs displaying errors >30% of the mean RFU are shown in Supplementary Table S4. Inset: Schematic of the library of RFP expressing clones with variable promoters (pHalV-RFP). IPTG-RFP: IPTG-inducible *P_Trc_* pBbA1a-RFP (uninduced); P7-RFP: pHal7-RFP control containing the constitutive promoter with variable region 7 from the paper by Li *et al.* (22).

Randomization of the variable region of the promoter was performed by PCR, and a library of >100 individual clones were screened in *E. coli* using a microtiter plate-based culture growth and RFP fluorescence monitoring. Individually sequenced clones were ranked according to their RFU, which is a measure of culture fluorescence intensity per unit cell density (see Figure 1 and Supplementary Table S3). Data were compared to those generated by IPTG-inducible RFP constructs, which differed by the promoter system utilized (T7-like MmP1 (26), *pTrc* or *placUV5*) and plasmid backbone (pHal2 (3) versus BglBrick vectors (25); see Supplementary Table S4).

Analysis of a subset of 51 variants with unique RFP promoter sequences showed over a 600-fold difference in RFU, with the original promoter displaying only 4.7% RFU compared to the highest variant (see Figure 1). Only 85% of the promoters contained 13 or 14 bp in the variable region, with the sequence length varying from 2 to 111 bp. Surprisingly, two clones with promoters >100 bp showed very high RFU (clones 17 and 114), while a third showed only 9% of the maximum. A control IPTG-inducible (*pTrc*) construct showed only 22% RFU compared to the best variant, but was over 4-fold higher than the original constitutive pHal7-RFP construct (see Supplementary Table S4). Overall, this screen has identified more than 50 new constitutive promoters that function in *E. coli*, with 50% displaying expression strengths higher than a strong IPTG-inducible system.

### 3.2 *P*_porin_-like sub-library screening

**Figure 2.**
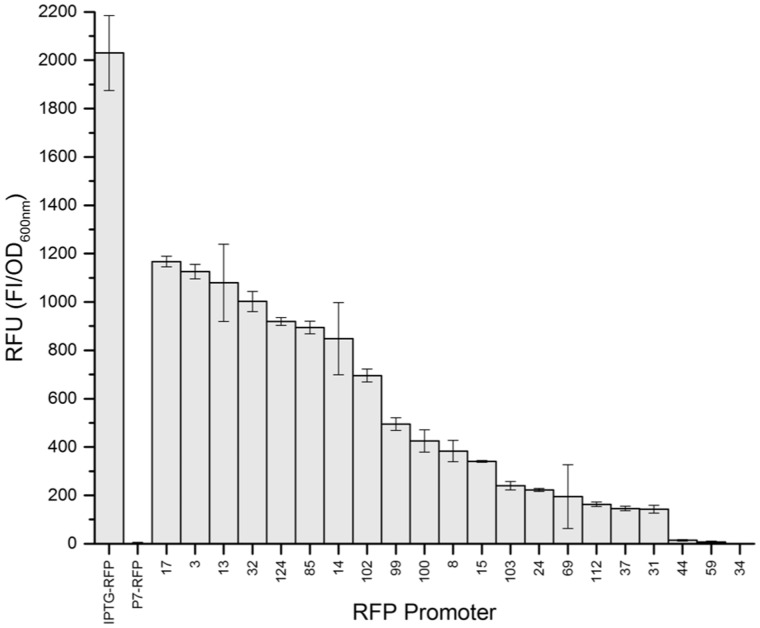
Screening of a limited set of *P*_porin_-like promoters expressing RFP in *Halomonas* TQ10. Culture growth and RFP monitoring were performed as for *E. coli*, as described in the Figure 1 legend, except the growth medium was YTN6 medium (LB with 60 g/l NaCl) with 50 µg/ml spectinomycin. Results are expressed as the mean of the RFU (RFI/OD_600 nm_) with error bars of one standard deviation. Numerical data for this figure can be found in Supplementary Table S5.

Transferring the entire *P*_porin_-RFP library into *Halomonas* was not deemed practical, as plasmid incorporation requires conjugation (26), compared to the more rapid transformation protocols available for *E. coli*. Therefore, a sub-library of 21 *P*_porin_-RFP constructs was selected for screening in *Halomonas*, which covers the entire *E. coli* expression range (see Figure 2). As a control, this sub-library was also rescreened in *E. coli* under near identical conditions as in *Halomonas*, except for the required differences in the growth medium.

There was a dramatic reduction in the overall RFP fluorescence detected in *Halomonas* cultures compared to *E. coli* (see Figure 2). For example, the best *P*_porin_-RFP construct in *E. coli* showed a 600-fold reduction of RFU in *Halomonas*. This was an unexpected finding, as the earlier study showed the fluorophore signal (in this case green fluorescent protein) correlated well between *E. coli* and *Halomonas* (31). This disparity was compounded by a lack of correlation between the relative promoter strengths between the two organisms, with an R-squared (*R*^2^) value of only 0.46. When comparing the highest performing promoters from each organism, the RFU in *Halomonas* (clone 17) was around 80-fold lower than in *E. coli* (clone 2; see Supplementary Tables S5 and S6). It is unclear whether this is a protein expression issue or if additional factors are influencing the observed RFU. For example, RFP misfolding/instability under halophilic conditions may be a contributory factor, as could potentially differences in the plasmid copy number and RBS strengths between *E. coli* and *Halomonas*.

Further differences were seen when comparing the *Halomonas* constitutive library to RFP expression controlled by the T7-like MmP1 IPTG-inducible promoter (26). In this case, the IPTG-inducible construct had RFU values 1.7-fold higher than the best constitutive promoter (see Figure 2). This suggests the limited library of promoters is not optimized for RFP expression in *Halomonas* compared to *E. coli*, in spite of the original promoter originating in *Halomonas*. This could also contribute to the comparatively lower RFU values observed in *Halomonas*.

### 3.3 Constitutive library screening for propane production

**Figure 3.**
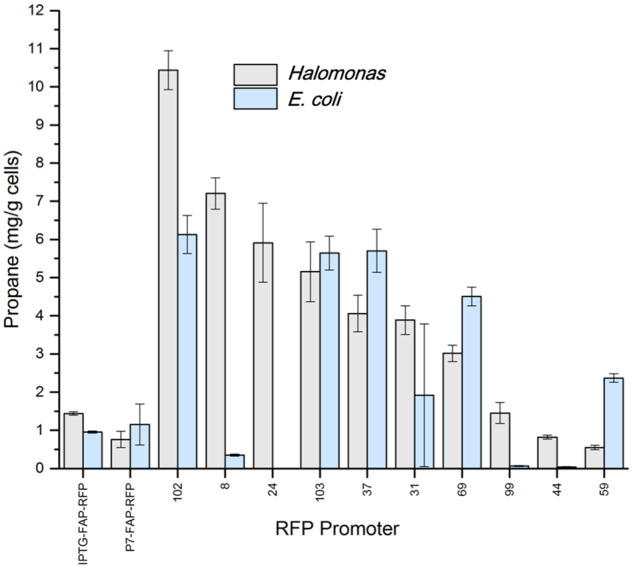
Expression of CvFAP_G462V_RFP controlled by 10 *P*_porin_-like promoters in *E. coli* NEB5α and *Halomonas* TQ10. Culture growth and RFP monitoring was performed as for *E. coli* and *Halomonas* as described in the Figures 1 and 2 legends, respectively. Propane concentration was determined by manual injection into a Micro GC. Results are expressed as propane production (mg propane/g cells wet weight), with error bars representing one standard deviation of the data. Numerical values of these data can be found in Supplementary Tables S7 and S8.

The ultimate aim of this tuneable promoter library is to utilize it within industrial microorganisms to express biocatalytic genes for the production of fine chemicals and fuels. Therefore, it is important to establish whether the relative promoter strengths are organism type and gene sequence specific. To investigate this, we screened the promoter library in *E. coli* and *Halomonas* for the expression of the biocatalyst CvFAP from *C. variabilis*. This enzyme catalyzes the blue light-dependent decarboxylation of volatile fatty acids to hydrocarbon gases (e.g. propane, butane and isobutane) (3, 11, 20, 21). For comparative purposes, we generated a CvFAP_G462V_RFP fusion protein to enable us to monitor both the RFU and propane titers. The promoter library was reduced to 10 variants, and the performance in both *E. coli* and *Halomonas* was determined (see Figure 3). To determine propane production, additional cultures of each variant were cultivated in sealed vials in the presence of butyric acid and blue light. Propane production was determined by manual headspace analysis by Micro GC.

We expected a near 1:1 correlation between the RFU and propane titers, as both activities are expressed as a single fusion protein. This was seen in the promoter screen with *Halomonas* (*R*^2^ = 0.991 RFP: propane; see Supplementary Table S7), but surprisingly not in *E. coli* (*R*^2^ = 0.367; see Supplementary Table S8). Lower than expected correlations could occur when one or both of the ‘activities’ monitored are sub-optimal or absent. For example, photoinactivation or lack of flavin incorporation of CvFAP leads to inactive protein (32, 33), while the C-terminal RFP domain may be fully active. Conversely, the RFP maturation rate may not keep pace with high protein expression rates in *E. coli* (34). The higher relative correlation seen in *Halomonas* may be in part due to the presence of high levels of the compatible solute ectoine, which in some cases can increase protein expression, stabilize and assist in the folding of proteins *in vivo* (4).

We observed a good correlation in promoter strength between the two microorganisms in many cases, when comparing propane titers alone (see Figure 3). In almost all cases, the propane levels in both microorganisms were comparable, with titers between 2- and 10-fold higher than an IPTG-inducible construct. This differs from comparative RFP expression between the two microorganisms, where RFP fluorescence was considerably higher in *E. coli* (Figures 1 and 2). There was a dynamic range in propane titers of around 20 for the limited promoter screen in *Halomonas* (0.55 ± 0.06 to 10.44 ± 0.51 mg propane/g cells from promoter 59 to 102, respectively). The best-performing promoters had different sequences to those producing the highest RFU when RFP was expressed alone. Therefore, the relative performance of this promoter library is both microorganism and protein sequence specific.

### 3.4 Fermentative propane production in *Halomonas*

To determine the ideal constitutive promoter for *in vivo* biocatalysis, maximizing the promoter strength must be balanced against the reduction in host fitness associated with recombinant protein overexpression. To investigate this, we determined the growth profiles of *Halomonas* TQ10 expressing CvFAP_G462V_RFP fusion under control of high, medium and low strength constitutive promoters (p102, p69 and p59, respectively) and the MmP1 inducible promoter. As expected, *Halomonas* growth declined significantly when utilizing the highest strength promoter, while no significant differences were seen in the presence of the medium and low strength promoter (see Supplementary Figure S1). The inducible strain showed a typical decline in growth rate after IPTG induction.

We performed small-scale fermentations of *Halomonas* TQ10 expressing CvFAP_G462V_RFP from promoters p102, p69 and p59 using a flatbed PBR. In each case, cultures were kept in the dark until mid-log phase to prevent CvFAP activity during initial biomass accumulation. Actinic blue light was supplied after the addition of butyrate to the cultures, and headspace analysis for propane production was performed via continuous monitoring or manual sampling. This protocol is similar to recent studies that described the *in vivo* production of propane, butane and isobutane by non-fusion CvFAP variants in *Halomonas* (3, 11). Overall, the differences in growth rate between the three *Halomonas* constructs were less pronounced under PBR conditions as opposed to microtiter plate cultivation (see Supplementary Figures S2, S4 and S6). This was seen by similar culture optical densities at stationary phase for all three strains, and may be a consequence of differences in the culture conditions, such as aeration and/or blue light intensity.

**Figure 4.**
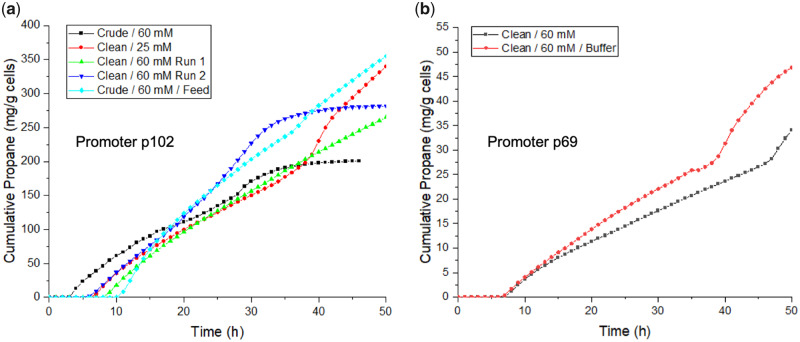
Cumulative propane production of *Halomonas* TQ10 expressing CvFAP_G462V_RFP under the control of constitutive promoters (**a**) p102 and (**b**) p69. General fermentation conditions: Cultures (400 ml) were grown in high salt glycerol medium pH 6.8 at 30°C with 60% stirring and an airflow rate of 1.21 l/min. Cultures were maintained in the dark until mid-log phase (OD_680_ ∼0.55). Butyric acid was added (25 or 60 mM; adjusted to pH 7.0) and the culture was illuminated with blue light (1625 µE), and maintained for ∼48 h. For the feed fermentation, once culture reached OD_680_ ∼0.55, continuous optical density maintenance was performed by feeding in the same culture medium containing the 60 mM butyrate, with continuous harvesting to maintain the culture volume. Propane concentration was determined by automated head space sampling using a Micro GC. Periodic culture samples (3 ml) were taken and analyzed for aqueous butyrate, glycerol and acetate concentrations by HPLC. Crude and clean glycerol is biodiesel waste glycerine and laboratory grade reagent, respectively. Culture growth and HPLC metabolite concentration data can be found in Supplementary Figures S2–S5. Data for the equivalent fermentation studies with *Halomonas* TQ10 expressing CvFAP_G462V_RFP under constitutive p59 promoter are found in Supplementary Figures S6 and S7.

A comparative study of propane production by *Halomonas* expressing p102-CvFAP_G462V_RFP showed fairly consistent cumulative propane production within the first 24–28 h (see Figure 4), in spite of variations in media composition between the five runs. This included *Halomonas* cultivation in the presence of ‘crude’ medium (3) containing seawater and biodiesel waste glycerine, designed to mimic more cost-effective scaled production conditions. In another case, less than half of the butyrate concentration was added, yet propane production was similar. The latter could be explained by observing that the concentration of butyrate did not decrease much over the fermentation, suggesting excessive levels had been added (see Supplementary Figure S4). In some cases, propane production diminished dramatically between 24 and 48 h, suggesting a loss of the biocatalytic plasmid and/or CvFAP (photo)inactivation. The average propane titers within the first 24 h were ∼100 mg/g cells (see Figure 4a). Implementation of a continuous culture regime to maintain culture density and medium composition yielded ∼350 mg propane/g cell in 2 days. This is comparable to batch fermentation studies of p102-CvFAP_G462V_ (no RFP) in *Halomonas*, which achieved around 250 mg propane/g cells within a similar time period (3).

*Halomonas* fermentation studies with ‘mid-range’ p69-CvFAP_G462V_RFP showed a near 10-fold decrease in propane titers (25–50 mg/g cells) than with the equivalent p102 promoter (see Figure 4b). This is similar to yields obtained in prior studies with IPTG-inducible CvFAP_G462I_ (no RFP), which was single site integrated into the *Halomonas* chromosome (3). Studies with ‘low-range’ p59-CvFAP_G462V_RFP generated propane titers too low for continuous monitoring, so manual headspace sampling was performed after allowing propane accumulation in the absence of aeration for 45 min (see Supplementary Figure S6). Titers were very low, achieving at best only 90 µg/g cells/day.

The transition from microtiter plate screening through to lab-scale fermentation has been successful in demonstrating a tuneable set of constitutive promoters in *Halomonas* (and *E. coli*), with minimal impact on cell growth. Given the limited subset of constitutive library promoters tested in *Halomonas*, further screening could dramatically improve the range of propane (or other biocatalytic) titers achievable in the absence of chemical inducers, thereby increasing the potential for scalable and commercially viable bio-processes. Further improvements could be obtained by employing a combinatorial approach, where selected promoters would be screened with a selection of RBSs designed specifically for the target protein. Existing scaled *Halomonas* biorefinery designs are considerably different from lab-scale cultivations (e.g. growth conditions and high cell densities), so further promoter optimization may be necessary to ensure a successful transitioning of new technologies from proof-of-principle demonstration to commercial application.

## 4. Conclusions

Commercialization of a recombinant bio-process is an iterative process, with step-wise improvements ‘chipping off’ economic barriers to success. One such barrier is the regulation of recombinant enzyme production (and activity) without the requirement for expensive chemical inducers. This has been achieved for CvFAP-dependent bio-propane production in *Halomonas* by the development of a series of tuneable constitutive promoters for modulating protein expression levels. Secondary control of *in vivo* CvFAP activity is possible by controlling (blue) light access to the culture. Therefore, the construction and screening of variable strength promoter libraries are one of many tools that may prove valuable in developing scalable recombinant bio-processes.

This study highlights that the relative strength of individual promoters is not fixed, but can be subject to both microbial host and gene-specific variation in response. The use of an easily detectable reporter gene within a genetically tractable microbial host enables rapid and high throughput identification of potentially useful promoter variants to generate small, focused libraries with a large dynamic range. This is especially important when available cloning strategies and/or enzymatic activity techniques are not amenable to rapid screening. This study also demonstrated the importance of considering promoter sequence length as a library variable, as a 5- to 8-fold difference in size led to dramatic increases or decreases in promoter strength in some cases, not previously shown in *Halomonas*. Overall, the demonstration of an *E. coli* and *Halomonas*-specific set of constitutive promoters takes us a step closer to commercialization of bio-propane production. Future applications using constitutive promoters within *Halomonas* (and other industrial hosts) will assist in the development of strategies for the production of biologically derived fuels and fine chemicals.

## Materials and resources 

Materials and resources described in the article are available from the authors under a Materials Transfer Agreement.

## Supplementary Material

ysaa022_Supplementary_Data
