# Supplementary material for: Promoter engineering for microbial bio-alkane gas production
Source: Synth Biol (Oxf). 2020 Oct 27;5(1):ysaa022. doi: 10.1093/synbio/ysaa022 (PMC7680561; doi:10.1093/synbio/ysaa022)
Supplement: ysaa022_Supplementary_Data [file ysaa022_supplementary_data.docx]

**Supplementary Data**

**Promoter Engineering for Microbial Bio-alkane Gas Production**

**Duangthip Trisrivirat,^1,2,3^ John M. X. Hughes,^1^ Robin Hoeven,^1^ Matthew Faulkner,^1^ Helen Toogood,^1^ Pimchai Chaiyen^2^ and Nigel S Scrutton^1,2*^**

^1^EPSRC/BBSRC Future Biomanufacturing Research Hub, BBSRC/EPSRC Synthetic Biology Research Centre SYNBIOCHEM Manchester Institute of Biotechnology and School of Chemistry, The University of Manchester, Manchester, M1 7DN, UK.

^2^School of Biomolecular Science and Engineering, Vidyasirimedhi Inistitute of Science and Technology (VISTEC), 555 Moo 1 Payupnai, Wangchan Valley, Rayong, Thailand 21210.

^3^Department of Biochemistry, Faculty of Science, Mahidol University, 272 Rama VI Road, Ratchathewi, Bangkok, Thailand 10400.

*Corresponding author.

**Keywords**: Constitutive promoter engineering, bio-propane, *Halomonas*, *Escherichia coli*.

**Table of Contents**

**Supplementary Figures**

**Figure 1:** Growth curves of *Halomonas* TQ10 expressing CvFAP_G462V_RFP **3**

**Figure 2:** Fermentation of *Halomonas* TQ10 expressing p102-CvFAP_G462V_RFP **4**

**Figure 3:** HPLC analysis of *Halomonas* TQ10 expressing p102-CvFAP_G462V_RFP **5**

**Figure 4:** Fermentation of *Halomonas* TQ10 expressing p69-CvFAP_G462V_RFP **6**

**Figure 5:** HPLC analysis of *Halomonas* TQ10 expressing p69-CvFAP_G462V_RFP **6**

**Figure 6:** Fermentation of *Halomonas* TQ10 expressing p59-CvFAP_G462V_RFP **7**

**Figure 7:** HPLC analysis of *Halomonas* TQ10 expressing p59-CvFAP_G462V_RFP **7**

**Supplementary Tables**

**Table 1:** Plasmid constructs used in the study **8**

**Table 2:** Oligonucleotide sequences for the assembly of DNA constructs **9**

**Table 3:** p-Porin-like constitutive promoter library variable region sequences **10**

**Table 4:** Promoter screening full data for *E. coli* expressing RFP **12**

**Table 5:** Promoter screening for *Halomonas* TQ10 expressing RFP **14**

**Table 6:** Partial promoter screening data for *E. coli* expressing RFP **15**

**Table 7:** Promoter screening for *Halomonas* TQ10 expressing CvFAP_G462V_RFP **16**

**Table 8:** Promoter screening for *E. coli* expressing CvFAP_G462V_RFP **17**

**Supplementary Data**

Plasmid

**References** **17**


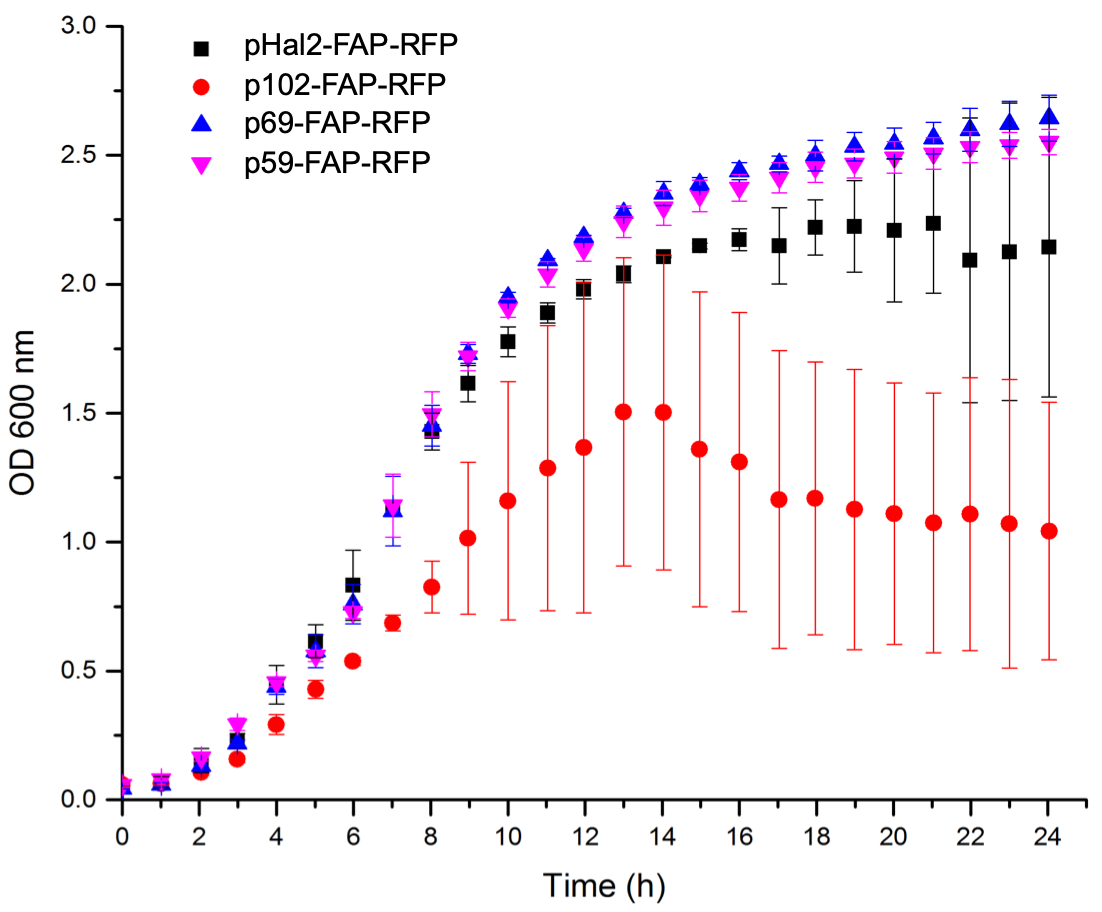


**Figure 1**. Growth curves of *Halomonas* TQ10 expressing CvFAP_G462V_RFP under the control of four different promoters. Cultures (1 mL) were grown in YTN6 medium containing 50 μg/ml spectinomycin in 2 mL Axygen^®^ 96-deep well plates, sealed with sterile gas permeable adhesive seals. The plates were incubated overnight at 30 ^o^C with 850 rpm agitation. Control wells contained either YTN6 media only or *Halomonas* TQ10 cultures of the IPTG-inducible pHal2-CvFAP_G462V_RFP. Replicate cultures (200 μL; starting OD_600 nm_ ~ 0.1) were set up in the same medium with spectinomycin in 96-well microtiter plates sealed with a moisture barrier seal. Cultures were incubated at 30 ^o^C with 300 rpm agitation in a CLARIOstar^®^ Plus Plate Reader. IPTG (0.1 mM) was added to the inducible cultures once OD_600 nm_ reached 0.55, and the incubation was continued overnight as before. The culture optical density was monitored every 5 minutes. Results are expressed as the mean of technical replicates, with error bars representing one standard deviation of the data.


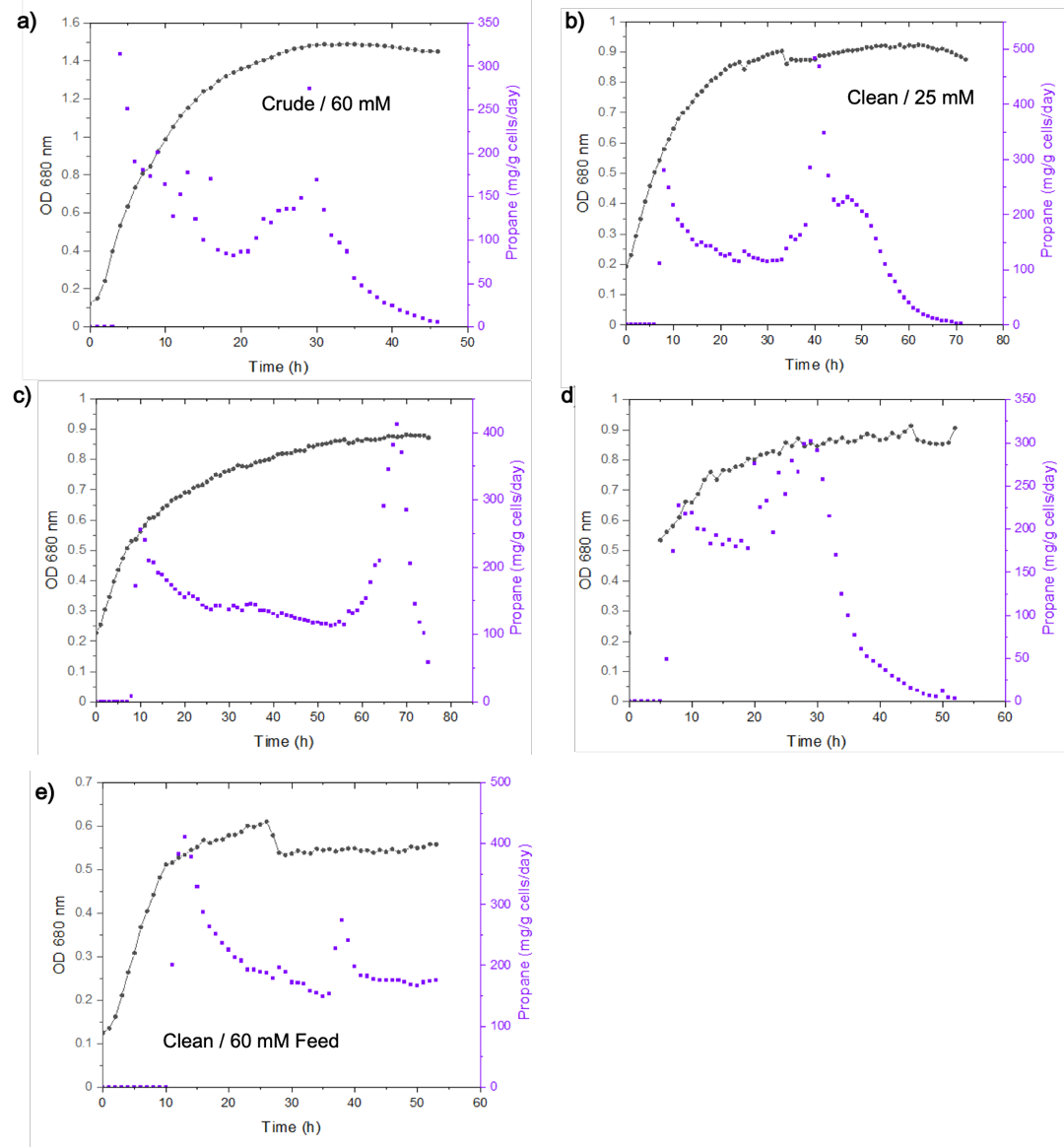


**Figure 2.** Fermentation runs of *Halomonas* TQ10 expressing CvFAP_G462V_RFP with a medium strength constitutive promoter p102. Cultures (400 mL) were grown in a flat panel photobioreactor in batch mode with high salt glycerol medium at pH 6.8 (5 g/L yeast extract, 1 g/L glycerol (crude or clean), 60 g/L NaCl, 50 μg/mL spectinomycin and 0.5 mL/L antifoam; 400 mL), pre-equilibrated at 30 °C with 60% stirring. An overnight starter culture (10 mL) of *Halomonas* TQ10 expressing CvFAP_G462V_RFP with the p102 promoter was added and the culture was maintained at 30 °C with an airflow rate of 1.21 L/min. Culture maintenance was performed with automated pH maintenance (sodium acetate), culture optical density monitoring and ambient room lighting until mid-log phase (OD_680_ ~ 0.55). Butyric acid was added (25 for run 1 and 60 mM for run 2; adjusted to pH 7.0) and the culture was illuminated with blue light (1625 μE), and maintained for ~48-72 h. For the feed fermentation, once culture reached OD680 ~ 0.55, continuous optical density maintenance was performed by feeding in the same culture medium containing the 60 mM butyrate, with continuous harvesting to maintain the culture volume. Propane concentration was determined by continuous monitoring at 15-minute intervals by a Micro GC. Crude and clean glycerol are biodiesel waste glycerine and laboratory grade reagent, respectively.


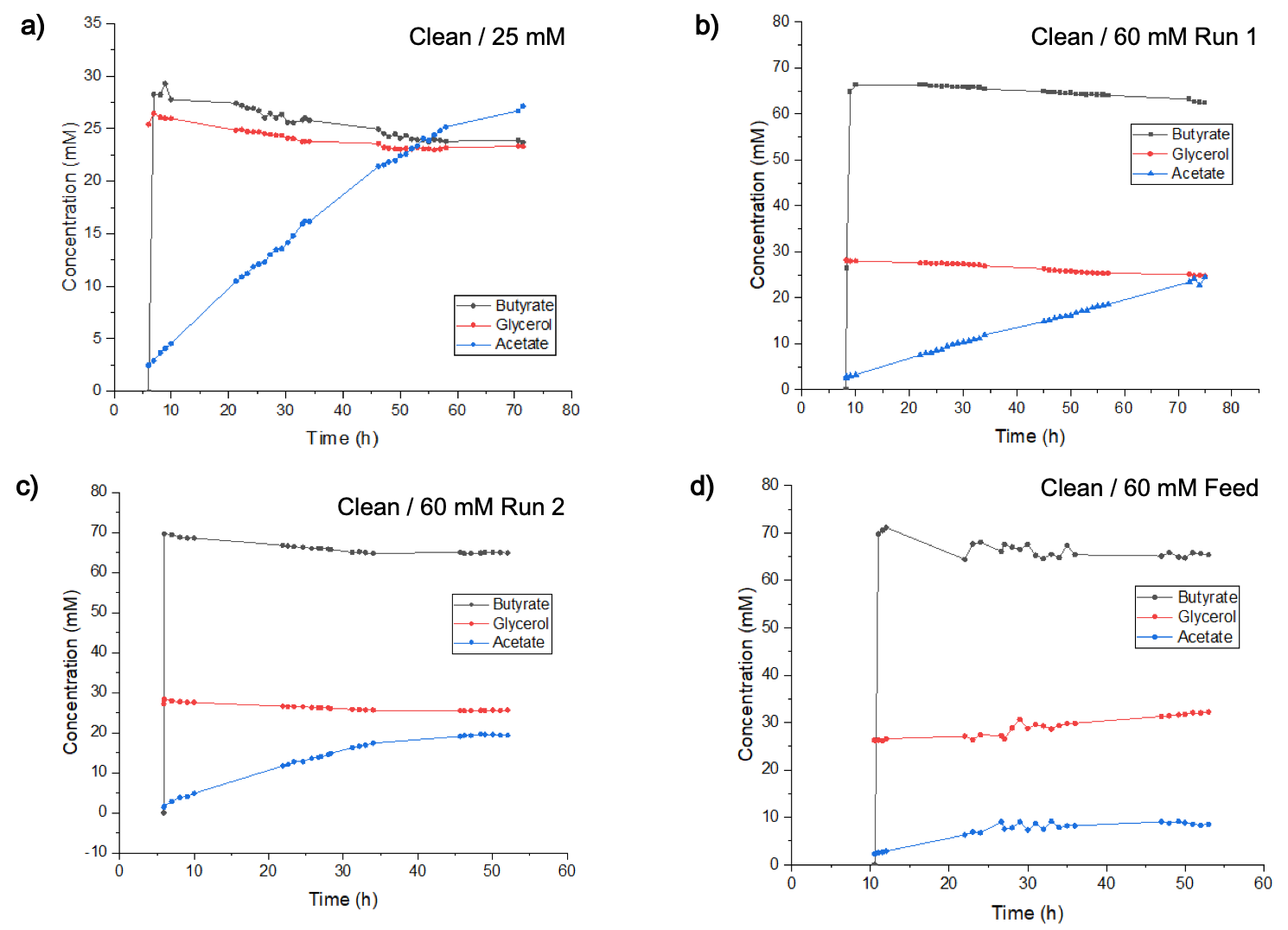


**Figure 3.** HPLC analysis of culture medium metabolites during fermentation runs of *Halomonas* TQ10 expressing CvFAP_G462V_RFP with a medium strength constitutive promoter p102. Culture conditions are described in the legend of Figure S2. The concentration of butyrate, glycerol and acetate in the culture medium were determined by HPLC using an Agilent 1260 Infinity HPLC with a 1260 refractive index detector (RID) and an Agilent Hi-Plex H column (300 x 7.7 mm; 5 mM H_2_SO_4_) as described previously (1).


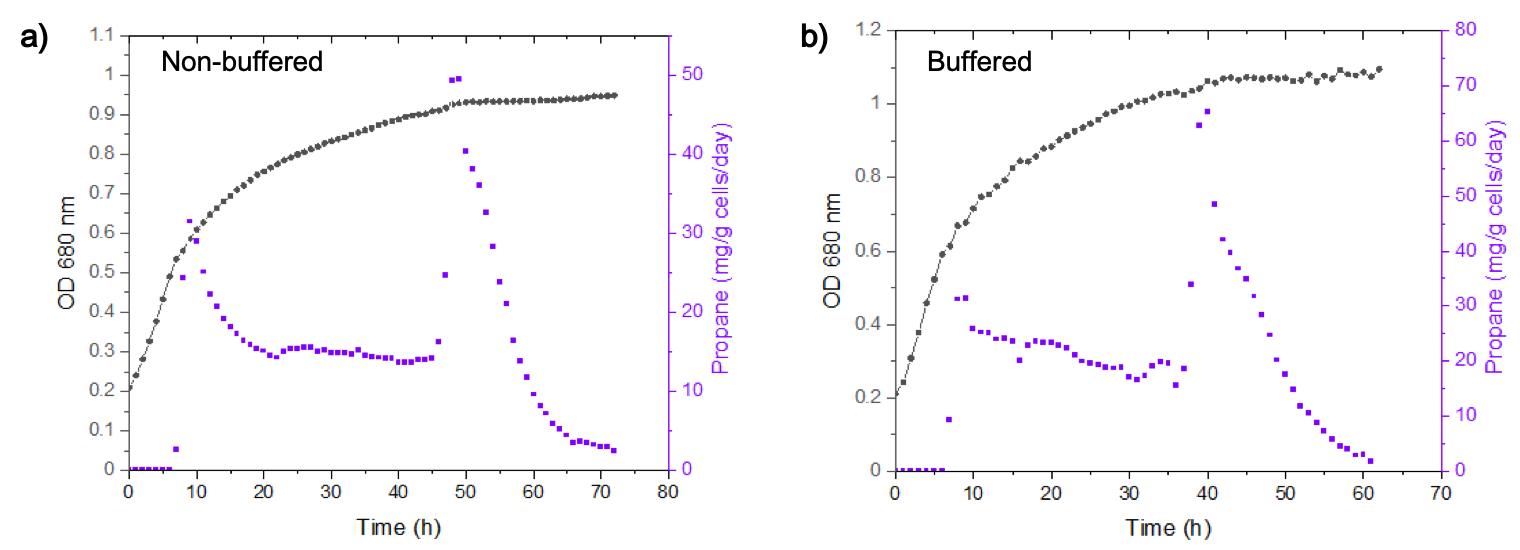


**Figure 4.** Fermentation of *Halomonas* TQ10 expressing CvFAP_G462V_RFP with constitutive promoter p69. Cultures (400 mL) were grown in a flat panel photobioreactor in batch mode with high salt glycerol medium at pH 6.8 (5 g/L yeast extract, 1 g/L glycerol, 60 g/L NaCl, 50 μg/mL spectinomycin and 0.5 mL/L antifoam; 400 mL), pre-equilibrated at 30 °C with 60% stirring. For the buffered culture, the medium contained 60 mM sodium phosphate buffer. An overnight starter culture (10 mL) of *Halomonas* TQ10 expressing CvFAP_G462V_RFP with the p69 promoter was added and the culture was maintained at 30 °C with an airflow rate of 1.21 L/min. Culture maintenance was performed with automated pH maintenance (sodium acetate), culture optical density monitoring and ambient room lighting until mid-log phase (OD_680_ ~ 0.55). Butyric acid was added (60 mM; adjusted to pH 7.0) and the culture was illuminated with blue light (1625 μE), and maintained for ~48-72 h. Propane concentration was determined by continuous monitoring at 15-minute intervals by a Micro GC.


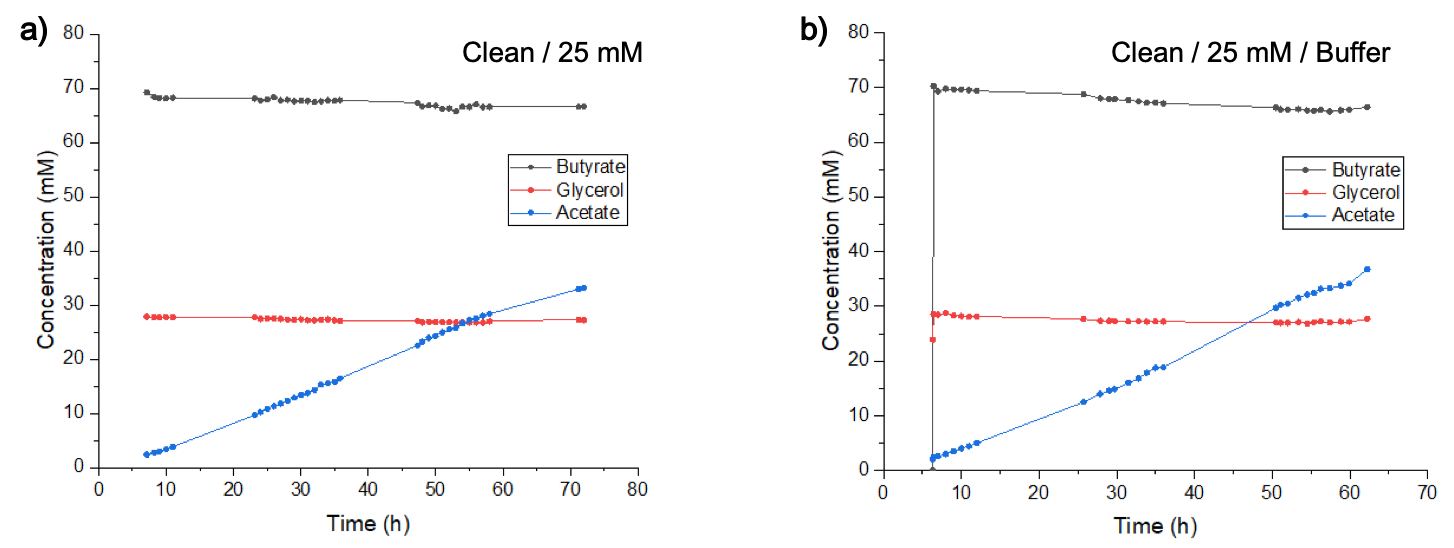


**Figure 5.** HPLC analysis of culture medium metabolites during fermentation runs of *Halomonas* TQ10 expressing CvFAP_G462V_RFP with a medium strength constitutive promoter p69. Culture conditions are described in the legend of Figure S4. The concentration of butyrate, glycerol and acetate in the culture medium were determined by HPLC using an Agilent 1260 Infinity HPLC with a 1260 refractive index detector (RID) and an Agilent Hi-Plex H column (300 x 7.7 mm; 5 mM H_2_SO_4_) as described previously (1).


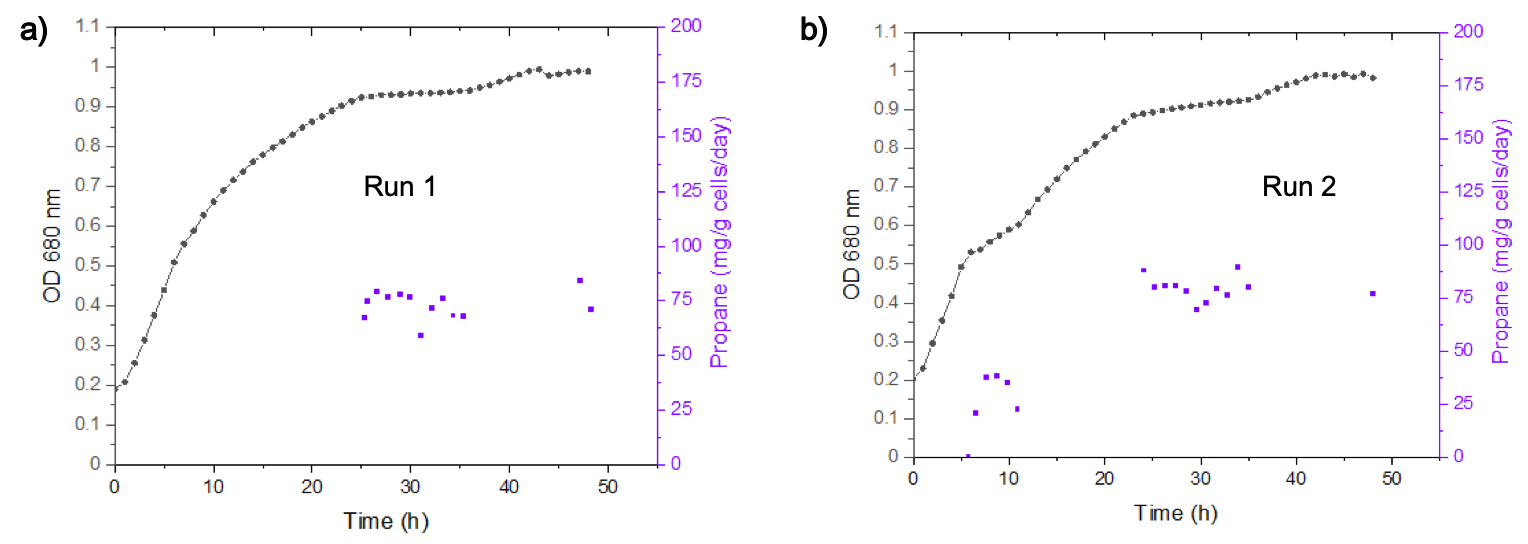


**Figure 6.** Fermentation of *Halomonas* TQ10 expressing CvFAP_G462V_RFP with constitutive promoter p59. Panels a) and b) represent duplicate cultivations performed under identical conditions. Cultures (400 mL) were grown in a flat panel photobioreactor in batch mode with high salt glycerol medium at pH 6.8 (5 g/L yeast extract, 1 g/L glycerol, 60 g/L NaCl, 50 μg/mL spectinomycin and 0.5 mL/L antifoam; 400 mL), pre-equilibrated at 30 °C with 60% stirring. An overnight starter culture (10 mL) of *Halomonas* TQ10 expressing CvFAP_G462V_RFP with the p59 promoter was added and the culture was maintained at 30 °C with an airflow rate of 1.21 L/min. Culture maintenance was performed with automated pH maintenance (sodium acetate), culture optical density monitoring and ambient room lighting until mid-log phase (OD_680_ ~ 0.55). Butyric acid was added (25 mM; adjusted to pH 7.0) and the culture was illuminated with blue light (1625 μE), and maintained for ~48-72 h. Periodically the air flow was stopped for 45 minutes prior to manual headspace sampling to allow the propane levels to accumulate. Propane concentration was determined by continuous monitoring at 15 minute intervals by a Micro GC.


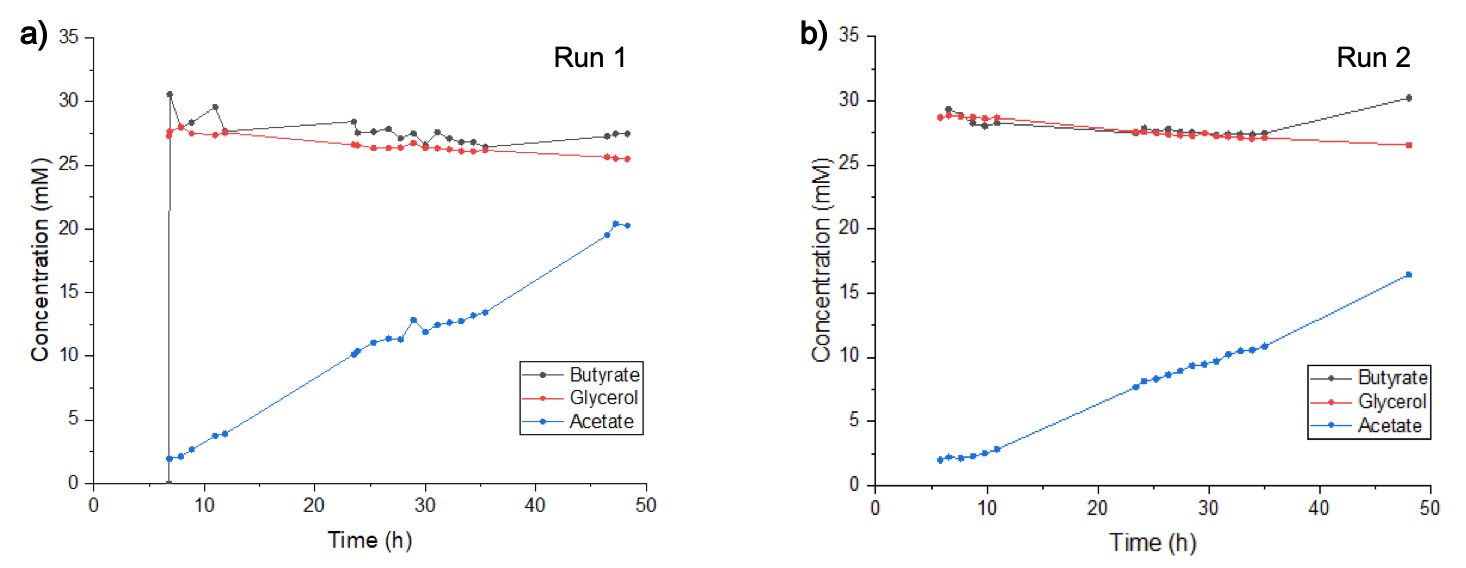


**Figure 7.** HPLC analysis of culture medium metabolites during fermentation runs of *Halomonas* TQ10 expressing CvFAP_G462V_RFP with a medium strength constitutive promoter p59. Culture conditions are described in the legend of Figure S4. The concentration of butyrate, glycerol and acetate in the culture medium were determined by HPLC using an Agilent 1260 Infinity HPLC with a 1260 refractive index detector (RID) and an Agilent Hi-Plex H column (300 x 7.7 mm; 5 mM H_2_SO_4_) as described previously (1).

| Table 1 Plasmid constructs used in this study | | |  | |
| --- | --- | --- | --- | --- |
| Construct | Promoter-Gene(s) | Plasmid | Antibiotic | References |
| pHal2-CvFAP_G462V_ | *T7like*-CvFAP_G462V_ | pHalSEVA | Kan^R^, Sm^R^ | (1) |
| pHal2-RFP | *T7like*-RFP | pHalSEVA | Kan^R^, Sm^R^ | This study |
| pBbA1a-RFP | *pTrc-*RFP | pBbA1a | Amp^R^ | (2) |
| pBbA5a-RFP | *placUV5-*RFP | pBbA5a | Amp^R^ | (2) |
| pHalT7P7-CvFAP_G462V_ | *T7like*-*pP7-*CvFAP_G462V_ | pHalSEVA | Kan^R^, Sm^R^ | This study |
| pHal7-CvFAP_G462V_ | *pP7-*CvFAP_G462V_ | pHalSEVA | Kan^R^, Sm^R^ | This study |
| pHalT7P7-RFP | *T7like*-*pP7-*RFP | pHalSEVA | Kan^R^, Sm^R^ | This study |
| pHal7-RFP-STag | *pP7-*RFP with non-functional S-Tag | pHalSEVA | Kan^R^, Sm^R^ | This study |
| pHal7-RFP | *pP7-*RFP | pHalSEVA | Kan^R^, Sm^R^ | This study |
| pHal7 | *pP7* (no gene control) | pHalSEVA | Kan^R^, Sm^R^ | This study |
| pHalV-RFP | *pPVariable-*RFP library | pHalSEVA | Kan^R^, Sm^R^ | This study |
| pHal7-FAP_G462V_RFP | *pP7-*CvFAP_G462V_RFP | pHalSEVA | Kan^R^, Sm^R^ | This study |
| pHal7-FAP_G462V_RFP | *pP7-*CvFAP_G462V_RFP fusion | pHalSEVA | Kan^R^, Sm^R^ | This study |
| pHal2-FAP_G462V_RFP | *T7like-*CvFAP_G462V_RFP fusion | pHalSEVA | Kan^R^, Sm^R^ | This study |
| pHalV-FAP_G462V_RFP | *pPVariable-*CvFAP_G462V_RFP fusion library | pHalSEVA | Kan^R^, Sm^R^ | This study |
| Antibiotic resistance: Kan^R^, kanamycin; Sm^R^, spectinomycin. Promoters: T7like, MmP1 P_T7_-like-promoter; pP7, constitutive porin-like promoter; pPVariable, constitutive porin-like library of promoters. pHalSEVA: modified pSEVA441containing the MmP1 P_T7_-like-promoter, an *Nco*I restriction site and a pET21b-like Shine-Delgarno sequence upstream of the start codon.(1) | | | | |

| **Table 2** Oligonucleotide sequences for the assembly of DNA constructs. | | |
| --- | --- | --- |
| **Stage** | **Oligonucleotide sequence 5’ to 3’** | **Template DNA** |
| *Construction of pHalT7P7-CvFAP_G462V_ by overlap extension PCR and In-Fusion cloning* | | |
| Vector opening  (Two pairs of overlapping oligos) | TTTACTAGAGAAAGAGGAGAAATACTAGATGGCCAGCGCAGTTGAAGATATTCG & TGAGACTTTACTAGAGAAAGAGGAGAAATACTAGATGGC  GTATTTTACGCAATAGAAAAACCCTATAATGCCACAAATATAATTAACTCATGG & AACTCTATACAACTAATGTATTTTACGCAATAGAAAAAC CCTATAATGCC | pHal2-CvFAP_G462V_ |
| *Construction of pHal7-RFP and removal of the obsolete P_T7_-like-promoter and S-Tag by In-Fusion cloning* | | |
| Vector opening  RFP PCR  pT7-like removal  S-Tag removal | CTCGAGTCTGGTAAAGAAACCGCTGC  CTAGTATTTCTCCTCTTTCTCTAGTAAAGTCTCAAAC  GAGGAGAAATACTAGATGGCGAGTAGCGAAGACGTTATCAAAG  TTTACCAGACTCGAGTTAAGCACCGGTGGAGTGACGACC  GGTTTTTCTATTGCGTACAACCGATAAAGGTATAGAGTTTG  CGCAATAGAAAAACCTCCTGTGTGAAATTGTTATCCGC  TTAACCTAGGCTGCTGCCACCGCTGAGCAATAAAGC  AGCAGCCTAGGTTAATTAAGCACCGGTGGAGTGACG | pHalT7P7-CvFAP_G462V_  pBbE1c-RFP  pHalT7P7-RFP  pHal7-RFP-STag |
| *Construction of pHal2-RFP by In-Fusion cloning* | | |
| Vector opening  RFP PCR | CTCGAGTCTGGTAAAGAAACCGCTGC  GGTATATCTCCTTCTTAAAGTTAAACAAACTAGTATTTCTC  AGAAGGAGATATACCATGGCGAGTAGCGAAGACGTTATC  TTTACCAGACTCGAGTTAAGCACCGGTGGAGTGACGAC | pHal2-CvFAP_G462V_  pBbE1c-RFP |
| *Construction of pHal7 (RFP removal) by In-Fusion cloning* | | |
| Vector opening | GAGAAATACTAGCTCGAGTCTGGTAAAGAAACCGCTGC  GAGCTAGTATTTCTCCTCTTTCTCTAGTAAAGTCTCAAACTC | pHal7-RFP |
| *Random mutagenesis and In-Fusion cloning of pHal7-RFP at the 14 bp variable region of the porin promoter* | | |
| Vector opening | NNNNNNNGTATAGAGTTTGAGACTTTACTAGAGAAAGAG  NNNNNNNACGCAATAGAAAAACCTCCTGTGTGAAATTG | pHal7-RFP |
| *Construction of pHal7-FAP_G462V_RFP dual construct by In-Fusion cloning* | | |
| Vector opening  RFP PCR | ATTTAGCTGTCCTCCTTATGCTGCAACGGTTGCCGG  TTAACCTAGGCTGCTGCCACCGCTGAGCAATAAAGC  GGAGGACAGCTAAATGGCGAGTAGCGAAGACGTTATC  AGCAGCCTAGGTTAATTAAGCACCGGTGGAGTGACG | pHalT7P7-CvFAP_G462V_  pHal7-RFP |
| *Construction of pHal7-FAP_G462V_RFP fusion by stop codon removal and linker^a^ addition* | | |
| Vector opening | GTTCTGCGGCCGGTTCTGGCGAATTTATGGCGAGTAG  AACCGGCCGCAGAACCCGCAGAACCTGCTGCAAC | pHal7-FAP_G462V_RFP |
| *Construction of pHal2-FAP_G462V_RFP fusion by In-Fusion cloning* | | |
| Vector opening  CvFAP_G462V_RFP PCR | ATGGCGAGTAGCGAAGACGTTATCAAAG  GGTATATCTCCTTCTTAAAGTTAAACAAACTAGTATTTCTCC  AGAAGGAGATATACCATGGCCAGCGCAGTTGAAGATATTC  TTCGCTACTCGCCATAAATTCGCCAGAACCGGCCGC | pHal2_RFP_inducible  pHal7-FAP_G462V_RFP |
| *Construction of pHalV-FAP_G462V_RFP library by In-Fusion cloning* | | |
| Vector opening  CvFAP_G462V_RFP PCR | TTAACCTAGGCTGCTGCCACCGCTGAGCAATAAAGC  CTAGTATTTCTCCTCTTTCTCTAGTAAAGTCTCAAAC  GAGGAGAAATACTAGATGGCCAGCGCAGTTGAAGATATTCG  AGCAGCCTAGGTTAATTAAGCACCGGTGGAGTGACG | pHalV-RFP  pHal7-FAP_G462V_RFP |
| ^a^Linker DNA sequence (GGTTCTGCGGGTTCTGCGGCCGGTTCTGGCGAATTT) between CvFAP_G462V_ and RFP. | | |

| Table 3 p-Porin-like constitutive promoter library variable region sequences | | |
| --- | --- | --- |
| Clone | Variable region sequence 5’ to 3’ | Length (bp) |
| Porin  P7^a^ | TTGCGTTCACTGGAATCCCAGTATAGAGTTTGAGAC*TTTACTAGAGAAAGAGGAGAAATACTAG*  TTGCGTACAACCGATAAAGGTATAGAGTTTGAGAC*TTTACTAGAGAAAGAGGAGAAATACTAG* | 14  13 |
| 1 | GTATTCACTCTCCA | 14 |
| 2 | CAAACCGTAAATA | 13 |
| 3 | GTTTATGCAGAGCA | 14 |
| 4 | ACGCCGGGCAGAAT | 14 |
| 5 | CACCCTCAGTGCTG | 14 |
| 6 | GGTGGAATTATTAT | 14 |
| 7 | TTGACATTCATTCT | 14 |
| 8 | AAGTTTTTTTGGCG | 14 |
| 13 | TGTTACTCACATGT | 14 |
| 14 | ATTTGGTTACAGGG | 14 |
| 15 | ACAATTCTCTTGAA | 14 |
| 16 | CGAAATGTGTGAGG | 14 |
| 17 | GACGATTTAAAGTGGTATAGAGTTTGAGACTTTACTAGAGAAAGAGAGAGGATAACGCAATTTCACACAGGAGGTTTTTCTATTGCGTCCGCGCCGTACCTC | 102 |
| 18 | CTCGATTAATGCCA | 14 |
| 19 | ACCCTTTGTGCTAG | 14 |
| 20 | CCGGTTCTTACTCC | 14 |
| 21 | AAAATGAGTGTGAG | 14 |
| 22 | AGTCAGCAGCTTGT | 14 |
| 24 | ACACAGTTAGTCAT | 14 |
| 25 | TTTGCATGGGTGA | 13 |
| 27 | ATTAAACCCTTGAA | 14 |
| 29 | GCCTCCAAACTTAT | 14 |
| 31 | GGTTTATCATCCGT | 14 |
| 32 | CCGATAAGTGTTCA | 14 |
| 34 | AACCTCCCCGCGAG | 14 |
| 35 | CAACAGATCTGAAG | 14 |
| 37 | ACACTATATCACGG | 14 |
| 39 | ACCTTTTGACTCAT | 14 |
| 41 | ACCTTTGCAAGCAT | 14 |
| 42 | ACATCAAAGCTAAAGTATAGAGTTTGAGACTTTACTAGAGAAAGAGGAGAAATAAAAGCGGATAACAATTTCACACAGGAGGTTTTTCTATTGCGTTAGGTATACGGAG | 110 |
| 43 | AC | 2 |
| 44 | CCCTCCTCAAAGG | 13 |
| 47 | ATCCTTTTCTTCAT | 14 |
| 49 | GCGAATTTGAAGGT | 14 |
| 50 | ATCTACATGGACGG | 14 |
| 53 | CCTGTGTTAGTGAG | 14 |
| 54 | GTTCTTAGTAGCGTGTATAGAGTTTGAGACTTTACTAGAATTAAAGCGGATAACAATTTCACACAGGAGGTTTTTCTATTGCGTACCCATCGCTAGCT | 98 |
| 58 | CCCTATCTAAAGCT | 14 |
| 59 | TGGCCACTGAGCA | 13 |
| 62 | TCTATTATGGGTG | 13 |
| 69 | GCTCATTGGCCAAT | 14 |
| 71 | GGACTCCAGCTATT | 14 |
| 72 | CTCAATCAGAGTAT | 14 |
| 77 | GCATGCTAGTAGGA | 14 |
| 78 | TATGCAAAAG | 10 |
| 79 | CGTCTGATGTAGGC | 14 |
| 80 | GTCTCCGGAGTAAG | 14 |
| 85 | GCAGAATCGGATGC | 14 |
| 87 | ATGAAAATGGCACC | 14 |
| 89 | ATGTCATGACCTGC | 14 |
| 91 | TACTTCTGATGGCG | 14 |
| 92 | CTGTTATCGCCGCT | 14 |
| 93 | ACCTCGATGAGTCT | 14 |
| 97 | TATGGTACCTGGGT | 14 |
| 98 | CCGACACAGGCCCC | 14 |
| 99 | GATACTTTTCTAGA | 14 |
| 100 | AACTACAGTGGGGG | 14 |
| 101 | ATGCAGTTTGAGAT | 14 |
| 102 | CCTGATCGTAGTGC | 14 |
| 103 | GACTTTAAATGGGG | 14 |
| 107 | ACCTGGTTGTGAT | 13 |
| 109 | ACACAACTCAGTAT | 14 |
| 112 | ACTCCATAGTGGAT | 14 |
| 114 | CCAACCTTCCAGGGTATAGAGTTTGAGACTTTACTAGAGAAAGAGGAGAAATACTAAAGCGGATAACAATTTCACACAGGAGGTTTTTCTATTGCGTACTTGAAGTGAATA | 111 |
| 116 | CCATCTA | 7 |
| 120 | TCATTAACA | 9 |
| 121 | CCTTGGGCAGA | 11 |
| 123 | TTTCCTTAGGCGTTT | 15 |
| 124 | TACTCAAGCCGATT | 14 |
| 125 | CTCTCCGTTG | 10 |
| 129 | AATCAGCAGCTTGT | 14 |
| ^a^Full sequence of the inserted P7 *P_porin_*-like promoter (pHal7). Underlined: -35 and -10 boxes; bold: variable region; *italics*: Shine Dalgarno and spacer sequence. The first sequence is the original unmodified 40-bp *P_porin_* sequence obtained from (3). | | |

| Table 4. Screening data for RFP-containing constitutive library clones. | | |  |
| --- | --- | --- | --- |
| Clone/Promoter | Optical Density  (OD_600 nm_) | Fluorescence Intensity  (FI) | RFU  (FI/OD_600 nm_) |
| *Control plasmids* | | | |
| pHal7 (empty vector) | 1.070 ± 0.310 | N/A | N/A |
| pBbA1a-RFP uninduced | 1.047 ± 0.208 | 3103 ± 561 | 2992 ± 312 |
| pBbA5a-RFP uninduced | 1.746 ± 0.269 | 3397 ± 4090 | 1776 ± 1632 |
| pHal2-RFP uninduced | 0.961 ± 0.263 | 276 ± 185 | 284 ± 191 |
| pBbA1a-RFP induced | 1.534 ± 0.451 | 27166 ± 40805 | 13963 ± 15766 |
| pBbA5a-RFP induced | 1.265 ± 0.368 | 5229 ± 10533 | 3731 ± 6880 |
| pHal2-RFP induced | 0.821 ± 0.133 | 219 ± 152 | 275 ± 224 |
| pHal7-RFP | 1.102 ± 0.208 | 3129 ± 1424 | 3028 ± 1860 |
| *Constitutive library clones* | | | |
| 37 | 2.397 ± 1.714 | 168851 ± 128793 | 64055 ± 13886 |
| 114 | 1.753 ± 1.373 | 120201 ± 101181 | 63230 ± 11924 |
| 3 | 1.303 ± 0.993 | 84219 ± 67830 | 62396 ± 9932 |
| 17 | 0.065 ± 0.039 | 3161 ± 1799 | 50069 ± 3784 |
| 27 | 0.932 ± 0.102 | 40096 ± 8605 | 43126 ± 8528 |
| 124 | 0.290 ± 0.378 | 12070 ± 15054 | 43003 ± 5678 |
| 8 | 1.351 ± 0.403 | 57038 ± 26077 | 41029 ± 6872 |
| 7 | 0.739 ± 0.073 | 28294 ± 1643 | 38459 ± 3115 |
| 85 | 1.407 ± 1.029 | 46187 ± 32833 | 33176 ± 924 |
| 21 | 0.966 ± 0.061 | 31699 ± 2234 | 32994 ± 4401 |
| 16 | 1.471 ± 0.562 | 47725 ± 17489 | 32630 ± 1716 |
| 34 | 0.719 ± 0.074 | 21644 ± 1612 | 30168 ± 1244 |
| 35 | 0.820 ± 0.079 | 23493 ± 562 | 28827 ± 2833 |
| 87 | 0.988 ± 0.254 | 26530 ± 1498 | 28080 ± 7119 |
| 18 | 1.048 ± 0.392 | 28675 ± 11981 | 27554 ± 5518 |
| 15 | 1.065 ± 0.119 | 28384 ± 1950 | 26980 ± 4682 |
| 4 | 1.331 ± 0.420 | 37117 ± 20488 | 26854 ± 6992 |
| 71 | 0.701 ± 0.289 | 17027 ± 5120 | 25271 ± 3766 |
| 99 | 0.810 ± 0.234 | 21121 ± 10351 | 25179 ± 4734 |
| 20 | 0.936 ± 0.359 | 23024 ± 8858 | 24703 ± 4975 |
| 6 | 0.919 ± 0.049 | 21958 ± 4412 | 23869 ± 4304 |
| 22 | 0.685 ± 0.112 | 12669 ± 2459 | 18447 ± 599 |
| 102 | 1.498 ± 0.223 | 22589 ± 1818 | 15381 ± 3322 |
| 93 | 0.973 ± 0.129 | 14320 ± 2797 | 14739 ± 2190 |
| 77 | 0.912 ± 0.177 | 13131 ± 1803 | 14673 ± 2654 |
| 129 | 1.448 ± 0.365 | 19745 ± 5867 | 13518 ± 763 |
| 19 | 1.205 ± 0.182 | 14888 ± 2279 | 12683 ± 3494 |
| 103 | 0.770 ± 0.089 | 9552 ± 1360 | 12475 ± 1918 |
| 24 | 0.957 ± 0.088 | 11669 ± 1482 | 12218 ± 1301 |
| 25 | 0.920 ± 0.023 | 11122 ± 1848 | 12117 ± 2306 |
| 41 | 0.918 ± 0.013 | 10236 ± 1813 | 11129 ± 1825 |
| 62 | 0.678 ± 0.102 | 6834 ± 251 | 10206 ± 1276 |
| 79 | 0.826 ± 0.233 | 8257 ± 2939 | 9872 ± 1023 |
| 47 | 1.363 ± 0.219 | 12251 ± 1214 | 9064 ± 976 |
| 109 | 1.351 ± 0.105 | 10304 ± 1190 | 7610 ± 305 |
| 54 | 0.944 ± 0.033 | 7092 ± 1340 | 7489 ± 1143 |
| 31 | 1.209 ± 0.316 | 8873 ± 1628 | 7463 ± 737 |
| 42 | 0.903 ± 0.116 | 5882 ± 562 | 6532 ± 347 |
| 101 | 1.299 ± 0.36 | 8363 ± 2239 | 6477 ± 1038 |
| 39 | 0.940 ± 0.168 | 5739 ± 539 | 6173 ± 588 |
| 98 | 1.508 ± 0.342 | 8815 ± 1595 | 5892 ± 346 |
| 49 | 0.741 ± 0.083 | 3055 ± 363 | 4122 ± 33 |
| 116 | 0.671 ± 0.211 | 2608 ± 651 | 3960 ± 527 |
| 112 | 1.231 ± 0.286 | 4663 ± 1026 | 3801 ± 252 |
| 69 | 0.655 ± 0.150 | 2129 ± 543 | 3241 ± 87 |
| 92 | 0.973 ± 0.094 | 2644 ± 362 | 2725 ± 381 |
| 44 | 0.797 ± 0.048 | 1411 ± 290 | 1788 ± 459 |
| 123 | 1.111 ± 0.366 | 1642 ± 391 | 1557 ± 433 |
| 59 | 1.094 ± 0.198 | 1080 ± 116 | 1022 ± 301 |
| 107 | 0.778 ± 0.172 | 203 ± 44 | 261 ± 7 |
| 120 | 0.950 ± 0.169 | 122 ± 15 | 129 ± 7 |
| 121 | 0.911 ± 0.059 | 93 ± 10 | 103 ± 16 |
| *Additional constitutive library clones screened showing high variability* | | | |
| 32 | 1.060 ± 0.286 | 61488 ± 35761 | 55476 ± 18785 |
| 14 | 0.056 ± 0.050 | 2585 ± 2148 | 49159 ± 13592 |
| 72 | 0.014 ± 0.006 | 525 ± 737 | 45513 ± 68700 |
| 91 | 1.144 ± 0.402 | 49767 ± 23712 | 45286 ± 21913 |
| 50 | 2.410 ± 0.877 | 118239 ± 122681 | 42196 ± 29903 |
| 5 | 1.597 ± 0.126 | 63471 ± 33810 | 39992 ± 21934 |
| 1 | 1.531 ± 0.472 | 53053 ± 4263 | 37539 ± 14777 |
| 29 | 1.287 ± 0.617 | 52237 ± 32935 | 37206 ± 10628 |
| 2 | 1.565 ± 0.229 | 54238 ± 24838 | 35860 ± 19732 |
| 58 | 1.216 ± 0.402 | 37685 ± 6839 | 35463 ± 20707 |
| 53 | 1.019 ± 0.277 | 30605 ± 13763 | 31493 ± 18185 |
| 97 | 1.470 ± 0.155 | 30439 ± 7939 | 21262 ± 8013 |
| 80 | 0.099 ± 0.099 | 2440 ± 3486 | 19669 ± 15180 |
| 100 | 1.205 ± 0.355 | 22021 ± 2290 | 19579 ± 6663 |
| 89 | 0.776 ± 0.256 | 16099 ± 11907 | 18967 ± 8131 |
| 13 | 0.830 ± 0.159 | 12961 ± 12176 | 17893 ± 18846 |
| 78 | 0.017 ± 0.002 | 4 ± 3 | 224 ± 214 |
| 43 | 0.826 ± 0.122 | 50 ± 11 | 63 ± 22 |
| 125 | 1.081 ± 0.273 | 42 ± 7 | 41 ± 14 |
| Initial cultures (1 mL) were grown overnight in LB medium containing 50 μg/ml kanamycin at 30 °C with 850 rpm agitation in 2 mL 96-deep well plates with gas permeable seals. Replicate cultures (200 μL; starting OD_600 nm_ ~ 0.1) were set up in the same medium in 96-well microtiter plates with a moisture barrier seal. Cultures were incubated at 30 °C with 300 rpm agitation in a microtiter plate reader. IPTG (0.1 mM) was added to the inducible cultures once OD_600 nm_ reached 0.55, and the incubation was continued overnight as before. Both the culture optical density and relative RFP fluorescence intensity (RFI) were monitored every 5 minutes (excitation and emission wavelengths of 584 and 607 nm, respectively). Results are expressed as the mean of the relative fluorescence units (RFU), which is fluorescence intensity per OD_600 nm_, with error bars representing one standard deviation of the data. | | | |

| Table 5 Comparative performance of selected constitutive promoters for RFP expression in *Halomonas*. | | | |
| --- | --- | --- | --- |
| Clone/Promoter | Optical Density  (OD_600 nm_) | Fluorescence Intensity  (FI) | RFU  (FI/OD_600 nm_) |
| pHal7 (empty vector) | 2.307 ± 0.025 | ND | NA |
| pBbA1a-RFP uninduced | 1.612 ± 0.312 | ND | NA |
| pBbA5a-RFP uninduced | 1.988 ± 0.062 | ND | NA |
| pHal2-RFP uninduced | 1.733 ± 0.202 | ND | NA |
| pBbA1a-RFP induced | 1.880 ± 0.040 | ND | NA |
| pBbA5a-RFP induced | 1.832 ± 0.133 | ND | NA |
| pHal2-RFP induced | 2.169 ± 0.050 | 4409 ± 436 | 2030 ± 155 |
| pHal7-RFP | 2.243 ± 0.064 | 2 ± 10 | 1 ± 5 |
| 17 | 1.462 ± 0.032 | 1706 ± 17 | 1167 ± 22 |
| 3 | 2.266 ± 0.004 | 2552 ± 71 | 1126 ± 30 |
| 13 | 2.017 ± 0.154 | 2193 ± 499 | 1079 ± 160 |
| 32 | 1.974 ± 0.081 | 1981 ± 160 | 1003 ± 42 |
| 124 | 1.563 ± 0.057 | 1437 ± 58 | 919 ± 17 |
| 85 | 2.311 ± 0.027 | 2068 ± 84 | 895 ± 26 |
| 14 | 1.665 ± 0.406 | 1453 ± 626 | 848 ± 149 |
| 102 | 1.874 ± 0.080 | 1305 ± 90 | 696 ± 26 |
| 99 | 1.919 ± 0.123 | 949 ± 89 | 495 ± 26 |
| 100 | 2.030 ± 0.139 | 868 ± 152 | 425 ± 46 |
| 8 | 2.147 ± 0.146 | 827 ± 146 | 383 ± 44 |
| 15 | 1.673 ± 0.117 | 570 ± 41 | 341 ± 4 |
| 114 | 1.574 ± 0.071 | 490 ± 845 | 311 ± 535 |
| 103 | 1.734 ± 0.170 | 418 ± 71 | 240 ± 18 |
| 24 | 1.551 ± 0.024 | 345 ± 6 | 223 ± 6 |
| 69 | 2.174 ± 1.071 | 518 ± 578 | 195 ± 132 |
| 112 | 1.514 ± 0.010 | 247 ± 14 | 163 ± 9 |
| 37 | 1.884 ± 0.102 | 275 ± 33 | 146 ± 10 |
| 31 | 2.244 ± 0.143 | 322 ± 58 | 143 ± 16 |
| 44 | 1.688 ± 0.187 | 23 ± 2 | 14 ± 3 |
| 59 | 1.474 ± 0.025 | 10 ± 6 | 7 ± 4 |
| 34 | 2.091 ± 0.118 | ND | NA |
| Initial cultures (1 mL) were grown overnight in YTN6 medium containing 50 μg/ml spectinomycin at 30 °C with 850 rpm agitation in 2 mL 96-deep well plates with gas permeable seals. Replicate cultures (200 μL; starting OD_600 nm_ ~ 0.1) were set up in the same medium in 96-well microtiter plates with a moisture barrier seal. Cultures were incubated at 30 °C with 300 rpm agitation in a microtiter plate reader. IPTG (0.1 mM) was added to the inducible cultures once OD_600 nm_ reached 0.55, and the incubation was continued overnight as before. Both the culture optical density and relative RFP fluorescence intensity (RFI) were monitored every 5 minutes (excitation and emission wavelengths of 584 and 607 nm, respectively). Results are expressed as the mean of the relative fluorescence units (RFU), which is fluorescence intensity per OD_600 nm_, with error bars representing one standard deviation of the data; ND: none detected; NA: not applicable. | | | |

| Table 6 Comparative performance of selected constitutive promoters for RFP expression in *E. coli*. | | | |
| --- | --- | --- | --- |
| Clone/Promoter | Optical Density  (OD_600 nm_) | Fluorescence Intensity  (FI) | RFU  (FI/OD_600 nm_) |
| pHal7 (empty vector) | 0.945 ± 0.264 | ND | NA |
| pBbA1a-RFP uninduced | 1.014 ± 0.048 | 2759 ± 204 | 2719 ± 72 |
| pBbA5a-RFP uninduced | 1.616 ± 0.112 | 1697 ± 81 | 1052 ± 54 |
| pHal2-RFP uninduced | 1.003 ± 0.078 | 221 ± 33 | 220 ± 33 |
| pBbA1a-RFP induced | 1.252 ± 0.247 | 8112 ± 2368 | 6401 ± 643 |
| pBbA5a-RFP induced | 0.817 ± 0.182 | 1255 ± 166 | 1555 ± 137 |
| pHal2-RFP induced | 0.978 ± 0.085 | 200 ± 40 | 204 ± 31 |
| pHal7-RFP | 1.275 ± 0.091 | 2231 ± 390 | 1741 ± 189 |
| 17 | 0.058 ± 0.022 | 1814 ± 696 | 36932 ± 22712 |
| 3 | 0.441 ± 0.719 | 9643 ± 10462 | 97283 ± 71438 |
| 13 | 0.830 ± 0.159 | 12961 ± 12176 | 17983 ± 18846 |
| 32 | 1.146 ± 0.138 | 63500 ± 16904 | 56280 ± 16959 |
| 124 | 0.028 ± 0.002 | 902 ± 372 | 32772 ± 15703 |
| 85 | 1.283 ± 0.466 | 89328 ± 28786 | 70261 ± 2699 |
| 14 | 0.056 ± 0.050 | 2585 ± 2148 | 49159 ± 13592 |
| 102 | 1.019 ± 0.232 | 20400 ± 3262 | 20258 ± 1883 |
| 99 | 1.298 ± 0.250 | 52044 ± 23721 | 38794 ± 12327 |
| 100 | 0.894 ± 0.105 | 28142 ± 2992 | 31854 ± 5644 |
| 8 | 1.244 ± 0.154 | 14388 ± 2056 | 11689 ± 2348 |
| 15 | 1.017 ± 0.164 | 22191 ± 2551 | 22294 ± 5080 |
| 114 | 0.756 ± 0.276 | 36560 ± 2288 | 51663 ± 13781 |
| 103 | 1.017 ± 0.156 | 7980 ± 982 | 7906 ± 1053 |
| 24 | 0.877 ± 0.202 | 8709 ± 571 | 10308 ± 2538 |
| 69 | 0.866 ± 0.159 | 2053 ± 619 | 2337 ± 376 |
| 112 | 0.863 ± 0.264 | 3223 ± 1053 | 3725 ± 242 |
| 37 | 0.203 ± 0.295 | 9684 ± 10456 | 88098 ± 42178 |
| 31 | 1.363 ± 0.258 | 8701 ± 3543 | 6817 ± 4061 |
| 44 | 1.089 ± 0.023 | 1644 ± 66 | 1509 ± 54 |
| 59 | 0.804 ± 0.269 | 912 ± 150 | 1194 ± 266 |
| 34 | 1.097 ± 0.208 | 34183 ± 5544 | 31917 ± 8430 |
| Initial cultures (1 mL) were grown overnight in LB medium containing 50 μg/ml kanamycin at 30 °C with 850 rpm agitation in 2 mL 96-deep well plates with gas permeable seals. Replicate cultures (200 μL; starting OD_600 nm_ ~ 0.1) were set up in the same medium in 96-well microtiter plates with a moisture barrier seal. Cultures were incubated at 30 °C with 300 rpm agitation in a microtiter plate reader. IPTG (0.1 mM) was added to the inducible cultures once OD_600 nm_ reached 0.55, and the incubation was continued overnight as before. Both the culture optical density and relative RFP fluorescence intensity (RFI) were monitored every 5 minutes (excitation and emission wavelengths of 584 and 607 nm, respectively). Results are expressed as the mean of the relative fluorescence units (RFU), which is fluorescence intensity per OD_600 nm_, with error bars representing one standard deviation of the data; ND: none detected; NA: not applicable. R^2^= 0.46 when comparing RFU data for 17, 3 and 13 to 34 with RFU data for the same promoters in *Halomonas* (Supplementary Table 5). | | | |

| Table 7 Comparative performance of selected constitutive promoters for CvFAP_G462V_RFP fusion expression in *Halomonas*. | | | | |
| --- | --- | --- | --- | --- |
| Clone/Promoter | Optical Density^a^  (OD_600 nm_) | Fluorescence Intensity^a^ (FI) | RFU  (FI/OD_600 nm_) | Propane  (mg/g cells) |
| pHal7 (empty vector) | 1.960 | ND | NA | NA |
| pBbA1a-RFP induced | 2.030 ± 0.044 | 2 ± 1 | 1 ± 1 | NA |
| pBbA1a-RFP uninduced | 2.153 ± 0.126 | 3 ± 1 | 2 ± 1 | NA |
| pBbA5a-RFP induced | 2.180 ± 0.121 | 4 ± 1 | 2 ± 1 | NA |
| pBbA5a-RFP uninduced | 2.010 ± 0.085 | 3 ± 1 | 2 ± 1 | NA |
| pHal2-RFP induced | 1.857 ± 0.045 | 299 ± 53 | 161 ± 28 | NA |
| pHal2-RFP uninduced | 2.027 ± 0.067 | 22 ± 1 | 11 ± 0 | NA |
| pHal7-RFP | 2.043 ± 0.180 | 18 ± 1 | 9 ± 1 | NA |
| pHal2-CvFAP_G462V_ induced | 2.170 | 3 | 1 | 1.70 ± 0.22 |
| pHal2-CvFAP_G462V_ uninduced | 2.220 | ND | ND | 0.06 ± 0.00 |
| pHal7-CvFAP_G462V_ | 1.710 | ND | ND | 0.26 ± 0.02 |
| pHal2-FAP_G462V_RFP induced | 2.600 | 73 | 28 | 1.44 ± 0.04 |
| pHal2-FAP_G462V_RFP uninduced | 2.090 | 10 | 5 | 0.17 ± 0.02 |
| pHal7-FAP_G462V_RFP | 2.150 | 35 | 16 | 0.76 ± 0.21 |
| 102 | 2.410 | 655 | 272 | 10.44 ± 0.51 |
| 8 | 2.09 | 348 | 166 | 7.20 ± 0.41 |
| 24 | 2.24 | 275 | 123 | 5.91 ± 1.03 |
| 103 | 2.630 | 338 | 128 | 5.15 ± 0.78 |
| 37 | 2.21 | 210 | 95 | 4.06 ± 0.48 |
| 31 | 2.08 | 160 | 77 | 3.88 ± 0.38 |
| 69 | 2.410 | 163 | 67 | 3.02 ± 0.21 |
| 99 | 2.230 | 63 | 28 | 1.45 ± 0.27 |
| 44 | 2.16 | 48 | 22 | 0.82 ± 0.05 |
| 59 | 2.190 | 33 | 15 | 0.55 ± 0.06 |
| Colonies of each construct were grown in YTN6 medium (5 mL) containing 50 μg/ml spectinomycin in 4 mL vials. The cultures were incubated overnight at 37 °C with 190 rpm agitation. Four aliquots of each culture (1 mL) were incubated in 4 mL vials in the presence of 10 mM butyric acid at 30 °C with 190 rpm. For inducible control clones, IPTG (0.1 mM) was added 2 hours prior the addition of butyric acid, and the incubation was continued overnight as before. At the end of the incubation, the culture growth (OD_600 nm_), RFP fluorescence (FI) and propane production (mg propane/L culture/OD_600 nm_) were determined. Results are expressed as RFU and propane production (mg propane/g cells wet weight), with error bars representing one standard deviation of the data. ^a^Data without SD values were from a single data point only, as the replicates were used for cultivation in vials for propane production. In this case, the culture OD 600 nm was taken at the point of propane analysis. ND: none detected; NA: not applicable. | | | | |

| Table 8 Comparative performance of selected constitutive promoters for CvFAP_G462V_RFP fusion expression in *E. coli*. | | | | |
| --- | --- | --- | --- | --- |
| Clone/Promoter | Optical Density^a^  (OD_600 nm_) | Fluorescence Intensity^a^ (FI) | RFU  (FI/OD_600 nm_) | Propane  (mg/g cells) |
| pHal7 (empty vector) | 4.748 | ND | NA | NA |
| pBbA1a-RFP induced | 7.243 ± 0.323 | 450353 ± 15437 | 62207 ± 1155 | NA |
| pBbA1a-RFP uninduced | 6.988 ± 0.087 | 415525 ± 3903 | 59469 ± 424 | NA |
| pBbA5a-RFP induced | 5.403 ± 0.077 | 130498 ± 7004 | 24170 ± 1622 | NA |
| pBbA5a-RFP uninduced | 5.473 ± 0.124 | 122025 ± 2109 | 22300 ± 131 | NA |
| pHal2-RFP induced | 5.290 ± 0.637 | 39213 ± 2073 | 7451 ± 473 | NA |
| pHal2-RFP uninduced | 5.153 ± 0.293 | 37820 ± 730 | 7351 ± 297 | NA |
| pHal7-RFP | 4.788 ± 0.125 | 179558 ± 1255 | 37520 ± 885 | NA |
| pHal2-CvFAP_G462V_ induced | 5.205 | ND | NA | 0.68 ± 0.02 |
| pHal2-CvFAP_G462V_ uninduced | 5.138 | ND | NA | 0.65 ± 0.03 |
| pHal7-CvFAP_G462V_ | 4.343 | ND | NA | 1.18 ± 0.00 |
| pHal2-FAP_G462V_RFP induced | 4.875 | 9148 | 1876 | 0.96 ± 0.03 |
| pHal2-FAP_G462V_RFP uninduced | 5.385 | 10363 | 1924 | 1.12 ± 0.12 |
| pHal7-FAP_G462V_RFP | 2.993 | 21500 | 7185 | 1.16 ± 0.54 |
| 102 | 4.688 | 40325 | 8603 | 6.13 ± 0.50 |
| 37 | 4.688 | 6200 | 1323 | 5.70 ± 0.57 |
| 103 | 4.703 | 43993 | 9355 | 5.64 ± 0.44 |
| 69 | 4.988 | 40288 | 8078 | 4.51 ± 0.24 |
| 59 | 3.233 | 15973 | 4941 | 2.37 ± 0.11 |
| 31 | 8.430 | 140 | 17 | 1.92 ± 1.86 |
| 8 | 2.865 | 18680 | 6520 | 0.35 ± 0.03 |
| 99 | 5.153 | 2735 | 531 | 0.07 ± 0.01 |
| 44 | 0.735 | 2735 | 3721 | 0.04 ± 0.01 |
| 24 | 4.740 | 33590 | 7086 | ND |
| Colonies of each construct were grown in LB medium (5 mL) containing 50 μg/ml kanamycin in 4 mL vials. The cultures were incubated overnight at 37 °C with 190 rpm agitation. Four aliquots of each culture (1 mL) were incubated in 4 mL vials in the presence of 10 mM butyric acid at 30 °C with 190 rpm. For inducible control clones, IPTG (0.1 mM) was added 2 hours prior the addition of butyric acid, and the incubation was continued overnight as before. At the end of the incubation, the culture growth (OD_600nm_), RFP fluorescence (FI) and propane production (mg propane/L culture/OD_600nm_) were determined. Results are expressed as RFU and propane production (mg propane/g cells wet weight), with error bars representing one standard deviation of the data. ^a^Fluorescence and optical density data without SD values were from a single culture only, as the replicates were used for cultivation in vials for propane production. In this case, the culture OD 600 nm was taken at the point of propane analysis. ND: none detected; NA: not applicable. | | | | |

**Supplementary Data**

Plasmid sequences

The following sequences are for plasmids used for data collection in this study. The website <https://www.addgene.org/browse/article/5445/> contains the sequences of the plasmids pBbA1a-RFP and pBbA5a-RFP (2). The remaining plasmids pHalT7P7-CvFAP_G462V_, pHalT7P7-RFP and pHal7-RFP-Stag were intermediate stages of the cloning of the constructs above, and no data was collected using them. Details of the oligonucleotides used to make these changes are found in Supplementary Information Table 2. For pHalV-RFP and pHalV-FAP_G462V_RFP library clones, the ‘V’ refers to the variable promoter sequence. To generate the exact sequences for each library member, the ***NNNNNNNNNNNNN*** should be substituted for the individual sequences in Supplementary Information Table 3. The genes are highlighted with grey shading, and the promoter regions are in blue. The start and stop codons are highlighted in bold.

>pHal7 GenBank:MW076822

TAGTGTAGCCGTAGTTAGGCCACCACTTCAAGAACTCTGTAGCACCGCCTACATACCTCGCTCTGCTAATCCTGTTACCAGTGGCTGCTGCCAGTGGCGATAAGTCGTGTCTTACCGGGTTGGACTCAAGACGATAGTTACCGGATAAGGCGCAGCGGTCGGGCTGAACGGGGGGTTCGTGCACACAGCCCAGCTTGGAGCGAACGACCTACACCGAACTGAGATACCTACAGCGTGAGCTATGAGAAAGCGCCACGCTTCCCGAAGGGAGAAAGGCGGACAGGCATCCGGTAAGCGGCAGGGTCGGAACAGGAGAGCGCACGAGGGAGCTTCCAGGGGGAAACGCCTGGTATCTTTATAGTCCTGTCGGGTTTCGCCACCTCTGACTTGAGCGTCGATTTTTGTGATGCTCGTCAGGGGGGCGGAGCCTATGGAAAAACGCCAGCAACGCGGCCGTGAAAGGCAGGCCGGTCCGTGGTGGCCACGGCCTCTAGGCCAGATCCAGCGGCATCTGGGTTAGTCGAGCGCGGGCCGCTTCCCATGTCTCACCAGGGCGAGCCTGTTTCGCGATCTCAGCATCTGAAATCTTCCCGGCCTTGCGCTTCGCTGGGGCCTTACCCACCGCCTTGGCGGGCTTCTTCGGTCCAAAACTGAACAACAGATGTGTGACCTTGCGCCCGGTCTTTCGCTGCGCCCACTCCACCTGTAGCGGGCTGTGCTCGTTGATCTGCGTCACGGCTGGATCAAGCACTCGCAACTTGAAGTCCTTGATCGAGGGATACCGGCCTTCCAGTTGAAACCACTTTCGCAGCTGGTCAATTTCTATTTCGCGCTGGCCGATGCTGTCCCATTGCATGAGCAGCTCGTAAAGCCTGATCGCGTGGGTGCTGTCCATCTTGGCCACGTCAGCCAAGGCGTATTTGGTGAACTGTTTGGTGAGTTCCGTCAGGTACGGCAGCATGTCTTTGGTGAACCTGAGTTCTACACGGCCCTCACCCTCCCGGTAGATGATTGTTTGCACCCAGCCGGTAATCATCACACTCGGTCTTTTCCCCTTGCCATTGGGCTCTTGGGTTAACCGGACTTCCCGCCGTTTCAGGCGCAGGGCCGCTTCTTTGAGCTGGTTGTAGGAAGATTCGATAGGGACACCCGCCATCGTCGCTATGTCCTCCGCCGTCACTGAATACATCACTTCATCGGTGACAGGCTCGCTCCTCTTCACCTGGCTAATACAGGCCAGAACGATCCGCTGTTCCTGAACACTGAGGCGATACGCGGCCTCGACCAGGGCATTGCTTTTGTAAACCATTGGGGGTGAGGCCACGTTCGACATTCCTTGTGTATAAGGGGACACTGTATCTGCGTCCCACAATACAACAAATCCGTCCCTTTACAACAACAAATCCGTCCCTTCTTAACAACAAATCCGTCCCTTAATGGCAACAAATCCGTCCCTTTTTAAACTCTAGAGGCCACGGATTACGTGGCCTGTAGACGTCCTAAAAGGTTTAAAAGGGAAAAGGAAGAAAAGGGTGGAAACGCAAAAAACGCACCACTACGTGGCCCCGTTGGGGCCGCATTTGTGCCCCTGAAGGGGCGGGGGAGGCGTCTGGGCAATCCCCGTTTTACCAGTCCCCTATCGCCGCCTGAGAGGGCGCAGGAAGCGAGTAATCAGGGTATCGAGGCGGATTCACCCTTGGCGTCCAACCAGCGGCACCAGCGGCGCCTGAGAGGGGCGCGCCCAGCTGTCTAGGGCGGCGGATTTGTCCTACTCAGGAGAGCGTTCACCGACAAACAACAGATAAAACGAAAGGCCCAGTCTTTCGACTGAGCCTTTCGTTTTATTTGATGCCTTTAATTAAAGCGGATAACAATTTCACACAGGAGGTTTTTCTATTGCGTACAACCGATAAAGGTATAGAGTTTGAGACTTTACTAGAGAAAGAGGAGAAATACTAGCTCGAGTCTGGTAAAGAAACCGCTGCTGCGAAATTTGAACGCCAGCACATGGACTCGTCTACTAGCGCAGCTTAATTAACCTAGGCTGCTGCCACCGCTGAGCAATAAAGCCCATGCAAGCTTGCGGCCGCGTCGTGACTGGGAAAACCCTGGCGACTAGTCTTGGACTCCTGTTGATAGATCCAGTAATGACCTCAGAACTCCATCTGGATTTGTTCAGAACGCTCGGTTGCCGCCGGGCGTTTTTTATTGGTGAGAATCCAGACGTTGTGTCTCAAAATCTCTGATGTTACATTGCACAAGATAAAAATATATCATCATGAACAATAAAACTGTCTGCTTACATAAACAGTAATACAAGGGGTGTTATGAGCCATATTCAACGGGAAACGTCTTGCTCGAGGCCGCGATTAAATTCCAACATGGATGCTGATTTATATGGGTATAAATGGGCTCGCGATAATGTCGGGCAATCAGGTGCGACAATCTATCGATTGTATGGGAAGCCCGATGCGCCAGAGTTGTTTCTGAAACATGGCAAAGGTAGCGTTGCCAATGATGTTACAGATGAGATGGTCAGACTAAACTGGCTGACGGAATTTATGCCTCTTCCGACCATCAAGCATTTTATCCGTACTCCTGATGATGCATGGTTACTCACCACTGCGATCCCCGGGAAAACAGCATTCCAGGTATTAGAAGAATATCCTGATTCAGGTGAAAATATTGTTGATGCGCTGGCAGTGTTCCTGCGCCGGTTGCATTCGATTCCTGTTTGTAATTGTCCTTTTAACAGCGATCGCGTATTTCGTCTCGCTCAGGCGCAATCACGAATGAATAACGGTTTGGTTGATGCGAGTGATTTTGATGACGAGCGTAATGGCTGGCCTGTTGAACAAGTCTGGAAAGAAATGCATAAGCTTTTGCCATTCTCACCGGATTCAGTCGTCACTCATGGTGATTTCTCACTTGATAACCTTATTTTTGACGAGGGGAAATTAATAGGTTGTATTGATGTTGGACGAGTCGGAATCGCAGACCGATACCAGGATCTTGCCATCCTATGGAACTGCCTCGGTGAGTTTTCTCCTTCATTACAGAAACGGCTTTTTCAAAAATATGGTATTGATAATCCTGATATGAATAAATTGCAGTTTCATTTGATGCTCGATGAGTTTTTCTAATCAGAATTGGTTAATTGGTTGTAACACTGGCAGAGCATTACGCTGACTTGACGGGACGGCGGCTTTGTTGAATAAATCGAACTTTTGCTGAGTTGAAGGATCAGATCACGCATCTTCCCGACAACGCAGACCGTTCCGTGGCAAAGCAAAAGTTCAAAATCACCAACTGGTCCACCTACAACAAAGCTCTCATCAACCGTGGCTCCCTCACTTTCTGGCTGGATGATGGGGCGATTCAGGCCTGGTATGAGTCAGCAACACCTTCTTCACGAGGCAGACCTCAGCGCTATTCTGACCTTGCCATCACGACTGTGCTGGTCATTAAACGCGTATTCAGGCTGACCCTGCGCGCTGCGCAGGGCTTTATTGATTCCATTTTTACACTGATGAATGTTCCGTTGCGCTGCCCGGATTACAGCCGGATCCTCTAGAGTCGACCTGCAGGCATGCTGATCGGCACGTAAGAGGTTCCAACTTTCACCATAATGAAATAAGATCACTACCGGGCGTATTTTTTGAGTTATCGAGATTTTCAGGAGCTAAGGAAGCTAAAATGCGCTCACGCAACTGGTCCAGAACCTTGACCGAACGCAGCGGTGGTAACGGCGCAGTGGCGGTTTTCATGGCTTGTTATGACTGTTTTTTTGGGGTACAGTCTATGCCTCGGGCATCCAAGCAGCAAGCGCGTTACGCCGTGGGTCGATGTTTGATGTTATGGAGCAGCAACGATGTTACGCAGCAGGGCAGTCGCCCTAAAACAAAGTTAAACATCATGAGGGAAGCGGTGATCGCCGAAGTATCGACTCAACTATCAGAGGTAGTTGGCGTCATCGAGCGCCATCTCGAACCGACGTTGCTGGCCGTACATTTGTACGGCTCCGCAGTGGATGGCGGCCTGAAGCCACACAGTGATATTGATTTGCTGGTTACGGTGACCGTAAGGCTTGATGAAACAACGCGGCGAGCTTTGATCAACGACCTTTTGGAAACTTCGGCTTCCCCTGGAGAGAGCGAGATTCTCCGCGCTGTAGAAGTCACCATTGTTGTGCACGACGACATCATTCCGTGGCGTTATCCAGCTAAGCGCGAACTGCAATTTGGAGAATGGCAGCGCAATGACATTCTTGCAGGTATCTTCGAGCCAGCCACGATCGACATTGATCTGGCTATCTTGCTGACAAAAGCAAGAGAACATAGCGTTGCCTTGGTAGGTCCAGCGGCGGAGGAACTCTTTGATCCGGTTCCTGAACAGGATCTATTTGAGGCGCTAAATGAAACCTTAACGCTATGGAACTCGCCGCCCGACTGGGCTGGCGATGAGCGAAATGTAGTGCTTACGTTGTCCCGCATTTGGTACAGCGCAGTAACCGGCAAAATCGCGCCGAAGGATGTCGCTGCCGACTGGGCAATGGAGCGCCTGCCGGCCCAGTATCAGCCCGTCATACTTGAAGCTAGACAGGCTTATCTTGGACAAGAAGAAGATCGCTTGGCCTCGCGCGCAGATCAGTTGGAAGAATTTGTCCACTACGTGAAAGGCGAGATCACCAAGGTAGTCGGCAAATAAACTAGTAAATAATAAAAAAGCCGGATTAATAATCTGGCTTTTTATATTCTCTGCATAACCCTGCTTCGGGGTCATTATAGCGATTTTTTCGGTATATCCATCCTTTTTCGCACGATATACAGGATTTTGCCAAAGGGTTCGTGTAGACTTTCCTTGGTGTATCCAACGGCGTCAGCCGGGCAGGATAGGTGAAGTAGGCCCACCCGCGAGCGGGTGTTCCTTCTTCACTGTCCCTTATTCGCACCTGGCGGTGCTCAACGGGAATCCTGCTCTGCGAGGCTGGCCGTAGGCCGGCCGATAATCTCATGACCAAAATCCCTTAACGTGAGTTTTCGTTCCACTGAGCGTCAGACCCCGTAGAAAAGATCAAAGGATCTTCTTGAGATCCTTTTTTTCTGCGCGTAATCTGCTGCTTGCAAACAAAAAAACCACCGCTACCAGCGGTGGTTTGTTTGCCGGATCAAGAGCTACCAACTCTTTTTCCGAAGGTAACTGGCTTCAGCAGAGCGCAGATACCAAATACTGTTCTTC

>pHal2-RFP GenBank:MW076823

TCTTCCGACCATCAAGCATTTTATCCGTACTCCTGATGATGCATGGTTACTCACCACTGCGATCCCCGGGAAAACAGCATTCCAGGTATTAGAAGAATATCCTGATTCAGGTGAAAATATTGTTGATGCGCTGGCAGTGTTCCTGCGCCGGTTGCATTCGATTCCTGTTTGTAATTGTCCTTTTAACAGCGATCGCGTATTTCGTCTCGCTCAGGCGCAATCACGAATGAATAACGGTTTGGTTGATGCGAGTGATTTTGATGACGAGCGTAATGGCTGGCCTGTTGAACAAGTCTGGAAAGAAATGCATAAGCTTTTGCCATTCTCACCGGATTCAGTCGTCACTCATGGTGATTTCTCACTTGATAACCTTATTTTTGACGAGGGGAAATTAATAGGTTGTATTGATGTTGGACGAGTCGGAATCGCAGACCGATACCAGGATCTTGCCATCCTATGGAACTGCCTCGGTGAGTTTTCTCCTTCATTACAGAAACGGCTTTTTCAAAAATATGGTATTGATAATCCTGATATGAATAAATTGCAGTTTCATTTGATGCTCGATGAGTTTTTCTAATCAGAATTGGTTAATTGGTTGTAACACTGGCAGAGCATTACGCTGACTTGACGGGACGGCGGCTTTGTTGAATAAATCGAACTTTTGCTGAGTTGAAGGATCAGATCACGCATCTTCCCGACAACGCAGACCGTTCCGTGGCAAAGCAAAAGTTCAAAATCACCAACTGGTCCACCTACAACAAAGCTCTCATCAACCGTGGCTCCCTCACTTTCTGGCTGGATGATGGGGCGATTCAGGCCTGGTATGAGTCAGCAACACCTTCTTCACGAGGCAGACCTCAGCGCTATTCTGACCTTGCCATCACGACTGTGCTGGTCATTAAACGCGTATTCAGGCTGACCCTGCGCGCTGCGCAGGGCTTTATTGATTCCATTTTTACACTGATGAATGTTCCGTTGCGCTGCCCGGATTACAGCCGGATCCTCTAGAGTCGACCTGCAGGCATGCTGATCGGCACGTAAGAGGTTCCAACTTTCACCATAATGAAATAAGATCACTACCGGGCGTATTTTTTGAGTTATCGAGATTTTCAGGAGCTAAGGAAGCTAAAATGCGCTCACGCAACTGGTCCAGAACCTTGACCGAACGCAGCGGTGGTAACGGCGCAGTGGCGGTTTTCATGGCTTGTTATGACTGTTTTTTTGGGGTACAGTCTATGCCTCGGGCATCCAAGCAGCAAGCGCGTTACGCCGTGGGTCGATGTTTGATGTTATGGAGCAGCAACGATGTTACGCAGCAGGGCAGTCGCCCTAAAACAAAGTTAAACATCATGAGGGAAGCGGTGATCGCCGAAGTATCGACTCAACTATCAGAGGTAGTTGGCGTCATCGAGCGCCATCTCGAACCGACGTTGCTGGCCGTACATTTGTACGGCTCCGCAGTGGATGGCGGCCTGAAGCCACACAGTGATATTGATTTGCTGGTTACGGTGACCGTAAGGCTTGATGAAACAACGCGGCGAGCTTTGATCAACGACCTTTTGGAAACTTCGGCTTCCCCTGGAGAGAGCGAGATTCTCCGCGCTGTAGAAGTCACCATTGTTGTGCACGACGACATCATTCCGTGGCGTTATCCAGCTAAGCGCGAACTGCAATTTGGAGAATGGCAGCGCAATGACATTCTTGCAGGTATCTTCGAGCCAGCCACGATCGACATTGATCTGGCTATCTTGCTGACAAAAGCAAGAGAACATAGCGTTGCCTTGGTAGGTCCAGCGGCGGAGGAACTCTTTGATCCGGTTCCTGAACAGGATCTATTTGAGGCGCTAAATGAAACCTTAACGCTATGGAACTCGCCGCCCGACTGGGCTGGCGATGAGCGAAATGTAGTGCTTACGTTGTCCCGCATTTGGTACAGCGCAGTAACCGGCAAAATCGCGCCGAAGGATGTCGCTGCCGACTGGGCAATGGAGCGCCTGCCGGCCCAGTATCAGCCCGTCATACTTGAAGCTAGACAGGCTTATCTTGGACAAGAAGAAGATCGCTTGGCCTCGCGCGCAGATCAGTTGGAAGAATTTGTCCACTACGTGAAAGGCGAGATCACCAAGGTAGTCGGCAAATAAACTAGTAAATAATAAAAAAGCCGGATTAATAATCTGGCTTTTTATATTCTCTGCATAACCCTGCTTCGGGGTCATTATAGCGATTTTTTCGGTATATCCATCCTTTTTCGCACGATATACAGGATTTTGCCAAAGGGTTCGTGTAGACTTTCCTTGGTGTATCCAACGGCGTCAGCCGGGCAGGATAGGTGAAGTAGGCCCACCCGCGAGCGGGTGTTCCTTCTTCACTGTCCCTTATTCGCACCTGGCGGTGCTCAACGGGAATCCTGCTCTGCGAGGCTGGCCGTAGGCCGGCCGATAATCTCATGACCAAAATCCCTTAACGTGAGTTTTCGTTCCACTGAGCGTCAGACCCCGTAGAAAAGATCAAAGGATCTTCTTGAGATCCTTTTTTTCTGCGCGTAATCTGCTGCTTGCAAACAAAAAAACCACCGCTACCAGCGGTGGTTTGTTTGCCGGATCAAGAGCTACCAACTCTTTTTCCGAAGGTAACTGGCTTCAGCAGAGCGCAGATACCAAATACTGTTCTTCTAGTGTAGCCGTAGTTAGGCCACCACTTCAAGAACTCTGTAGCACCGCCTACATACCTCGCTCTGCTAATCCTGTTACCAGTGGCTGCTGCCAGTGGCGATAAGTCGTGTCTTACCGGGTTGGACTCAAGACGATAGTTACCGGATAAGGCGCAGCGGTCGGGCTGAACGGGGGGTTCGTGCACACAGCCCAGCTTGGAGCGAACGACCTACACCGAACTGAGATACCTACAGCGTGAGCTATGAGAAAGCGCCACGCTTCCCGAAGGGAGAAAGGCGGACAGGCATCCGGTAAGCGGCAGGGTCGGAACAGGAGAGCGCACGAGGGAGCTTCCAGGGGGAAACGCCTGGTATCTTTATAGTCCTGTCGGGTTTCGCCACCTCTGACTTGAGCGTCGATTTTTGTGATGCTCGTCAGGGGGGCGGAGCCTATGGAAAAACGCCAGCAACGCGGCCGTGAAAGGCAGGCCGGTCCGTGGTGGCCACGGCCTCTAGGCCAGATCCAGCGGCATCTGGGTTAGTCGAGCGCGGGCCGCTTCCCATGTCTCACCAGGGCGAGCCTGTTTCGCGATCTCAGCATCTGAAATCTTCCCGGCCTTGCGCTTCGCTGGGGCCTTACCCACCGCCTTGGCGGGCTTCTTCGGTCCAAAACTGAACAACAGATGTGTGACCTTGCGCCCGGTCTTTCGCTGCGCCCACTCCACCTGTAGCGGGCTGTGCTCGTTGATCTGCGTCACGGCTGGATCAAGCACTCGCAACTTGAAGTCCTTGATCGAGGGATACCGGCCTTCCAGTTGAAACCACTTTCGCAGCTGGTCAATTTCTATTTCGCGCTGGCCGATGCTGTCCCATTGCATGAGCAGCTCGTAAAGCCTGATCGCGTGGGTGCTGTCCATCTTGGCCACGTCAGCCAAGGCGTATTTGGTGAACTGTTTGGTGAGTTCCGTCAGGTACGGCAGCATGTCTTTGGTGAACCTGAGTTCTACACGGCCCTCACCCTCCCGGTAGATGATTGTTTGCACCCAGCCGGTAATCATCACACTCGGTCTTTTCCCCTTGCCATTGGGCTCTTGGGTTAACCGGACTTCCCGCCGTTTCAGGCGCAGGGCCGCTTCTTTGAGCTGGTTGTAGGAAGATTCGATAGGGACACCCGCCATCGTCGCTATGTCCTCCGCCGTCACTGAATACATCACTTCATCGGTGACAGGCTCGCTCCTCTTCACCTGGCTAATACAGGCCAGAACGATCCGCTGTTCCTGAACACTGAGGCGATACGCGGCCTCGACCAGGGCATTGCTTTTGTAAACCATTGGGGGTGAGGCCACGTTCGACATTCCTTGTGTATAAGGGGACACTGTATCTGCGTCCCACAATACAACAAATCCGTCCCTTTACAACAACAAATCCGTCCCTTCTTAACAACAAATCCGTCCCTTAATGGCAACAAATCCGTCCCTTTTTAAACTCTAGAGGCCACGGATTACGTGGCCTGTAGACGTCCTAAAAGGTTTAAAAGGGAAAAGGAAGAAAAGGGTGGAAACGCAAAAAACGCACCACTACGTGGCCCCGTTGGGGCCGCATTTGTGCCCCTGAAGGGGCGGGGGAGGCGTCTGGGCAATCCCCGTTTTACCAGTCCCCTATCGCCGCCTGAGAGGGCGCAGGAAGCGAGTAATCAGGGTATCGAGGCGGATTCACCCTTGGCGTCCAACCAGCGGCACCAGCGGCGCCTGAGAGGGGCGCGCCCAGCTGTCTAGGGCGGCGGATTTGTCCTACTCAGGAGAGCGTTCACCGACAAACAACAGATAAAACGAAAGGCCCAGTCTTTCGACTGAGCCTTTCGTTTTATTTGATGCCTTTAATTAAAGCGGATAACAATTTCACACAGGAGGCCGCCTAGGCAAGGATGCCTCCACACCGCTCGTCACATCCTGCCCATGAGTTAATTATATTTGTGGCATTATAGGGAATTGTGAGCGCTCACAATTAGCTGTCACCGGATGTGCTTTCCGGTCTGATGAGTCCGTGAGGACGAAACAGCCTCTACAAATAATTTTGTTTAATACTAGAGAAAGAGGAGAAATACTAGTTTGTTTAACTTTAAGAAGGAGATATACC**ATG**GCGAGTAGCGAAGACGTTATCAAAGAGTTCATGCGTTTCAAAGTTCGTATGGAAGGTTCCGTTAACGGTCACGAGTTCGAAATCGAAGGTGAAGGTGAAGGTCGTCCGTACGAAGGTACCCAGACCGCTAAACTGAAAGTTACCAAAGGTGGTCCGCTGCCGTTCGCTTGGGACATCCTGTCCCCGCAGTTCCAGTACGGTTCCAAAGCTTACGTTAAACACCCGGCTGACATCCCGGACTACCTGAAACTGTCCTTCCCGGAAGGTTTCAAATGGGAACGTGTTATGAACTTCGAAGACGGTGGTGTTGTTACCGTTACCCAGGACTCCTCCCTGCAAGACGGTGAGTTCATCTACAAAGTTAAACTGCGTGGTACCAACTTCCCGTCCGACGGTCCGGTTATGCAGAAAAAAACCATGGGTTGGGAAGCTTCCACCGAACGTATGTACCCGGAAGACGGTGCTCTGAAAGGTGAAATCAAAATGCGTCTGAAACTGAAAGACGGTGGTCACTACGACGCTGAAGTTAAAACCACCTACATGGCTAAAAAACCGGTTCAGCTGCCGGGTGCTTACAAAACCGACATCAAACTGGACATCACCTCCCACAACGAAGACTACACCATCGTTGAACAGTACGAACGTGCTGAAGGTCGTCACTCCACCGGTGCT**TAA**CTCGAGTCTGGTAAAGAAACCGCTGCTGCGAAATTTGAACGCCAGCACATGGACTCGTCTACTAGCGCAGCTTAATTAACCTAGGCTGCTGCCACCGCTGAGCAATAAAGCCCATGCAAGCTTGCGGCCGCGTCGTGACTGGGAAAACCCTGGCGACTAGTCTTGGACTCCTGTTGATAGATCCAGTAATGACCTCAGAACTCCATCTGGATTTGTTCAGAACGCTCGGTTGCCGCCGGGCGTTTTTTATTGGTGAGAATCCAGACGTTGTGTCTCAAAATCTCTGATGTTACATTGCACAAGATAAAAATATATCATCATGAACAATAAAACTGTCTGCTTACATAAACAGTAATACAAGGGGTGTTATGAGCCATATTCAACGGGAAACGTCTTGCTCGAGGCCGCGATTAAATTCCAACATGGATGCTGATTTATATGGGTATAAATGGGCTCGCGATAATGTCGGGCAATCAGGTGCGACAATCTATCGATTGTATGGGAAGCCCGATGCGCCAGAGTTGTTTCTGAAACATGGCAAAGGTAGCGTTGCCAATGATGTTACAGATGAGATGGTCAGACTAAACTGGCTGACGGAATTTATGCC

>pHal7-RFP GenBank:MW076824

TAGTGTAGCCGTAGTTAGGCCACCACTTCAAGAACTCTGTAGCACCGCCTACATACCTCGCTCTGCTAATCCTGTTACCAGTGGCTGCTGCCAGTGGCGATAAGTCGTGTCTTACCGGGTTGGACTCAAGACGATAGTTACCGGATAAGGCGCAGCGGTCGGGCTGAACGGGGGGTTCGTGCACACAGCCCAGCTTGGAGCGAACGACCTACACCGAACTGAGATACCTACAGCGTGAGCTATGAGAAAGCGCCACGCTTCCCGAAGGGAGAAAGGCGGACAGGCATCCGGTAAGCGGCAGGGTCGGAACAGGAGAGCGCACGAGGGAGCTTCCAGGGGGAAACGCCTGGTATCTTTATAGTCCTGTCGGGTTTCGCCACCTCTGACTTGAGCGTCGATTTTTGTGATGCTCGTCAGGGGGGCGGAGCCTATGGAAAAACGCCAGCAACGCGGCCGTGAAAGGCAGGCCGGTCCGTGGTGGCCACGGCCTCTAGGCCAGATCCAGCGGCATCTGGGTTAGTCGAGCGCGGGCCGCTTCCCATGTCTCACCAGGGCGAGCCTGTTTCGCGATCTCAGCATCTGAAATCTTCCCGGCCTTGCGCTTCGCTGGGGCCTTACCCACCGCCTTGGCGGGCTTCTTCGGTCCAAAACTGAACAACAGATGTGTGACCTTGCGCCCGGTCTTTCGCTGCGCCCACTCCACCTGTAGCGGGCTGTGCTCGTTGATCTGCGTCACGGCTGGATCAAGCACTCGCAACTTGAAGTCCTTGATCGAGGGATACCGGCCTTCCAGTTGAAACCACTTTCGCAGCTGGTCAATTTCTATTTCGCGCTGGCCGATGCTGTCCCATTGCATGAGCAGCTCGTAAAGCCTGATCGCGTGGGTGCTGTCCATCTTGGCCACGTCAGCCAAGGCGTATTTGGTGAACTGTTTGGTGAGTTCCGTCAGGTACGGCAGCATGTCTTTGGTGAACCTGAGTTCTACACGGCCCTCACCCTCCCGGTAGATGATTGTTTGCACCCAGCCGGTAATCATCACACTCGGTCTTTTCCCCTTGCCATTGGGCTCTTGGGTTAACCGGACTTCCCGCCGTTTCAGGCGCAGGGCCGCTTCTTTGAGCTGGTTGTAGGAAGATTCGATAGGGACACCCGCCATCGTCGCTATGTCCTCCGCCGTCACTGAATACATCACTTCATCGGTGACAGGCTCGCTCCTCTTCACCTGGCTAATACAGGCCAGAACGATCCGCTGTTCCTGAACACTGAGGCGATACGCGGCCTCGACCAGGGCATTGCTTTTGTAAACCATTGGGGGTGAGGCCACGTTCGACATTCCTTGTGTATAAGGGGACACTGTATCTGCGTCCCACAATACAACAAATCCGTCCCTTTACAACAACAAATCCGTCCCTTCTTAACAACAAATCCGTCCCTTAATGGCAACAAATCCGTCCCTTTTTAAACTCTAGAGGCCACGGATTACGTGGCCTGTAGACGTCCTAAAAGGTTTAAAAGGGAAAAGGAAGAAAAGGGTGGAAACGCAAAAAACGCACCACTACGTGGCCCCGTTGGGGCCGCATTTGTGCCCCTGAAGGGGCGGGGGAGGCGTCTGGGCAATCCCCGTTTTACCAGTCCCCTATCGCCGCCTGAGAGGGCGCAGGAAGCGAGTAATCAGGGTATCGAGGCGGATTCACCCTTGGCGTCCAACCAGCGGCACCAGCGGCGCCTGAGAGGGGCGCGCCCAGCTGTCTAGGGCGGCGGATTTGTCCTACTCAGGAGAGCGTTCACCGACAAACAACAGATAAAACGAAAGGCCCAGTCTTTCGACTGAGCCTTTCGTTTTATTTGATGCCTTTAATTAAAGCGGATAACAATTTCACACAGGAGGTTTTTCTATTGCGTACAACCGATAAAGGTATAGAGTTTGAGACTTTACTAGAGAAAGAGGAGAAATACTAG**ATG**GCGAGTAGCGAAGACGTTATCAAAGAGTTCATGCGTTTCAAAGTTCGTATGGAAGGTTCCGTTAACGGTCACGAGTTCGAAATCGAAGGTGAAGGTGAAGGTCGTCCGTACGAAGGTACCCAGACCGCTAAACTGAAAGTTACCAAAGGTGGTCCGCTGCCGTTCGCTTGGGACATCCTGTCCCCGCAGTTCCAGTACGGTTCCAAAGCTTACGTTAAACACCCGGCTGACATCCCGGACTACCTGAAACTGTCCTTCCCGGAAGGTTTCAAATGGGAACGTGTTATGAACTTCGAAGACGGTGGTGTTGTTACCGTTACCCAGGACTCCTCCCTGCAAGACGGTGAGTTCATCTACAAAGTTAAACTGCGTGGTACCAACTTCCCGTCCGACGGTCCGGTTATGCAGAAAAAAACCATGGGTTGGGAAGCTTCCACCGAACGTATGTACCCGGAAGACGGTGCTCTGAAAGGTGAAATCAAAATGCGTCTGAAACTGAAAGACGGTGGTCACTACGACGCTGAAGTTAAAACCACCTACATGGCTAAAAAACCGGTTCAGCTGCCGGGTGCTTACAAAACCGACATCAAACTGGACATCACCTCCCACAACGAAGACTACACCATCGTTGAACAGTACGAACGTGCTGAAGGTCGTCACTCCACCGGTGCT**TAA**TTAACCTAGGCTGCTGCCACCGCTGAGCAATAAAGCCCATGCAAGCTTGCGGCCGCGTCGTGACTGGGAAAACCCTGGCGACTAGTCTTGGACTCCTGTTGATAGATCCAGTAATGACCTCAGAACTCCATCTGGATTTGTTCAGAACGCTCGGTTGCCGCCGGGCGTTTTTTATTGGTGAGAATCCAGACGTTGTGTCTCAAAATCTCTGATGTTACATTGCACAAGATAAAAATATATCATCATGAACAATAAAACTGTCTGCTTACATAAACAGTAATACAAGGGGTGTTATGAGCCATATTCAACGGGAAACGTCTTGCTCGAGGCCGCGATTAAATTCCAACATGGATGCTGATTTATATGGGTATAAATGGGCTCGCGATAATGTCGGGCAATCAGGTGCGACAATCTATCGATTGTATGGGAAGCCCGATGCGCCAGAGTTGTTTCTGAAACATGGCAAAGGTAGCGTTGCCAATGATGTTACAGATGAGATGGTCAGACTAAACTGGCTGACGGAATTTATGCCTCTTCCGACCATCAAGCATTTTATCCGTACTCCTGATGATGCATGGTTACTCACCACTGCGATCCCCGGGAAAACAGCATTCCAGGTATTAGAAGAATATCCTGATTCAGGTGAAAATATTGTTGATGCGCTGGCAGTGTTCCTGCGCCGGTTGCATTCGATTCCTGTTTGTAATTGTCCTTTTAACAGCGATCGCGTATTTCGTCTCGCTCAGGCGCAATCACGAATGAATAACGGTTTGGTTGATGCGAGTGATTTTGATGACGAGCGTAATGGCTGGCCTGTTGAACAAGTCTGGAAAGAAATGCATAAGCTTTTGCCATTCTCACCGGATTCAGTCGTCACTCATGGTGATTTCTCACTTGATAACCTTATTTTTGACGAGGGGAAATTAATAGGTTGTATTGATGTTGGACGAGTCGGAATCGCAGACCGATACCAGGATCTTGCCATCCTATGGAACTGCCTCGGTGAGTTTTCTCCTTCATTACAGAAACGGCTTTTTCAAAAATATGGTATTGATAATCCTGATATGAATAAATTGCAGTTTCATTTGATGCTCGATGAGTTTTTCTAATCAGAATTGGTTAATTGGTTGTAACACTGGCAGAGCATTACGCTGACTTGACGGGACGGCGGCTTTGTTGAATAAATCGAACTTTTGCTGAGTTGAAGGATCAGATCACGCATCTTCCCGACAACGCAGACCGTTCCGTGGCAAAGCAAAAGTTCAAAATCACCAACTGGTCCACCTACAACAAAGCTCTCATCAACCGTGGCTCCCTCACTTTCTGGCTGGATGATGGGGCGATTCAGGCCTGGTATGAGTCAGCAACACCTTCTTCACGAGGCAGACCTCAGCGCTATTCTGACCTTGCCATCACGACTGTGCTGGTCATTAAACGCGTATTCAGGCTGACCCTGCGCGCTGCGCAGGGCTTTATTGATTCCATTTTTACACTGATGAATGTTCCGTTGCGCTGCCCGGATTACAGCCGGATCCTCTAGAGTCGACCTGCAGGCATGCTGATCGGCACGTAAGAGGTTCCAACTTTCACCATAATGAAATAAGATCACTACCGGGCGTATTTTTTGAGTTATCGAGATTTTCAGGAGCTAAGGAAGCTAAAATGCGCTCACGCAACTGGTCCAGAACCTTGACCGAACGCAGCGGTGGTAACGGCGCAGTGGCGGTTTTCATGGCTTGTTATGACTGTTTTTTTGGGGTACAGTCTATGCCTCGGGCATCCAAGCAGCAAGCGCGTTACGCCGTGGGTCGATGTTTGATGTTATGGAGCAGCAACGATGTTACGCAGCAGGGCAGTCGCCCTAAAACAAAGTTAAACATCATGAGGGAAGCGGTGATCGCCGAAGTATCGACTCAACTATCAGAGGTAGTTGGCGTCATCGAGCGCCATCTCGAACCGACGTTGCTGGCCGTACATTTGTACGGCTCCGCAGTGGATGGCGGCCTGAAGCCACACAGTGATATTGATTTGCTGGTTACGGTGACCGTAAGGCTTGATGAAACAACGCGGCGAGCTTTGATCAACGACCTTTTGGAAACTTCGGCTTCCCCTGGAGAGAGCGAGATTCTCCGCGCTGTAGAAGTCACCATTGTTGTGCACGACGACATCATTCCGTGGCGTTATCCAGCTAAGCGCGAACTGCAATTTGGAGAATGGCAGCGCAATGACATTCTTGCAGGTATCTTCGAGCCAGCCACGATCGACATTGATCTGGCTATCTTGCTGACAAAAGCAAGAGAACATAGCGTTGCCTTGGTAGGTCCAGCGGCGGAGGAACTCTTTGATCCGGTTCCTGAACAGGATCTATTTGAGGCGCTAAATGAAACCTTAACGCTATGGAACTCGCCGCCCGACTGGGCTGGCGATGAGCGAAATGTAGTGCTTACGTTGTCCCGCATTTGGTACAGCGCAGTAACCGGCAAAATCGCGCCGAAGGATGTCGCTGCCGACTGGGCAATGGAGCGCCTGCCGGCCCAGTATCAGCCCGTCATACTTGAAGCTAGACAGGCTTATCTTGGACAAGAAGAAGATCGCTTGGCCTCGCGCGCAGATCAGTTGGAAGAATTTGTCCACTACGTGAAAGGCGAGATCACCAAGGTAGTCGGCAAATAAACTAGTAAATAATAAAAAAGCCGGATTAATAATCTGGCTTTTTATATTCTCTGCATAACCCTGCTTCGGGGTCATTATAGCGATTTTTTCGGTATATCCATCCTTTTTCGCACGATATACAGGATTTTGCCAAAGGGTTCGTGTAGACTTTCCTTGGTGTATCCAACGGCGTCAGCCGGGCAGGATAGGTGAAGTAGGCCCACCCGCGAGCGGGTGTTCCTTCTTCACTGTCCCTTATTCGCACCTGGCGGTGCTCAACGGGAATCCTGCTCTGCGAGGCTGGCCGTAGGCCGGCCGATAATCTCATGACCAAAATCCCTTAACGTGAGTTTTCGTTCCACTGAGCGTCAGACCCCGTAGAAAAGATCAAAGGATCTTCTTGAGATCCTTTTTTTCTGCGCGTAATCTGCTGCTTGCAAACAAAAAAACCACCGCTACCAGCGGTGGTTTGTTTGCCGGATCAAGAGCTACCAACTCTTTTTCCGAAGGTAACTGGCTTCAGCAGAGCGCAGATACCAAATACTGTTCTTC

>pHalV-RFP GenBank:MW076825

TAGTGTAGCCGTAGTTAGGCCACCACTTCAAGAACTCTGTAGCACCGCCTACATACCTCGCTCTGCTAATCCTGTTACCAGTGGCTGCTGCCAGTGGCGATAAGTCGTGTCTTACCGGGTTGGACTCAAGACGATAGTTACCGGATAAGGCGCAGCGGTCGGGCTGAACGGGGGGTTCGTGCACACAGCCCAGCTTGGAGCGAACGACCTACACCGAACTGAGATACCTACAGCGTGAGCTATGAGAAAGCGCCACGCTTCCCGAAGGGAGAAAGGCGGACAGGCATCCGGTAAGCGGCAGGGTCGGAACAGGAGAGCGCACGAGGGAGCTTCCAGGGGGAAACGCCTGGTATCTTTATAGTCCTGTCGGGTTTCGCCACCTCTGACTTGAGCGTCGATTTTTGTGATGCTCGTCAGGGGGGCGGAGCCTATGGAAAAACGCCAGCAACGCGGCCGTGAAAGGCAGGCCGGTCCGTGGTGGCCACGGCCTCTAGGCCAGATCCAGCGGCATCTGGGTTAGTCGAGCGCGGGCCGCTTCCCATGTCTCACCAGGGCGAGCCTGTTTCGCGATCTCAGCATCTGAAATCTTCCCGGCCTTGCGCTTCGCTGGGGCCTTACCCACCGCCTTGGCGGGCTTCTTCGGTCCAAAACTGAACAACAGATGTGTGACCTTGCGCCCGGTCTTTCGCTGCGCCCACTCCACCTGTAGCGGGCTGTGCTCGTTGATCTGCGTCACGGCTGGATCAAGCACTCGCAACTTGAAGTCCTTGATCGAGGGATACCGGCCTTCCAGTTGAAACCACTTTCGCAGCTGGTCAATTTCTATTTCGCGCTGGCCGATGCTGTCCCATTGCATGAGCAGCTCGTAAAGCCTGATCGCGTGGGTGCTGTCCATCTTGGCCACGTCAGCCAAGGCGTATTTGGTGAACTGTTTGGTGAGTTCCGTCAGGTACGGCAGCATGTCTTTGGTGAACCTGAGTTCTACACGGCCCTCACCCTCCCGGTAGATGATTGTTTGCACCCAGCCGGTAATCATCACACTCGGTCTTTTCCCCTTGCCATTGGGCTCTTGGGTTAACCGGACTTCCCGCCGTTTCAGGCGCAGGGCCGCTTCTTTGAGCTGGTTGTAGGAAGATTCGATAGGGACACCCGCCATCGTCGCTATGTCCTCCGCCGTCACTGAATACATCACTTCATCGGTGACAGGCTCGCTCCTCTTCACCTGGCTAATACAGGCCAGAACGATCCGCTGTTCCTGAACACTGAGGCGATACGCGGCCTCGACCAGGGCATTGCTTTTGTAAACCATTGGGGGTGAGGCCACGTTCGACATTCCTTGTGTATAAGGGGACACTGTATCTGCGTCCCACAATACAACAAATCCGTCCCTTTACAACAACAAATCCGTCCCTTCTTAACAACAAATCCGTCCCTTAATGGCAACAAATCCGTCCCTTTTTAAACTCTAGAGGCCACGGATTACGTGGCCTGTAGACGTCCTAAAAGGTTTAAAAGGGAAAAGGAAGAAAAGGGTGGAAACGCAAAAAACGCACCACTACGTGGCCCCGTTGGGGCCGCATTTGTGCCCCTGAAGGGGCGGGGGAGGCGTCTGGGCAATCCCCGTTTTACCAGTCCCCTATCGCCGCCTGAGAGGGCGCAGGAAGCGAGTAATCAGGGTATCGAGGCGGATTCACCCTTGGCGTCCAACCAGCGGCACCAGCGGCGCCTGAGAGGGGCGCGCCCAGCTGTCTAGGGCGGCGGATTTGTCCTACTCAGGAGAGCGTTCACCGACAAACAACAGATAAAACGAAAGGCCCAGTCTTTCGACTGAGCCTTTCGTTTTATTTGATGCCTTTAATTAAAGCGGATAACAATTTCACACAGGAGGTTTTTCTATTGCGT***NNNNNNNNNNNNN***GTATAGAGTTTGAGACTTTACTAGAGAAAGAGGAGAAATACTAG**ATG**GCGAGTAGCGAAGACGTTATCAAAGAGTTCATGCGTTTCAAAGTTCGTATGGAAGGTTCCGTTAACGGTCACGAGTTCGAAATCGAAGGTGAAGGTGAAGGTCGTCCGTACGAAGGTACCCAGACCGCTAAACTGAAAGTTACCAAAGGTGGTCCGCTGCCGTTCGCTTGGGACATCCTGTCCCCGCAGTTCCAGTACGGTTCCAAAGCTTACGTTAAACACCCGGCTGACATCCCGGACTACCTGAAACTGTCCTTCCCGGAAGGTTTCAAATGGGAACGTGTTATGAACTTCGAAGACGGTGGTGTTGTTACCGTTACCCAGGACTCCTCCCTGCAAGACGGTGAGTTCATCTACAAAGTTAAACTGCGTGGTACCAACTTCCCGTCCGACGGTCCGGTTATGCAGAAAAAAACCATGGGTTGGGAAGCTTCCACCGAACGTATGTACCCGGAAGACGGTGCTCTGAAAGGTGAAATCAAAATGCGTCTGAAACTGAAAGACGGTGGTCACTACGACGCTGAAGTTAAAACCACCTACATGGCTAAAAAACCGGTTCAGCTGCCGGGTGCTTACAAAACCGACATCAAACTGGACATCACCTCCCACAACGAAGACTACACCATCGTTGAACAGTACGAACGTGCTGAAGGTCGTCACTCCACCGGTGCT**TAA**TTAACCTAGGCTGCTGCCACCGCTGAGCAATAAAGCCCATGCAAGCTTGCGGCCGCGTCGTGACTGGGAAAACCCTGGCGACTAGTCTTGGACTCCTGTTGATAGATCCAGTAATGACCTCAGAACTCCATCTGGATTTGTTCAGAACGCTCGGTTGCCGCCGGGCGTTTTTTATTGGTGAGAATCCAGACGTTGTGTCTCAAAATCTCTGATGTTACATTGCACAAGATAAAAATATATCATCATGAACAATAAAACTGTCTGCTTACATAAACAGTAATACAAGGGGTGTTATGAGCCATATTCAACGGGAAACGTCTTGCTCGAGGCCGCGATTAAATTCCAACATGGATGCTGATTTATATGGGTATAAATGGGCTCGCGATAATGTCGGGCAATCAGGTGCGACAATCTATCGATTGTATGGGAAGCCCGATGCGCCAGAGTTGTTTCTGAAACATGGCAAAGGTAGCGTTGCCAATGATGTTACAGATGAGATGGTCAGACTAAACTGGCTGACGGAATTTATGCCTCTTCCGACCATCAAGCATTTTATCCGTACTCCTGATGATGCATGGTTACTCACCACTGCGATCCCCGGGAAAACAGCATTCCAGGTATTAGAAGAATATCCTGATTCAGGTGAAAATATTGTTGATGCGCTGGCAGTGTTCCTGCGCCGGTTGCATTCGATTCCTGTTTGTAATTGTCCTTTTAACAGCGATCGCGTATTTCGTCTCGCTCAGGCGCAATCACGAATGAATAACGGTTTGGTTGATGCGAGTGATTTTGATGACGAGCGTAATGGCTGGCCTGTTGAACAAGTCTGGAAAGAAATGCATAAGCTTTTGCCATTCTCACCGGATTCAGTCGTCACTCATGGTGATTTCTCACTTGATAACCTTATTTTTGACGAGGGGAAATTAATAGGTTGTATTGATGTTGGACGAGTCGGAATCGCAGACCGATACCAGGATCTTGCCATCCTATGGAACTGCCTCGGTGAGTTTTCTCCTTCATTACAGAAACGGCTTTTTCAAAAATATGGTATTGATAATCCTGATATGAATAAATTGCAGTTTCATTTGATGCTCGATGAGTTTTTCTAATCAGAATTGGTTAATTGGTTGTAACACTGGCAGAGCATTACGCTGACTTGACGGGACGGCGGCTTTGTTGAATAAATCGAACTTTTGCTGAGTTGAAGGATCAGATCACGCATCTTCCCGACAACGCAGACCGTTCCGTGGCAAAGCAAAAGTTCAAAATCACCAACTGGTCCACCTACAACAAAGCTCTCATCAACCGTGGCTCCCTCACTTTCTGGCTGGATGATGGGGCGATTCAGGCCTGGTATGAGTCAGCAACACCTTCTTCACGAGGCAGACCTCAGCGCTATTCTGACCTTGCCATCACGACTGTGCTGGTCATTAAACGCGTATTCAGGCTGACCCTGCGCGCTGCGCAGGGCTTTATTGATTCCATTTTTACACTGATGAATGTTCCGTTGCGCTGCCCGGATTACAGCCGGATCCTCTAGAGTCGACCTGCAGGCATGCTGATCGGCACGTAAGAGGTTCCAACTTTCACCATAATGAAATAAGATCACTACCGGGCGTATTTTTTGAGTTATCGAGATTTTCAGGAGCTAAGGAAGCTAAAATGCGCTCACGCAACTGGTCCAGAACCTTGACCGAACGCAGCGGTGGTAACGGCGCAGTGGCGGTTTTCATGGCTTGTTATGACTGTTTTTTTGGGGTACAGTCTATGCCTCGGGCATCCAAGCAGCAAGCGCGTTACGCCGTGGGTCGATGTTTGATGTTATGGAGCAGCAACGATGTTACGCAGCAGGGCAGTCGCCCTAAAACAAAGTTAAACATCATGAGGGAAGCGGTGATCGCCGAAGTATCGACTCAACTATCAGAGGTAGTTGGCGTCATCGAGCGCCATCTCGAACCGACGTTGCTGGCCGTACATTTGTACGGCTCCGCAGTGGATGGCGGCCTGAAGCCACACAGTGATATTGATTTGCTGGTTACGGTGACCGTAAGGCTTGATGAAACAACGCGGCGAGCTTTGATCAACGACCTTTTGGAAACTTCGGCTTCCCCTGGAGAGAGCGAGATTCTCCGCGCTGTAGAAGTCACCATTGTTGTGCACGACGACATCATTCCGTGGCGTTATCCAGCTAAGCGCGAACTGCAATTTGGAGAATGGCAGCGCAATGACATTCTTGCAGGTATCTTCGAGCCAGCCACGATCGACATTGATCTGGCTATCTTGCTGACAAAAGCAAGAGAACATAGCGTTGCCTTGGTAGGTCCAGCGGCGGAGGAACTCTTTGATCCGGTTCCTGAACAGGATCTATTTGAGGCGCTAAATGAAACCTTAACGCTATGGAACTCGCCGCCCGACTGGGCTGGCGATGAGCGAAATGTAGTGCTTACGTTGTCCCGCATTTGGTACAGCGCAGTAACCGGCAAAATCGCGCCGAAGGATGTCGCTGCCGACTGGGCAATGGAGCGCCTGCCGGCCCAGTATCAGCCCGTCATACTTGAAGCTAGACAGGCTTATCTTGGACAAGAAGAAGATCGCTTGGCCTCGCGCGCAGATCAGTTGGAAGAATTTGTCCACTACGTGAAAGGCGAGATCACCAAGGTAGTCGGCAAATAAACTAGTAAATAATAAAAAAGCCGGATTAATAATCTGGCTTTTTATATTCTCTGCATAACCCTGCTTCGGGGTCATTATAGCGATTTTTTCGGTATATCCATCCTTTTTCGCACGATATACAGGATTTTGCCAAAGGGTTCGTGTAGACTTTCCTTGGTGTATCCAACGGCGTCAGCCGGGCAGGATAGGTGAAGTAGGCCCACCCGCGAGCGGGTGTTCCTTCTTCACTGTCCCTTATTCGCACCTGGCGGTGCTCAACGGGAATCCTGCTCTGCGAGGCTGGCCGTAGGCCGGCCGATAATCTCATGACCAAAATCCCTTAACGTGAGTTTTCGTTCCACTGAGCGTCAGACCCCGTAGAAAAGATCAAAGGATCTTCTTGAGATCCTTTTTTTCTGCGCGTAATCTGCTGCTTGCAAACAAAAAAACCACCGCTACCAGCGGTGGTTTGTTTGCCGGATCAAGAGCTACCAACTCTTTTTCCGAAGGTAACTGGCTTCAGCAGAGCGCAGATACCAAATACTGTTCTTC

>pHal2-CvFAP_G462V_ GenBank:MW076826

TCTTCCGACCATCAAGCATTTTATCCGTACTCCTGATGATGCATGGTTACTCACCACTGCGATCCCCGGGAAAACAGCATTCCAGGTATTAGAAGAATATCCTGATTCAGGTGAAAATATTGTTGATGCGCTGGCAGTGTTCCTGCGCCGGTTGCATTCGATTCCTGTTTGTAATTGTCCTTTTAACAGCGATCGCGTATTTCGTCTCGCTCAGGCGCAATCACGAATGAATAACGGTTTGGTTGATGCGAGTGATTTTGATGACGAGCGTAATGGCTGGCCTGTTGAACAAGTCTGGAAAGAAATGCATAAGCTTTTGCCATTCTCACCGGATTCAGTCGTCACTCATGGTGATTTCTCACTTGATAACCTTATTTTTGACGAGGGGAAATTAATAGGTTGTATTGATGTTGGACGAGTCGGAATCGCAGACCGATACCAGGATCTTGCCATCCTATGGAACTGCCTCGGTGAGTTTTCTCCTTCATTACAGAAACGGCTTTTTCAAAAATATGGTATTGATAATCCTGATATGAATAAATTGCAGTTTCATTTGATGCTCGATGAGTTTTTCTAATCAGAATTGGTTAATTGGTTGTAACACTGGCAGAGCATTACGCTGACTTGACGGGACGGCGGCTTTGTTGAATAAATCGAACTTTTGCTGAGTTGAAGGATCAGATCACGCATCTTCCCGACAACGCAGACCGTTCCGTGGCAAAGCAAAAGTTCAAAATCACCAACTGGTCCACCTACAACAAAGCTCTCATCAACCGTGGCTCCCTCACTTTCTGGCTGGATGATGGGGCGATTCAGGCCTGGTATGAGTCAGCAACACCTTCTTCACGAGGCAGACCTCAGCGCTATTCTGACCTTGCCATCACGACTGTGCTGGTCATTAAACGCGTATTCAGGCTGACCCTGCGCGCTGCGCAGGGCTTTATTGATTCCATTTTTACACTGATGAATGTTCCGTTGCGCTGCCCGGATTACAGCCGGATCCTCTAGAGTCGACCTGCAGGCATGCTGATCGGCACGTAAGAGGTTCCAACTTTCACCATAATGAAATAAGATCACTACCGGGCGTATTTTTTGAGTTATCGAGATTTTCAGGAGCTAAGGAAGCTAAAATGCGCTCACGCAACTGGTCCAGAACCTTGACCGAACGCAGCGGTGGTAACGGCGCAGTGGCGGTTTTCATGGCTTGTTATGACTGTTTTTTTGGGGTACAGTCTATGCCTCGGGCATCCAAGCAGCAAGCGCGTTACGCCGTGGGTCGATGTTTGATGTTATGGAGCAGCAACGATGTTACGCAGCAGGGCAGTCGCCCTAAAACAAAGTTAAACATCATGAGGGAAGCGGTGATCGCCGAAGTATCGACTCAACTATCAGAGGTAGTTGGCGTCATCGAGCGCCATCTCGAACCGACGTTGCTGGCCGTACATTTGTACGGCTCCGCAGTGGATGGCGGCCTGAAGCCACACAGTGATATTGATTTGCTGGTTACGGTGACCGTAAGGCTTGATGAAACAACGCGGCGAGCTTTGATCAACGACCTTTTGGAAACTTCGGCTTCCCCTGGAGAGAGCGAGATTCTCCGCGCTGTAGAAGTCACCATTGTTGTGCACGACGACATCATTCCGTGGCGTTATCCAGCTAAGCGCGAACTGCAATTTGGAGAATGGCAGCGCAATGACATTCTTGCAGGTATCTTCGAGCCAGCCACGATCGACATTGATCTGGCTATCTTGCTGACAAAAGCAAGAGAACATAGCGTTGCCTTGGTAGGTCCAGCGGCGGAGGAACTCTTTGATCCGGTTCCTGAACAGGATCTATTTGAGGCGCTAAATGAAACCTTAACGCTATGGAACTCGCCGCCCGACTGGGCTGGCGATGAGCGAAATGTAGTGCTTACGTTGTCCCGCATTTGGTACAGCGCAGTAACCGGCAAAATCGCGCCGAAGGATGTCGCTGCCGACTGGGCAATGGAGCGCCTGCCGGCCCAGTATCAGCCCGTCATACTTGAAGCTAGACAGGCTTATCTTGGACAAGAAGAAGATCGCTTGGCCTCGCGCGCAGATCAGTTGGAAGAATTTGTCCACTACGTGAAAGGCGAGATCACCAAGGTAGTCGGCAAATAAACTAGTAAATAATAAAAAAGCCGGATTAATAATCTGGCTTTTTATATTCTCTGCATAACCCTGCTTCGGGGTCATTATAGCGATTTTTTCGGTATATCCATCCTTTTTCGCACGATATACAGGATTTTGCCAAAGGGTTCGTGTAGACTTTCCTTGGTGTATCCAACGGCGTCAGCCGGGCAGGATAGGTGAAGTAGGCCCACCCGCGAGCGGGTGTTCCTTCTTCACTGTCCCTTATTCGCACCTGGCGGTGCTCAACGGGAATCCTGCTCTGCGAGGCTGGCCGTAGGCCGGCCGATAATCTCATGACCAAAATCCCTTAACGTGAGTTTTCGTTCCACTGAGCGTCAGACCCCGTAGAAAAGATCAAAGGATCTTCTTGAGATCCTTTTTTTCTGCGCGTAATCTGCTGCTTGCAAACAAAAAAACCACCGCTACCAGCGGTGGTTTGTTTGCCGGATCAAGAGCTACCAACTCTTTTTCCGAAGGTAACTGGCTTCAGCAGAGCGCAGATACCAAATACTGTTCTTCTAGTGTAGCCGTAGTTAGGCCACCACTTCAAGAACTCTGTAGCACCGCCTACATACCTCGCTCTGCTAATCCTGTTACCAGTGGCTGCTGCCAGTGGCGATAAGTCGTGTCTTACCGGGTTGGACTCAAGACGATAGTTACCGGATAAGGCGCAGCGGTCGGGCTGAACGGGGGGTTCGTGCACACAGCCCAGCTTGGAGCGAACGACCTACACCGAACTGAGATACCTACAGCGTGAGCTATGAGAAAGCGCCACGCTTCCCGAAGGGAGAAAGGCGGACAGGCATCCGGTAAGCGGCAGGGTCGGAACAGGAGAGCGCACGAGGGAGCTTCCAGGGGGAAACGCCTGGTATCTTTATAGTCCTGTCGGGTTTCGCCACCTCTGACTTGAGCGTCGATTTTTGTGATGCTCGTCAGGGGGGCGGAGCCTATGGAAAAACGCCAGCAACGCGGCCGTGAAAGGCAGGCCGGTCCGTGGTGGCCACGGCCTCTAGGCCAGATCCAGCGGCATCTGGGTTAGTCGAGCGCGGGCCGCTTCCCATGTCTCACCAGGGCGAGCCTGTTTCGCGATCTCAGCATCTGAAATCTTCCCGGCCTTGCGCTTCGCTGGGGCCTTACCCACCGCCTTGGCGGGCTTCTTCGGTCCAAAACTGAACAACAGATGTGTGACCTTGCGCCCGGTCTTTCGCTGCGCCCACTCCACCTGTAGCGGGCTGTGCTCGTTGATCTGCGTCACGGCTGGATCAAGCACTCGCAACTTGAAGTCCTTGATCGAGGGATACCGGCCTTCCAGTTGAAACCACTTTCGCAGCTGGTCAATTTCTATTTCGCGCTGGCCGATGCTGTCCCATTGCATGAGCAGCTCGTAAAGCCTGATCGCGTGGGTGCTGTCCATCTTGGCCACGTCAGCCAAGGCGTATTTGGTGAACTGTTTGGTGAGTTCCGTCAGGTACGGCAGCATGTCTTTGGTGAACCTGAGTTCTACACGGCCCTCACCCTCCCGGTAGATGATTGTTTGCACCCAGCCGGTAATCATCACACTCGGTCTTTTCCCCTTGCCATTGGGCTCTTGGGTTAACCGGACTTCCCGCCGTTTCAGGCGCAGGGCCGCTTCTTTGAGCTGGTTGTAGGAAGATTCGATAGGGACACCCGCCATCGTCGCTATGTCCTCCGCCGTCACTGAATACATCACTTCATCGGTGACAGGCTCGCTCCTCTTCACCTGGCTAATACAGGCCAGAACGATCCGCTGTTCCTGAACACTGAGGCGATACGCGGCCTCGACCAGGGCATTGCTTTTGTAAACCATTGGGGGTGAGGCCACGTTCGACATTCCTTGTGTATAAGGGGACACTGTATCTGCGTCCCACAATACAACAAATCCGTCCCTTTACAACAACAAATCCGTCCCTTCTTAACAACAAATCCGTCCCTTAATGGCAACAAATCCGTCCCTTTTTAAACTCTAGAGGCCACGGATTACGTGGCCTGTAGACGTCCTAAAAGGTTTAAAAGGGAAAAGGAAGAAAAGGGTGGAAACGCAAAAAACGCACCACTACGTGGCCCCGTTGGGGCCGCATTTGTGCCCCTGAAGGGGCGGGGGAGGCGTCTGGGCAATCCCCGTTTTACCAGTCCCCTATCGCCGCCTGAGAGGGCGCAGGAAGCGAGTAATCAGGGTATCGAGGCGGATTCACCCTTGGCGTCCAACCAGCGGCACCAGCGGCGCCTGAGAGGGGCGCGCCCAGCTGTCTAGGGCGGCGGATTTGTCCTACTCAGGAGAGCGTTCACCGACAAACAACAGATAAAACGAAAGGCCCAGTCTTTCGACTGAGCCTTTCGTTTTATTTGATGCCTTTAATTAAAGCGGATAACAATTTCACACAGGAGGCCGCCTAGGCAAGGATGCCTCCACACCGCTCGTCACATCCTGCCCATGAGTTAATTATATTTGTGGCATTATAGGGAATTGTGAGCGCTCACAATTAGCTGTCACCGGATGTGCTTTCCGGTCTGATGAGTCCGTGAGGACGAAACAGCCTCTACAAATAATTTTGTTTAATACTAGAGAAAGAGGAGAAATACTAGTTTGTTTAACTTTAAGAAGGAGATATACC**ATG**GCCAGCGCAGTTGAAGATATTCGTAAAGTTCTGAGCGATAGCAGCAGTCCGGTTGCAGGTCAGAAATATGATTATATTCTGGTTGGTGGTGGCACCGCAGCATGTGTTCTGGCAAATCGTCTGAGCGCAGATGGTAGCAAACGTGTTCTGGTTCTGGAAGCAGGTCCGGATAATACCAGCCGTGATGTTAAAATTCCGGCAGCAATTACCCGTCTGTTTCGTAGTCCGCTGGATTGGAACCTGTTTAGCGAACTGCAAGAACAGCTGGCAGAACGTCAGATTTATATGGCACGTGGTCGTCTGTTAGGTGGTAGCAGCGCAACCAATGCAACCCTGTATCATCGTGGTGCAGCCGGTGATTATGATGCATGGGGTGTTGAAGGTTGGAGCAGCGAAGATGTTCTGAGCTGGTTTGTTCAGGCAGAAACCAATGCCGATTTTGGTCCGGGTGCATATCATGGTAGCGGTGGTCCGATGCGTGTTGAAAATCCGCGTTATACCAATAAACAGCTGCATACCGCATTTTTCAAAGCAGCAGAAGAAGTTGGTCTGACCCCGAATAGCGATTTTAATGATTGGAGCCATGATCACGCAGGTTATGGCACCTTTCAGGTTATGCAGGATAAAGGCACCCGTGCAGATATGTATCGTCAGTATCTGAAACCGGTTCTGGGTCGTCGTAATCTGCAGGTTCTGACCGGTGCAGCAGTTACCAAAGTTAATATTGATCAGGCAGCAGGTAAAGCACAGGCACTGGGCGTTGAATTTTCAACCGATGGTCCGACCGGTGAACGTCTGTCAGCAGAACTGGCACCTGGTGGTGAAGTTATTATGTGTGCCGGTGCAGTTCATACCCCGTTTCTGCTGAAACATAGCGGTGTTGGTCCGAGCGCAGAACTGAAAGAATTTGGTATTCCGGTTGTTAGCAATCTGGCAGGCGTTGGCCAGAATCTGCAAGATCAGCCTGCATGTCTGACCGCAGCACCGGTTAAAGAAAAATATGATGGTATTGCCATCAGCGACCACATCTATAATGAAAAAGGTCAGATTCGCAAACGTGCCATTGCAAGCTATCTGCTTGGTGGTCGTGGTGGCCTGACCAGTACCGGTTGTGATCGCGGTGCATTTGTTCGTACCGCAGGTCAGGCACTGCCGGATCTGCAAGTTCGTTTTGTTCCTGGTATGGCACTGGATCCGGATGTTGTTAGCACCTATGTGCGTTTTGCCAAATTTCAGAGCCAGGGCCTGAAATGGCCGAGCGGTATTACCATGCAGCTGATTGCATGTCGTCCGCAGAGTACCGGTAGCGTGGGTCTGAAAAGCGCAGATCCGTTTGCACCGCCTAAACTGAGTCCGGGTTATCTGACCGATAAAGATGGTGCAGATCTGGCGACCCTGCGTAAAGGTATTCATTGGGCACGTGATGTTGCACGTAGCTCAGCACTGAGCGAATATCTGGATGGTGAACTGTTTCCTGGTAGTGGTGTTGTTAGTGATGATCAGATCGATGAATATATCCGTCGTAGCATTCATAGCAGCAATGCAATTACCGGCACCTGTAAAATGGGTAATGCCGGTGATAGCAGCTCAGTTGTTGATAACCAGCTGCGTGTGCATGGTGTGGAAGGTCTGCGTGTTGTTGATGCAAGCGTTGTTCCGAAAATTCCTGGTGGTCAGACAGGTGCTCCGGTGGTGATGATTGCAGAACGTGCAGCAGCCCTGCTGACCGGTAAAGCAACCATTGGTGCAAGCGCAGCCGCACCGGCAACCGTTGCAGCA**TAA**CTCGAGTCTGGTAAAGAAACCGCTGCTGCGAAATTTGAACGCCAGCACATGGACTCGTCTACTAGCGCAGCTTAATTAACCTAGGCTGCTGCCACCGCTGAGCAATAAAGCCCATGCAAGCTTGCGGCCGCGTCGTGACTGGGAAAACCCTGGCGACTAGTCTTGGACTCCTGTTGATAGATCCAGTAATGACCTCAGAACTCCATCTGGATTTGTTCAGAACGCTCGGTTGCCGCCGGGCGTTTTTTATTGGTGAGAATCCAGACGTTGTGTCTCAAAATCTCTGATGTTACATTGCACAAGATAAAAATATATCATCATGAACAATAAAACTGTCTGCTTACATAAACAGTAATACAAGGGGTGTTATGAGCCATATTCAACGGGAAACGTCTTGCTCGAGGCCGCGATTAAATTCCAACATGGATGCTGATTTATATGGGTATAAATGGGCTCGCGATAATGTCGGGCAATCAGGTGCGACAATCTATCGATTGTATGGGAAGCCCGATGCGCCAGAGTTGTTTCTGAAACATGGCAAAGGTAGCGTTGCCAATGATGTTACAGATGAGATGGTCAGACTAAACTGGCTGACGGAATTTATGCC

>pHal7-CvFAP_G462V_ GenBank:MW076827

TAGTGTAGCCGTAGTTAGGCCACCACTTCAAGAACTCTGTAGCACCGCCTACATACCTCGCTCTGCTAATCCTGTTACCAGTGGCTGCTGCCAGTGGCGATAAGTCGTGTCTTACCGGGTTGGACTCAAGACGATAGTTACCGGATAAGGCGCAGCGGTCGGGCTGAACGGGGGGTTCGTGCACACAGCCCAGCTTGGAGCGAACGACCTACACCGAACTGAGATACCTACAGCGTGAGCTATGAGAAAGCGCCACGCTTCCCGAAGGGAGAAAGGCGGACAGGCATCCGGTAAGCGGCAGGGTCGGAACAGGAGAGCGCACGAGGGAGCTTCCAGGGGGAAACGCCTGGTATCTTTATAGTCCTGTCGGGTTTCGCCACCTCTGACTTGAGCGTCGATTTTTGTGATGCTCGTCAGGGGGGCGGAGCCTATGGAAAAACGCCAGCAACGCGGCCGTGAAAGGCAGGCCGGTCCGTGGTGGCCACGGCCTCTAGGCCAGATCCAGCGGCATCTGGGTTAGTCGAGCGCGGGCCGCTTCCCATGTCTCACCAGGGCGAGCCTGTTTCGCGATCTCAGCATCTGAAATCTTCCCGGCCTTGCGCTTCGCTGGGGCCTTACCCACCGCCTTGGCGGGCTTCTTCGGTCCAAAACTGAACAACAGATGTGTGACCTTGCGCCCGGTCTTTCGCTGCGCCCACTCCACCTGTAGCGGGCTGTGCTCGTTGATCTGCGTCACGGCTGGATCAAGCACTCGCAACTTGAAGTCCTTGATCGAGGGATACCGGCCTTCCAGTTGAAACCACTTTCGCAGCTGGTCAATTTCTATTTCGCGCTGGCCGATGCTGTCCCATTGCATGAGCAGCTCGTAAAGCCTGATCGCGTGGGTGCTGTCCATCTTGGCCACGTCAGCCAAGGCGTATTTGGTGAACTGTTTGGTGAGTTCCGTCAGGTACGGCAGCATGTCTTTGGTGAACCTGAGTTCTACACGGCCCTCACCCTCCCGGTAGATGATTGTTTGCACCCAGCCGGTAATCATCACACTCGGTCTTTTCCCCTTGCCATTGGGCTCTTGGGTTAACCGGACTTCCCGCCGTTTCAGGCGCAGGGCCGCTTCTTTGAGCTGGTTGTAGGAAGATTCGATAGGGACACCCGCCATCGTCGCTATGTCCTCCGCCGTCACTGAATACATCACTTCATCGGTGACAGGCTCGCTCCTCTTCACCTGGCTAATACAGGCCAGAACGATCCGCTGTTCCTGAACACTGAGGCGATACGCGGCCTCGACCAGGGCATTGCTTTTGTAAACCATTGGGGGTGAGGCCACGTTCGACATTCCTTGTGTATAAGGGGACACTGTATCTGCGTCCCACAATACAACAAATCCGTCCCTTTACAACAACAAATCCGTCCCTTCTTAACAACAAATCCGTCCCTTAATGGCAACAAATCCGTCCCTTTTTAAACTCTAGAGGCCACGGATTACGTGGCCTGTAGACGTCCTAAAAGGTTTAAAAGGGAAAAGGAAGAAAAGGGTGGAAACGCAAAAAACGCACCACTACGTGGCCCCGTTGGGGCCGCATTTGTGCCCCTGAAGGGGCGGGGGAGGCGTCTGGGCAATCCCCGTTTTACCAGTCCCCTATCGCCGCCTGAGAGGGCGCAGGAAGCGAGTAATCAGGGTATCGAGGCGGATTCACCCTTGGCGTCCAACCAGCGGCACCAGCGGCGCCTGAGAGGGGCGCGCCCAGCTGTCTAGGGCGGCGGATTTGTCCTACTCAGGAGAGCGTTCACCGACAAACAACAGATAAAACGAAAGGCCCAGTCTTTCGACTGAGCCTTTCGTTTTATTTGATGCCTTTAATTAAAGCGGATAACAATTTCACACAGGAGGTTTTTCTATTGCGTACAACCGATAAAGGTATAGAGTTTGAGACTTTACTAGAGAAAGAGGAGAAATACTAG**ATG**GCCAGCGCAGTTGAAGATATTCGTAAAGTTCTGAGCGATAGCAGCAGTCCGGTTGCAGGTCAGAAATATGATTATATTCTGGTTGGTGGTGGCACCGCAGCATGTGTTCTGGCAAATCGTCTGAGCGCAGATGGTAGCAAACGTGTTCTGGTTCTGGAAGCAGGTCCGGATAATACCAGCCGTGATGTTAAAATTCCGGCAGCAATTACCCGTCTGTTTCGTAGTCCGCTGGATTGGAACCTGTTTAGCGAACTGCAAGAACAGCTGGCAGAACGTCAGATTTATATGGCACGTGGTCGTCTGTTAGGTGGTAGCAGCGCAACCAATGCAACCCTGTATCATCGTGGTGCAGCCGGTGATTATGATGCATGGGGTGTTGAAGGTTGGAGCAGCGAAGATGTTCTGAGCTGGTTTGTTCAGGCAGAAACCAATGCCGATTTTGGTCCGGGTGCATATCATGGTAGCGGTGGTCCGATGCGTGTTGAAAATCCGCGTTATACCAATAAACAGCTGCATACCGCATTTTTCAAAGCAGCAGAAGAAGTTGGTCTGACCCCGAATAGCGATTTTAATGATTGGAGCCATGATCACGCAGGTTATGGCACCTTTCAGGTTATGCAGGATAAAGGCACCCGTGCAGATATGTATCGTCAGTATCTGAAACCGGTTCTGGGTCGTCGTAATCTGCAGGTTCTGACCGGTGCAGCAGTTACCAAAGTTAATATTGATCAGGCAGCAGGTAAAGCACAGGCACTGGGCGTTGAATTTTCAACCGATGGTCCGACCGGTGAACGTCTGTCAGCAGAACTGGCACCTGGTGGTGAAGTTATTATGTGTGCCGGTGCAGTTCATACCCCGTTTCTGCTGAAACATAGCGGTGTTGGTCCGAGCGCAGAACTGAAAGAATTTGGTATTCCGGTTGTTAGCAATCTGGCAGGCGTTGGCCAGAATCTGCAAGATCAGCCTGCATGTCTGACCGCAGCACCGGTTAAAGAAAAATATGATGGTATTGCCATCAGCGACCACATCTATAATGAAAAAGGTCAGATTCGCAAACGTGCCATTGCAAGCTATCTGCTTGGTGGTCGTGGTGGCCTGACCAGTACCGGTTGTGATCGCGGTGCATTTGTTCGTACCGCAGGTCAGGCACTGCCGGATCTGCAAGTTCGTTTTGTTCCTGGTATGGCACTGGATCCGGATGTTGTTAGCACCTATGTGCGTTTTGCCAAATTTCAGAGCCAGGGCCTGAAATGGCCGAGCGGTATTACCATGCAGCTGATTGCATGTCGTCCGCAGAGTACCGGTAGCGTGGGTCTGAAAAGCGCAGATCCGTTTGCACCGCCTAAACTGAGTCCGGGTTATCTGACCGATAAAGATGGTGCAGATCTGGCGACCCTGCGTAAAGGTATTCATTGGGCACGTGATGTTGCACGTAGCTCAGCACTGAGCGAATATCTGGATGGTGAACTGTTTCCTGGTAGTGGTGTTGTTAGTGATGATCAGATCGATGAATATATCCGTCGTAGCATTCATAGCAGCAATGCAATTACCGGCACCTGTAAAATGGGTAATGCCGGTGATAGCAGCTCAGTTGTTGATAACCAGCTGCGTGTGCATGGTGTGGAAGGTCTGCGTGTTGTTGATGCAAGCGTTGTTCCGAAAATTCCTGGTGGTCAGACAGGTGCTCCGGTGGTGATGATTGCAGAACGTGCAGCAGCCCTGCTGACCGGTAAAGCAACCATTGGTGCAAGCGCAGCCGCACCGGCAACCGTTGCAGCA**TAA**TTAACCTAGGCTGCTGCCACCGCTGAGCAATAAAGCCCATGCAAGCTTGCGGCCGCGTCGTGACTGGGAAAACCCTGGCGACTAGTCTTGGACTCCTGTTGATAGATCCAGTAATGACCTCAGAACTCCATCTGGATTTGTTCAGAACGCTCGGTTGCCGCCGGGCGTTTTTTATTGGTGAGAATCCAGACGTTGTGTCTCAAAATCTCTGATGTTACATTGCACAAGATAAAAATATATCATCATGAACAATAAAACTGTCTGCTTACATAAACAGTAATACAAGGGGTGTTATGAGCCATATTCAACGGGAAACGTCTTGCTCGAGGCCGCGATTAAATTCCAACATGGATGCTGATTTATATGGGTATAAATGGGCTCGCGATAATGTCGGGCAATCAGGTGCGACAATCTATCGATTGTATGGGAAGCCCGATGCGCCAGAGTTGTTTCTGAAACATGGCAAAGGTAGCGTTGCCAATGATGTTACAGATGAGATGGTCAGACTAAACTGGCTGACGGAATTTATGCCTCTTCCGACCATCAAGCATTTTATCCGTACTCCTGATGATGCATGGTTACTCACCACTGCGATCCCCGGGAAAACAGCATTCCAGGTATTAGAAGAATATCCTGATTCAGGTGAAAATATTGTTGATGCGCTGGCAGTGTTCCTGCGCCGGTTGCATTCGATTCCTGTTTGTAATTGTCCTTTTAACAGCGATCGCGTATTTCGTCTCGCTCAGGCGCAATCACGAATGAATAACGGTTTGGTTGATGCGAGTGATTTTGATGACGAGCGTAATGGCTGGCCTGTTGAACAAGTCTGGAAAGAAATGCATAAGCTTTTGCCATTCTCACCGGATTCAGTCGTCACTCATGGTGATTTCTCACTTGATAACCTTATTTTTGACGAGGGGAAATTAATAGGTTGTATTGATGTTGGACGAGTCGGAATCGCAGACCGATACCAGGATCTTGCCATCCTATGGAACTGCCTCGGTGAGTTTTCTCCTTCATTACAGAAACGGCTTTTTCAAAAATATGGTATTGATAATCCTGATATGAATAAATTGCAGTTTCATTTGATGCTCGATGAGTTTTTCTAATCAGAATTGGTTAATTGGTTGTAACACTGGCAGAGCATTACGCTGACTTGACGGGACGGCGGCTTTGTTGAATAAATCGAACTTTTGCTGAGTTGAAGGATCAGATCACGCATCTTCCCGACAACGCAGACCGTTCCGTGGCAAAGCAAAAGTTCAAAATCACCAACTGGTCCACCTACAACAAAGCTCTCATCAACCGTGGCTCCCTCACTTTCTGGCTGGATGATGGGGCGATTCAGGCCTGGTATGAGTCAGCAACACCTTCTTCACGAGGCAGACCTCAGCGCTATTCTGACCTTGCCATCACGACTGTGCTGGTCATTAAACGCGTATTCAGGCTGACCCTGCGCGCTGCGCAGGGCTTTATTGATTCCATTTTTACACTGATGAATGTTCCGTTGCGCTGCCCGGATTACAGCCGGATCCTCTAGAGTCGACCTGCAGGCATGCTGATCGGCACGTAAGAGGTTCCAACTTTCACCATAATGAAATAAGATCACTACCGGGCGTATTTTTTGAGTTATCGAGATTTTCAGGAGCTAAGGAAGCTAAAATGCGCTCACGCAACTGGTCCAGAACCTTGACCGAACGCAGCGGTGGTAACGGCGCAGTGGCGGTTTTCATGGCTTGTTATGACTGTTTTTTTGGGGTACAGTCTATGCCTCGGGCATCCAAGCAGCAAGCGCGTTACGCCGTGGGTCGATGTTTGATGTTATGGAGCAGCAACGATGTTACGCAGCAGGGCAGTCGCCCTAAAACAAAGTTAAACATCATGAGGGAAGCGGTGATCGCCGAAGTATCGACTCAACTATCAGAGGTAGTTGGCGTCATCGAGCGCCATCTCGAACCGACGTTGCTGGCCGTACATTTGTACGGCTCCGCAGTGGATGGCGGCCTGAAGCCACACAGTGATATTGATTTGCTGGTTACGGTGACCGTAAGGCTTGATGAAACAACGCGGCGAGCTTTGATCAACGACCTTTTGGAAACTTCGGCTTCCCCTGGAGAGAGCGAGATTCTCCGCGCTGTAGAAGTCACCATTGTTGTGCACGACGACATCATTCCGTGGCGTTATCCAGCTAAGCGCGAACTGCAATTTGGAGAATGGCAGCGCAATGACATTCTTGCAGGTATCTTCGAGCCAGCCACGATCGACATTGATCTGGCTATCTTGCTGACAAAAGCAAGAGAACATAGCGTTGCCTTGGTAGGTCCAGCGGCGGAGGAACTCTTTGATCCGGTTCCTGAACAGGATCTATTTGAGGCGCTAAATGAAACCTTAACGCTATGGAACTCGCCGCCCGACTGGGCTGGCGATGAGCGAAATGTAGTGCTTACGTTGTCCCGCATTTGGTACAGCGCAGTAACCGGCAAAATCGCGCCGAAGGATGTCGCTGCCGACTGGGCAATGGAGCGCCTGCCGGCCCAGTATCAGCCCGTCATACTTGAAGCTAGACAGGCTTATCTTGGACAAGAAGAAGATCGCTTGGCCTCGCGCGCAGATCAGTTGGAAGAATTTGTCCACTACGTGAAAGGCGAGATCACCAAGGTAGTCGGCAAATAAACTAGTAAATAATAAAAAAGCCGGATTAATAATCTGGCTTTTTATATTCTCTGCATAACCCTGCTTCGGGGTCATTATAGCGATTTTTTCGGTATATCCATCCTTTTTCGCACGATATACAGGATTTTGCCAAAGGGTTCGTGTAGACTTTCCTTGGTGTATCCAACGGCGTCAGCCGGGCAGGATAGGTGAAGTAGGCCCACCCGCGAGCGGGTGTTCCTTCTTCACTGTCCCTTATTCGCACCTGGCGGTGCTCAACGGGAATCCTGCTCTGCGAGGCTGGCCGTAGGCCGGCCGATAATCTCATGACCAAAATCCCTTAACGTGAGTTTTCGTTCCACTGAGCGTCAGACCCCGTAGAAAAGATCAAAGGATCTTCTTGAGATCCTTTTTTTCTGCGCGTAATCTGCTGCTTGCAAACAAAAAAACCACCGCTACCAGCGGTGGTTTGTTTGCCGGATCAAGAGCTACCAACTCTTTTTCCGAAGGTAACTGGCTTCAGCAGAGCGCAGATACCAAATACTGTTCTTC

>pHal2-FAP_G462V_RFP GenBank:MW076828

TCTTCCGACCATCAAGCATTTTATCCGTACTCCTGATGATGCATGGTTACTCACCACTGCGATCCCCGGGAAAACAGCATTCCAGGTATTAGAAGAATATCCTGATTCAGGTGAAAATATTGTTGATGCGCTGGCAGTGTTCCTGCGCCGGTTGCATTCGATTCCTGTTTGTAATTGTCCTTTTAACAGCGATCGCGTATTTCGTCTCGCTCAGGCGCAATCACGAATGAATAACGGTTTGGTTGATGCGAGTGATTTTGATGACGAGCGTAATGGCTGGCCTGTTGAACAAGTCTGGAAAGAAATGCATAAGCTTTTGCCATTCTCACCGGATTCAGTCGTCACTCATGGTGATTTCTCACTTGATAACCTTATTTTTGACGAGGGGAAATTAATAGGTTGTATTGATGTTGGACGAGTCGGAATCGCAGACCGATACCAGGATCTTGCCATCCTATGGAACTGCCTCGGTGAGTTTTCTCCTTCATTACAGAAACGGCTTTTTCAAAAATATGGTATTGATAATCCTGATATGAATAAATTGCAGTTTCATTTGATGCTCGATGAGTTTTTCTAATCAGAATTGGTTAATTGGTTGTAACACTGGCAGAGCATTACGCTGACTTGACGGGACGGCGGCTTTGTTGAATAAATCGAACTTTTGCTGAGTTGAAGGATCAGATCACGCATCTTCCCGACAACGCAGACCGTTCCGTGGCAAAGCAAAAGTTCAAAATCACCAACTGGTCCACCTACAACAAAGCTCTCATCAACCGTGGCTCCCTCACTTTCTGGCTGGATGATGGGGCGATTCAGGCCTGGTATGAGTCAGCAACACCTTCTTCACGAGGCAGACCTCAGCGCTATTCTGACCTTGCCATCACGACTGTGCTGGTCATTAAACGCGTATTCAGGCTGACCCTGCGCGCTGCGCAGGGCTTTATTGATTCCATTTTTACACTGATGAATGTTCCGTTGCGCTGCCCGGATTACAGCCGGATCCTCTAGAGTCGACCTGCAGGCATGCTGATCGGCACGTAAGAGGTTCCAACTTTCACCATAATGAAATAAGATCACTACCGGGCGTATTTTTTGAGTTATCGAGATTTTCAGGAGCTAAGGAAGCTAAAATGCGCTCACGCAACTGGTCCAGAACCTTGACCGAACGCAGCGGTGGTAACGGCGCAGTGGCGGTTTTCATGGCTTGTTATGACTGTTTTTTTGGGGTACAGTCTATGCCTCGGGCATCCAAGCAGCAAGCGCGTTACGCCGTGGGTCGATGTTTGATGTTATGGAGCAGCAACGATGTTACGCAGCAGGGCAGTCGCCCTAAAACAAAGTTAAACATCATGAGGGAAGCGGTGATCGCCGAAGTATCGACTCAACTATCAGAGGTAGTTGGCGTCATCGAGCGCCATCTCGAACCGACGTTGCTGGCCGTACATTTGTACGGCTCCGCAGTGGATGGCGGCCTGAAGCCACACAGTGATATTGATTTGCTGGTTACGGTGACCGTAAGGCTTGATGAAACAACGCGGCGAGCTTTGATCAACGACCTTTTGGAAACTTCGGCTTCCCCTGGAGAGAGCGAGATTCTCCGCGCTGTAGAAGTCACCATTGTTGTGCACGACGACATCATTCCGTGGCGTTATCCAGCTAAGCGCGAACTGCAATTTGGAGAATGGCAGCGCAATGACATTCTTGCAGGTATCTTCGAGCCAGCCACGATCGACATTGATCTGGCTATCTTGCTGACAAAAGCAAGAGAACATAGCGTTGCCTTGGTAGGTCCAGCGGCGGAGGAACTCTTTGATCCGGTTCCTGAACAGGATCTATTTGAGGCGCTAAATGAAACCTTAACGCTATGGAACTCGCCGCCCGACTGGGCTGGCGATGAGCGAAATGTAGTGCTTACGTTGTCCCGCATTTGGTACAGCGCAGTAACCGGCAAAATCGCGCCGAAGGATGTCGCTGCCGACTGGGCAATGGAGCGCCTGCCGGCCCAGTATCAGCCCGTCATACTTGAAGCTAGACAGGCTTATCTTGGACAAGAAGAAGATCGCTTGGCCTCGCGCGCAGATCAGTTGGAAGAATTTGTCCACTACGTGAAAGGCGAGATCACCAAGGTAGTCGGCAAATAAACTAGTAAATAATAAAAAAGCCGGATTAATAATCTGGCTTTTTATATTCTCTGCATAACCCTGCTTCGGGGTCATTATAGCGATTTTTTCGGTATATCCATCCTTTTTCGCACGATATACAGGATTTTGCCAAAGGGTTCGTGTAGACTTTCCTTGGTGTATCCAACGGCGTCAGCCGGGCAGGATAGGTGAAGTAGGCCCACCCGCGAGCGGGTGTTCCTTCTTCACTGTCCCTTATTCGCACCTGGCGGTGCTCAACGGGAATCCTGCTCTGCGAGGCTGGCCGTAGGCCGGCCGATAATCTCATGACCAAAATCCCTTAACGTGAGTTTTCGTTCCACTGAGCGTCAGACCCCGTAGAAAAGATCAAAGGATCTTCTTGAGATCCTTTTTTTCTGCGCGTAATCTGCTGCTTGCAAACAAAAAAACCACCGCTACCAGCGGTGGTTTGTTTGCCGGATCAAGAGCTACCAACTCTTTTTCCGAAGGTAACTGGCTTCAGCAGAGCGCAGATACCAAATACTGTTCTTCTAGTGTAGCCGTAGTTAGGCCACCACTTCAAGAACTCTGTAGCACCGCCTACATACCTCGCTCTGCTAATCCTGTTACCAGTGGCTGCTGCCAGTGGCGATAAGTCGTGTCTTACCGGGTTGGACTCAAGACGATAGTTACCGGATAAGGCGCAGCGGTCGGGCTGAACGGGGGGTTCGTGCACACAGCCCAGCTTGGAGCGAACGACCTACACCGAACTGAGATACCTACAGCGTGAGCTATGAGAAAGCGCCACGCTTCCCGAAGGGAGAAAGGCGGACAGGCATCCGGTAAGCGGCAGGGTCGGAACAGGAGAGCGCACGAGGGAGCTTCCAGGGGGAAACGCCTGGTATCTTTATAGTCCTGTCGGGTTTCGCCACCTCTGACTTGAGCGTCGATTTTTGTGATGCTCGTCAGGGGGGCGGAGCCTATGGAAAAACGCCAGCAACGCGGCCGTGAAAGGCAGGCCGGTCCGTGGTGGCCACGGCCTCTAGGCCAGATCCAGCGGCATCTGGGTTAGTCGAGCGCGGGCCGCTTCCCATGTCTCACCAGGGCGAGCCTGTTTCGCGATCTCAGCATCTGAAATCTTCCCGGCCTTGCGCTTCGCTGGGGCCTTACCCACCGCCTTGGCGGGCTTCTTCGGTCCAAAACTGAACAACAGATGTGTGACCTTGCGCCCGGTCTTTCGCTGCGCCCACTCCACCTGTAGCGGGCTGTGCTCGTTGATCTGCGTCACGGCTGGATCAAGCACTCGCAACTTGAAGTCCTTGATCGAGGGATACCGGCCTTCCAGTTGAAACCACTTTCGCAGCTGGTCAATTTCTATTTCGCGCTGGCCGATGCTGTCCCATTGCATGAGCAGCTCGTAAAGCCTGATCGCGTGGGTGCTGTCCATCTTGGCCACGTCAGCCAAGGCGTATTTGGTGAACTGTTTGGTGAGTTCCGTCAGGTACGGCAGCATGTCTTTGGTGAACCTGAGTTCTACACGGCCCTCACCCTCCCGGTAGATGATTGTTTGCACCCAGCCGGTAATCATCACACTCGGTCTTTTCCCCTTGCCATTGGGCTCTTGGGTTAACCGGACTTCCCGCCGTTTCAGGCGCAGGGCCGCTTCTTTGAGCTGGTTGTAGGAAGATTCGATAGGGACACCCGCCATCGTCGCTATGTCCTCCGCCGTCACTGAATACATCACTTCATCGGTGACAGGCTCGCTCCTCTTCACCTGGCTAATACAGGCCAGAACGATCCGCTGTTCCTGAACACTGAGGCGATACGCGGCCTCGACCAGGGCATTGCTTTTGTAAACCATTGGGGGTGAGGCCACGTTCGACATTCCTTGTGTATAAGGGGACACTGTATCTGCGTCCCACAATACAACAAATCCGTCCCTTTACAACAACAAATCCGTCCCTTCTTAACAACAAATCCGTCCCTTAATGGCAACAAATCCGTCCCTTTTTAAACTCTAGAGGCCACGGATTACGTGGCCTGTAGACGTCCTAAAAGGTTTAAAAGGGAAAAGGAAGAAAAGGGTGGAAACGCAAAAAACGCACCACTACGTGGCCCCGTTGGGGCCGCATTTGTGCCCCTGAAGGGGCGGGGGAGGCGTCTGGGCAATCCCCGTTTTACCAGTCCCCTATCGCCGCCTGAGAGGGCGCAGGAAGCGAGTAATCAGGGTATCGAGGCGGATTCACCCTTGGCGTCCAACCAGCGGCACCAGCGGCGCCTGAGAGGGGCGCGCCCAGCTGTCTAGGGCGGCGGATTTGTCCTACTCAGGAGAGCGTTCACCGACAAACAACAGATAAAACGAAAGGCCCAGTCTTTCGACTGAGCCTTTCGTTTTATTTGATGCCTTTAATTAAAGCGGATAACAATTTCACACAGGAGGCCGCCTAGGCAAGGATGCCTCCACACCGCTCGTCACATCCTGCCCATGAGTTAATTATATTTGTGGCATTATAGGGAATTGTGAGCGCTCACAATTAGCTGTCACCGGATGTGCTTTCCGGTCTGATGAGTCCGTGAGGACGAAACAGCCTCTACAAATAATTTTGTTTAATACTAGAGAAAGAGGAGAAATACTAGTTTGTTTAACTTTAAGAAGGAGATATACC**ATG**GCCAGCGCAGTTGAAGATATTCGTAAAGTTCTGAGCGATAGCAGCAGTCCGGTTGCAGGTCAGAAATATGATTATATTCTGGTTGGTGGTGGCACCGCAGCATGTGTTCTGGCAAATCGTCTGAGCGCAGATGGTAGCAAACGTGTTCTGGTTCTGGAAGCAGGTCCGGATAATACCAGCCGTGATGTTAAAATTCCGGCAGCAATTACCCGTCTGTTTCGTAGTCCGCTGGATTGGAACCTGTTTAGCGAACTGCAAGAACAGCTGGCAGAACGTCAGATTTATATGGCACGTGGTCGTCTGTTAGGTGGTAGCAGCGCAACCAATGCAACCCTGTATCATCGTGGTGCAGCCGGTGATTATGATGCATGGGGTGTTGAAGGTTGGAGCAGCGAAGATGTTCTGAGCTGGTTTGTTCAGGCAGAAACCAATGCCGATTTTGGTCCGGGTGCATATCATGGTAGCGGTGGTCCGATGCGTGTTGAAAATCCGCGTTATACCAATAAACAGCTGCATACCGCATTTTTCAAAGCAGCAGAAGAAGTTGGTCTGACCCCGAATAGCGATTTTAATGATTGGAGCCATGATCACGCAGGTTATGGCACCTTTCAGGTTATGCAGGATAAAGGCACCCGTGCAGATATGTATCGTCAGTATCTGAAACCGGTTCTGGGTCGTCGTAATCTGCAGGTTCTGACCGGTGCAGCAGTTACCAAAGTTAATATTGATCAGGCAGCAGGTAAAGCACAGGCACTGGGCGTTGAATTTTCAACCGATGGTCCGACCGGTGAACGTCTGTCAGCAGAACTGGCACCTGGTGGTGAAGTTATTATGTGTGCCGGTGCAGTTCATACCCCGTTTCTGCTGAAACATAGCGGTGTTGGTCCGAGCGCAGAACTGAAAGAATTTGGTATTCCGGTTGTTAGCAATCTGGCAGGCGTTGGCCAGAATCTGCAAGATCAGCCTGCATGTCTGACCGCAGCACCGGTTAAAGAAAAATATGATGGTATTGCCATCAGCGACCACATCTATAATGAAAAAGGTCAGATTCGCAAACGTGCCATTGCAAGCTATCTGCTTGGTGGTCGTGGTGGCCTGACCAGTACCGGTTGTGATCGCGGTGCATTTGTTCGTACCGCAGGTCAGGCACTGCCGGATCTGCAAGTTCGTTTTGTTCCTGGTATGGCACTGGATCCGGATGTTGTTAGCACCTATGTGCGTTTTGCCAAATTTCAGAGCCAGGGCCTGAAATGGCCGAGCGGTATTACCATGCAGCTGATTGCATGTCGTCCGCAGAGTACCGGTAGCGTGGGTCTGAAAAGCGCAGATCCGTTTGCACCGCCTAAACTGAGTCCGGGTTATCTGACCGATAAAGATGGTGCAGATCTGGCGACCCTGCGTAAAGGTATTCATTGGGCACGTGATGTTGCACGTAGCTCAGCACTGAGCGAATATCTGGATGGTGAACTGTTTCCTGGTAGTGGTGTTGTTAGTGATGATCAGATCGATGAATATATCCGTCGTAGCATTCATAGCAGCAATGCAATTACCGGCACCTGTAAAATGGGTAATGCCGGTGATAGCAGCTCAGTTGTTGATAACCAGCTGCGTGTGCATGGTGTGGAAGGTCTGCGTGTTGTTGATGCAAGCGTTGTTCCGAAAATTCCTGGTGGTCAGACAGGTGCTCCGGTGGTGATGATTGCAGAACGTGCAGCAGCCCTGCTGACCGGTAAAGCAACCATTGGTGCAAGCGCAGCCGCACCGGCAACCGTTGCAGCAGGTTCTGCGGGTTCTGCGGCCGGTTCTGGCGAATTTATGGCGAGTAGCGAAGACGTTATCAAAGAGTTCATGCGTTTCAAAGTTCGTATGGAAGGTTCCGTTAACGGTCACGAGTTCGAAATCGAAGGTGAAGGTGAAGGTCGTCCGTACGAAGGTACCCAGACCGCTAAACTGAAAGTTACCAAAGGTGGTCCGCTGCCGTTCGCTTGGGACATCCTGTCCCCGCAGTTCCAGTACGGTTCCAAAGCTTACGTTAAACACCCGGCTGACATCCCGGACTACCTGAAACTGTCCTTCCCGGAAGGTTTCAAATGGGAACGTGTTATGAACTTCGAAGACGGTGGTGTTGTTACCGTTACCCAGGACTCCTCCCTGCAAGACGGTGAGTTCATCTACAAAGTTAAACTGCGTGGTACCAACTTCCCGTCCGACGGTCCGGTTATGCAGAAAAAAACCATGGGTTGGGAAGCTTCCACCGAACGTATGTACCCGGAAGACGGTGCTCTGAAAGGTGAAATCAAAATGCGTCTGAAACTGAAAGACGGTGGTCACTACGACGCTGAAGTTAAAACCACCTACATGGCTAAAAAACCGGTTCAGCTGCCGGGTGCTTACAAAACCGACATCAAACTGGACATCACCTCCCACAACGAAGACTACACCATCGTTGAACAGTACGAACGTGCTGAAGGTCGTCACTCCACCGGTGCT**TAA**CTCGAGTCTGGTAAAGAAACCGCTGCTGCGAAATTTGAACGCCAGCACATGGACTCGTCTACTAGCGCAGCTTAATTAACCTAGGCTGCTGCCACCGCTGAGCAATAAAGCCCATGCAAGCTTGCGGCCGCGTCGTGACTGGGAAAACCCTGGCGACTAGTCTTGGACTCCTGTTGATAGATCCAGTAATGACCTCAGAACTCCATCTGGATTTGTTCAGAACGCTCGGTTGCCGCCGGGCGTTTTTTATTGGTGAGAATCCAGACGTTGTGTCTCAAAATCTCTGATGTTACATTGCACAAGATAAAAATATATCATCATGAACAATAAAACTGTCTGCTTACATAAACAGTAATACAAGGGGTGTTATGAGCCATATTCAACGGGAAACGTCTTGCTCGAGGCCGCGATTAAATTCCAACATGGATGCTGATTTATATGGGTATAAATGGGCTCGCGATAATGTCGGGCAATCAGGTGCGACAATCTATCGATTGTATGGGAAGCCCGATGCGCCAGAGTTGTTTCTGAAACATGGCAAAGGTAGCGTTGCCAATGATGTTACAGATGAGATGGTCAGACTAAACTGGCTGACGGAATTTATGCC

>pHal7-FAP_G462V_RFP GenBank:MW076829

TAGTGTAGCCGTAGTTAGGCCACCACTTCAAGAACTCTGTAGCACCGCCTACATACCTCGCTCTGCTAATCCTGTTACCAGTGGCTGCTGCCAGTGGCGATAAGTCGTGTCTTACCGGGTTGGACTCAAGACGATAGTTACCGGATAAGGCGCAGCGGTCGGGCTGAACGGGGGGTTCGTGCACACAGCCCAGCTTGGAGCGAACGACCTACACCGAACTGAGATACCTACAGCGTGAGCTATGAGAAAGCGCCACGCTTCCCGAAGGGAGAAAGGCGGACAGGCATCCGGTAAGCGGCAGGGTCGGAACAGGAGAGCGCACGAGGGAGCTTCCAGGGGGAAACGCCTGGTATCTTTATAGTCCTGTCGGGTTTCGCCACCTCTGACTTGAGCGTCGATTTTTGTGATGCTCGTCAGGGGGGCGGAGCCTATGGAAAAACGCCAGCAACGCGGCCGTGAAAGGCAGGCCGGTCCGTGGTGGCCACGGCCTCTAGGCCAGATCCAGCGGCATCTGGGTTAGTCGAGCGCGGGCCGCTTCCCATGTCTCACCAGGGCGAGCCTGTTTCGCGATCTCAGCATCTGAAATCTTCCCGGCCTTGCGCTTCGCTGGGGCCTTACCCACCGCCTTGGCGGGCTTCTTCGGTCCAAAACTGAACAACAGATGTGTGACCTTGCGCCCGGTCTTTCGCTGCGCCCACTCCACCTGTAGCGGGCTGTGCTCGTTGATCTGCGTCACGGCTGGATCAAGCACTCGCAACTTGAAGTCCTTGATCGAGGGATACCGGCCTTCCAGTTGAAACCACTTTCGCAGCTGGTCAATTTCTATTTCGCGCTGGCCGATGCTGTCCCATTGCATGAGCAGCTCGTAAAGCCTGATCGCGTGGGTGCTGTCCATCTTGGCCACGTCAGCCAAGGCGTATTTGGTGAACTGTTTGGTGAGTTCCGTCAGGTACGGCAGCATGTCTTTGGTGAACCTGAGTTCTACACGGCCCTCACCCTCCCGGTAGATGATTGTTTGCACCCAGCCGGTAATCATCACACTCGGTCTTTTCCCCTTGCCATTGGGCTCTTGGGTTAACCGGACTTCCCGCCGTTTCAGGCGCAGGGCCGCTTCTTTGAGCTGGTTGTAGGAAGATTCGATAGGGACACCCGCCATCGTCGCTATGTCCTCCGCCGTCACTGAATACATCACTTCATCGGTGACAGGCTCGCTCCTCTTCACCTGGCTAATACAGGCCAGAACGATCCGCTGTTCCTGAACACTGAGGCGATACGCGGCCTCGACCAGGGCATTGCTTTTGTAAACCATTGGGGGTGAGGCCACGTTCGACATTCCTTGTGTATAAGGGGACACTGTATCTGCGTCCCACAATACAACAAATCCGTCCCTTTACAACAACAAATCCGTCCCTTCTTAACAACAAATCCGTCCCTTAATGGCAACAAATCCGTCCCTTTTTAAACTCTAGAGGCCACGGATTACGTGGCCTGTAGACGTCCTAAAAGGTTTAAAAGGGAAAAGGAAGAAAAGGGTGGAAACGCAAAAAACGCACCACTACGTGGCCCCGTTGGGGCCGCATTTGTGCCCCTGAAGGGGCGGGGGAGGCGTCTGGGCAATCCCCGTTTTACCAGTCCCCTATCGCCGCCTGAGAGGGCGCAGGAAGCGAGTAATCAGGGTATCGAGGCGGATTCACCCTTGGCGTCCAACCAGCGGCACCAGCGGCGCCTGAGAGGGGCGCGCCCAGCTGTCTAGGGCGGCGGATTTGTCCTACTCAGGAGAGCGTTCACCGACAAACAACAGATAAAACGAAAGGCCCAGTCTTTCGACTGAGCCTTTCGTTTTATTTGATGCCTTTAATTAAAGCGGATAACAATTTCACACAGGAGGTTTTTCTATTGCGTACAACCGATAAAGGTATAGAGTTTGAGACTTTACTAGAGAAAGAGGAGAAATACTAG**ATG**GCCAGCGCAGTTGAAGATATTCGTAAAGTTCTGAGCGATAGCAGCAGTCCGGTTGCAGGTCAGAAATATGATTATATTCTGGTTGGTGGTGGCACCGCAGCATGTGTTCTGGCAAATCGTCTGAGCGCAGATGGTAGCAAACGTGTTCTGGTTCTGGAAGCAGGTCCGGATAATACCAGCCGTGATGTTAAAATTCCGGCAGCAATTACCCGTCTGTTTCGTAGTCCGCTGGATTGGAACCTGTTTAGCGAACTGCAAGAACAGCTGGCAGAACGTCAGATTTATATGGCACGTGGTCGTCTGTTAGGTGGTAGCAGCGCAACCAATGCAACCCTGTATCATCGTGGTGCAGCCGGTGATTATGATGCATGGGGTGTTGAAGGTTGGAGCAGCGAAGATGTTCTGAGCTGGTTTGTTCAGGCAGAAACCAATGCCGATTTTGGTCCGGGTGCATATCATGGTAGCGGTGGTCCGATGCGTGTTGAAAATCCGCGTTATACCAATAAACAGCTGCATACCGCATTTTTCAAAGCAGCAGAAGAAGTTGGTCTGACCCCGAATAGCGATTTTAATGATTGGAGCCATGATCACGCAGGTTATGGCACCTTTCAGGTTATGCAGGATAAAGGCACCCGTGCAGATATGTATCGTCAGTATCTGAAACCGGTTCTGGGTCGTCGTAATCTGCAGGTTCTGACCGGTGCAGCAGTTACCAAAGTTAATATTGATCAGGCAGCAGGTAAAGCACAGGCACTGGGCGTTGAATTTTCAACCGATGGTCCGACCGGTGAACGTCTGTCAGCAGAACTGGCACCTGGTGGTGAAGTTATTATGTGTGCCGGTGCAGTTCATACCCCGTTTCTGCTGAAACATAGCGGTGTTGGTCCGAGCGCAGAACTGAAAGAATTTGGTATTCCGGTTGTTAGCAATCTGGCAGGCGTTGGCCAGAATCTGCAAGATCAGCCTGCATGTCTGACCGCAGCACCGGTTAAAGAAAAATATGATGGTATTGCCATCAGCGACCACATCTATAATGAAAAAGGTCAGATTCGCAAACGTGCCATTGCAAGCTATCTGCTTGGTGGTCGTGGTGGCCTGACCAGTACCGGTTGTGATCGCGGTGCATTTGTTCGTACCGCAGGTCAGGCACTGCCGGATCTGCAAGTTCGTTTTGTTCCTGGTATGGCACTGGATCCGGATGTTGTTAGCACCTATGTGCGTTTTGCCAAATTTCAGAGCCAGGGCCTGAAATGGCCGAGCGGTATTACCATGCAGCTGATTGCATGTCGTCCGCAGAGTACCGGTAGCGTGGGTCTGAAAAGCGCAGATCCGTTTGCACCGCCTAAACTGAGTCCGGGTTATCTGACCGATAAAGATGGTGCAGATCTGGCGACCCTGCGTAAAGGTATTCATTGGGCACGTGATGTTGCACGTAGCTCAGCACTGAGCGAATATCTGGATGGTGAACTGTTTCCTGGTAGTGGTGTTGTTAGTGATGATCAGATCGATGAATATATCCGTCGTAGCATTCATAGCAGCAATGCAATTACCGGCACCTGTAAAATGGGTAATGCCGGTGATAGCAGCTCAGTTGTTGATAACCAGCTGCGTGTGCATGGTGTGGAAGGTCTGCGTGTTGTTGATGCAAGCGTTGTTCCGAAAATTCCTGGTGGTCAGACAGGTGCTCCGGTGGTGATGATTGCAGAACGTGCAGCAGCCCTGCTGACCGGTAAAGCAACCATTGGTGCAAGCGCAGCCGCACCGGCAACCGTTGCAGCAGGTTCTGCGGGTTCTGCGGCCGGTTCTGGCGAATTTATGGCGAGTAGCGAAGACGTTATCAAAGAGTTCATGCGTTTCAAAGTTCGTATGGAAGGTTCCGTTAACGGTCACGAGTTCGAAATCGAAGGTGAAGGTGAAGGTCGTCCGTACGAAGGTACCCAGACCGCTAAACTGAAAGTTACCAAAGGTGGTCCGCTGCCGTTCGCTTGGGACATCCTGTCCCCGCAGTTCCAGTACGGTTCCAAAGCTTACGTTAAACACCCGGCTGACATCCCGGACTACCTGAAACTGTCCTTCCCGGAAGGTTTCAAATGGGAACGTGTTATGAACTTCGAAGACGGTGGTGTTGTTACCGTTACCCAGGACTCCTCCCTGCAAGACGGTGAGTTCATCTACAAAGTTAAACTGCGTGGTACCAACTTCCCGTCCGACGGTCCGGTTATGCAGAAAAAAACCATGGGTTGGGAAGCTTCCACCGAACGTATGTACCCGGAAGACGGTGCTCTGAAAGGTGAAATCAAAATGCGTCTGAAACTGAAAGACGGTGGTCACTACGACGCTGAAGTTAAAACCACCTACATGGCTAAAAAACCGGTTCAGCTGCCGGGTGCTTACAAAACCGACATCAAACTGGACATCACCTCCCACAACGAAGACTACACCATCGTTGAACAGTACGAACGTGCTGAAGGTCGTCACTCCACCGGTGCT**TAA**TTAACCTAGGCTGCTGCCACCGCTGAGCAATAAAGCCCATGCAAGCTTGCGGCCGCGTCGTGACTGGGAAAACCCTGGCGACTAGTCTTGGACTCCTGTTGATAGATCCAGTAATGACCTCAGAACTCCATCTGGATTTGTTCAGAACGCTCGGTTGCCGCCGGGCGTTTTTTATTGGTGAGAATCCAGACGTTGTGTCTCAAAATCTCTGATGTTACATTGCACAAGATAAAAATATATCATCATGAACAATAAAACTGTCTGCTTACATAAACAGTAATACAAGGGGTGTTATGAGCCATATTCAACGGGAAACGTCTTGCTCGAGGCCGCGATTAAATTCCAACATGGATGCTGATTTATATGGGTATAAATGGGCTCGCGATAATGTCGGGCAATCAGGTGCGACAATCTATCGATTGTATGGGAAGCCCGATGCGCCAGAGTTGTTTCTGAAACATGGCAAAGGTAGCGTTGCCAATGATGTTACAGATGAGATGGTCAGACTAAACTGGCTGACGGAATTTATGCCTCTTCCGACCATCAAGCATTTTATCCGTACTCCTGATGATGCATGGTTACTCACCACTGCGATCCCCGGGAAAACAGCATTCCAGGTATTAGAAGAATATCCTGATTCAGGTGAAAATATTGTTGATGCGCTGGCAGTGTTCCTGCGCCGGTTGCATTCGATTCCTGTTTGTAATTGTCCTTTTAACAGCGATCGCGTATTTCGTCTCGCTCAGGCGCAATCACGAATGAATAACGGTTTGGTTGATGCGAGTGATTTTGATGACGAGCGTAATGGCTGGCCTGTTGAACAAGTCTGGAAAGAAATGCATAAGCTTTTGCCATTCTCACCGGATTCAGTCGTCACTCATGGTGATTTCTCACTTGATAACCTTATTTTTGACGAGGGGAAATTAATAGGTTGTATTGATGTTGGACGAGTCGGAATCGCAGACCGATACCAGGATCTTGCCATCCTATGGAACTGCCTCGGTGAGTTTTCTCCTTCATTACAGAAACGGCTTTTTCAAAAATATGGTATTGATAATCCTGATATGAATAAATTGCAGTTTCATTTGATGCTCGATGAGTTTTTCTAATCAGAATTGGTTAATTGGTTGTAACACTGGCAGAGCATTACGCTGACTTGACGGGACGGCGGCTTTGTTGAATAAATCGAACTTTTGCTGAGTTGAAGGATCAGATCACGCATCTTCCCGACAACGCAGACCGTTCCGTGGCAAAGCAAAAGTTCAAAATCACCAACTGGTCCACCTACAACAAAGCTCTCATCAACCGTGGCTCCCTCACTTTCTGGCTGGATGATGGGGCGATTCAGGCCTGGTATGAGTCAGCAACACCTTCTTCACGAGGCAGACCTCAGCGCTATTCTGACCTTGCCATCACGACTGTGCTGGTCATTAAACGCGTATTCAGGCTGACCCTGCGCGCTGCGCAGGGCTTTATTGATTCCATTTTTACACTGATGAATGTTCCGTTGCGCTGCCCGGATTACAGCCGGATCCTCTAGAGTCGACCTGCAGGCATGCTGATCGGCACGTAAGAGGTTCCAACTTTCACCATAATGAAATAAGATCACTACCGGGCGTATTTTTTGAGTTATCGAGATTTTCAGGAGCTAAGGAAGCTAAAATGCGCTCACGCAACTGGTCCAGAACCTTGACCGAACGCAGCGGTGGTAACGGCGCAGTGGCGGTTTTCATGGCTTGTTATGACTGTTTTTTTGGGGTACAGTCTATGCCTCGGGCATCCAAGCAGCAAGCGCGTTACGCCGTGGGTCGATGTTTGATGTTATGGAGCAGCAACGATGTTACGCAGCAGGGCAGTCGCCCTAAAACAAAGTTAAACATCATGAGGGAAGCGGTGATCGCCGAAGTATCGACTCAACTATCAGAGGTAGTTGGCGTCATCGAGCGCCATCTCGAACCGACGTTGCTGGCCGTACATTTGTACGGCTCCGCAGTGGATGGCGGCCTGAAGCCACACAGTGATATTGATTTGCTGGTTACGGTGACCGTAAGGCTTGATGAAACAACGCGGCGAGCTTTGATCAACGACCTTTTGGAAACTTCGGCTTCCCCTGGAGAGAGCGAGATTCTCCGCGCTGTAGAAGTCACCATTGTTGTGCACGACGACATCATTCCGTGGCGTTATCCAGCTAAGCGCGAACTGCAATTTGGAGAATGGCAGCGCAATGACATTCTTGCAGGTATCTTCGAGCCAGCCACGATCGACATTGATCTGGCTATCTTGCTGACAAAAGCAAGAGAACATAGCGTTGCCTTGGTAGGTCCAGCGGCGGAGGAACTCTTTGATCCGGTTCCTGAACAGGATCTATTTGAGGCGCTAAATGAAACCTTAACGCTATGGAACTCGCCGCCCGACTGGGCTGGCGATGAGCGAAATGTAGTGCTTACGTTGTCCCGCATTTGGTACAGCGCAGTAACCGGCAAAATCGCGCCGAAGGATGTCGCTGCCGACTGGGCAATGGAGCGCCTGCCGGCCCAGTATCAGCCCGTCATACTTGAAGCTAGACAGGCTTATCTTGGACAAGAAGAAGATCGCTTGGCCTCGCGCGCAGATCAGTTGGAAGAATTTGTCCACTACGTGAAAGGCGAGATCACCAAGGTAGTCGGCAAATAAACTAGTAAATAATAAAAAAGCCGGATTAATAATCTGGCTTTTTATATTCTCTGCATAACCCTGCTTCGGGGTCATTATAGCGATTTTTTCGGTATATCCATCCTTTTTCGCACGATATACAGGATTTTGCCAAAGGGTTCGTGTAGACTTTCCTTGGTGTATCCAACGGCGTCAGCCGGGCAGGATAGGTGAAGTAGGCCCACCCGCGAGCGGGTGTTCCTTCTTCACTGTCCCTTATTCGCACCTGGCGGTGCTCAACGGGAATCCTGCTCTGCGAGGCTGGCCGTAGGCCGGCCGATAATCTCATGACCAAAATCCCTTAACGTGAGTTTTCGTTCCACTGAGCGTCAGACCCCGTAGAAAAGATCAAAGGATCTTCTTGAGATCCTTTTTTTCTGCGCGTAATCTGCTGCTTGCAAACAAAAAAACCACCGCTACCAGCGGTGGTTTGTTTGCCGGATCAAGAGCTACCAACTCTTTTTCCGAAGGTAACTGGCTTCAGCAGAGCGCAGATACCAAATACTGTTCTTC

>pHal**V**-FAP_G462V_RFP GenBank:MW076830

TAGTGTAGCCGTAGTTAGGCCACCACTTCAAGAACTCTGTAGCACCGCCTACATACCTCGCTCTGCTAATCCTGTTACCAGTGGCTGCTGCCAGTGGCGATAAGTCGTGTCTTACCGGGTTGGACTCAAGACGATAGTTACCGGATAAGGCGCAGCGGTCGGGCTGAACGGGGGGTTCGTGCACACAGCCCAGCTTGGAGCGAACGACCTACACCGAACTGAGATACCTACAGCGTGAGCTATGAGAAAGCGCCACGCTTCCCGAAGGGAGAAAGGCGGACAGGCATCCGGTAAGCGGCAGGGTCGGAACAGGAGAGCGCACGAGGGAGCTTCCAGGGGGAAACGCCTGGTATCTTTATAGTCCTGTCGGGTTTCGCCACCTCTGACTTGAGCGTCGATTTTTGTGATGCTCGTCAGGGGGGCGGAGCCTATGGAAAAACGCCAGCAACGCGGCCGTGAAAGGCAGGCCGGTCCGTGGTGGCCACGGCCTCTAGGCCAGATCCAGCGGCATCTGGGTTAGTCGAGCGCGGGCCGCTTCCCATGTCTCACCAGGGCGAGCCTGTTTCGCGATCTCAGCATCTGAAATCTTCCCGGCCTTGCGCTTCGCTGGGGCCTTACCCACCGCCTTGGCGGGCTTCTTCGGTCCAAAACTGAACAACAGATGTGTGACCTTGCGCCCGGTCTTTCGCTGCGCCCACTCCACCTGTAGCGGGCTGTGCTCGTTGATCTGCGTCACGGCTGGATCAAGCACTCGCAACTTGAAGTCCTTGATCGAGGGATACCGGCCTTCCAGTTGAAACCACTTTCGCAGCTGGTCAATTTCTATTTCGCGCTGGCCGATGCTGTCCCATTGCATGAGCAGCTCGTAAAGCCTGATCGCGTGGGTGCTGTCCATCTTGGCCACGTCAGCCAAGGCGTATTTGGTGAACTGTTTGGTGAGTTCCGTCAGGTACGGCAGCATGTCTTTGGTGAACCTGAGTTCTACACGGCCCTCACCCTCCCGGTAGATGATTGTTTGCACCCAGCCGGTAATCATCACACTCGGTCTTTTCCCCTTGCCATTGGGCTCTTGGGTTAACCGGACTTCCCGCCGTTTCAGGCGCAGGGCCGCTTCTTTGAGCTGGTTGTAGGAAGATTCGATAGGGACACCCGCCATCGTCGCTATGTCCTCCGCCGTCACTGAATACATCACTTCATCGGTGACAGGCTCGCTCCTCTTCACCTGGCTAATACAGGCCAGAACGATCCGCTGTTCCTGAACACTGAGGCGATACGCGGCCTCGACCAGGGCATTGCTTTTGTAAACCATTGGGGGTGAGGCCACGTTCGACATTCCTTGTGTATAAGGGGACACTGTATCTGCGTCCCACAATACAACAAATCCGTCCCTTTACAACAACAAATCCGTCCCTTCTTAACAACAAATCCGTCCCTTAATGGCAACAAATCCGTCCCTTTTTAAACTCTAGAGGCCACGGATTACGTGGCCTGTAGACGTCCTAAAAGGTTTAAAAGGGAAAAGGAAGAAAAGGGTGGAAACGCAAAAAACGCACCACTACGTGGCCCCGTTGGGGCCGCATTTGTGCCCCTGAAGGGGCGGGGGAGGCGTCTGGGCAATCCCCGTTTTACCAGTCCCCTATCGCCGCCTGAGAGGGCGCAGGAAGCGAGTAATCAGGGTATCGAGGCGGATTCACCCTTGGCGTCCAACCAGCGGCACCAGCGGCGCCTGAGAGGGGCGCGCCCAGCTGTCTAGGGCGGCGGATTTGTCCTACTCAGGAGAGCGTTCACCGACAAACAACAGATAAAACGAAAGGCCCAGTCTTTCGACTGAGCCTTTCGTTTTATTTGATGCCTTTAATTAAAGCGGATAACAATTTCACACAGGAGGTTTTTCTATTGCGT***NNNNNNNNNNNNN***GTATAGAGTTTGAGACTTTACTAGAGAAAGAGGAGAAATACTAG**ATG**GCCAGCGCAGTTGAAGATATTCGTAAAGTTCTGAGCGATAGCAGCAGTCCGGTTGCAGGTCAGAAATATGATTATATTCTGGTTGGTGGTGGCACCGCAGCATGTGTTCTGGCAAATCGTCTGAGCGCAGATGGTAGCAAACGTGTTCTGGTTCTGGAAGCAGGTCCGGATAATACCAGCCGTGATGTTAAAATTCCGGCAGCAATTACCCGTCTGTTTCGTAGTCCGCTGGATTGGAACCTGTTTAGCGAACTGCAAGAACAGCTGGCAGAACGTCAGATTTATATGGCACGTGGTCGTCTGTTAGGTGGTAGCAGCGCAACCAATGCAACCCTGTATCATCGTGGTGCAGCCGGTGATTATGATGCATGGGGTGTTGAAGGTTGGAGCAGCGAAGATGTTCTGAGCTGGTTTGTTCAGGCAGAAACCAATGCCGATTTTGGTCCGGGTGCATATCATGGTAGCGGTGGTCCGATGCGTGTTGAAAATCCGCGTTATACCAATAAACAGCTGCATACCGCATTTTTCAAAGCAGCAGAAGAAGTTGGTCTGACCCCGAATAGCGATTTTAATGATTGGAGCCATGATCACGCAGGTTATGGCACCTTTCAGGTTATGCAGGATAAAGGCACCCGTGCAGATATGTATCGTCAGTATCTGAAACCGGTTCTGGGTCGTCGTAATCTGCAGGTTCTGACCGGTGCAGCAGTTACCAAAGTTAATATTGATCAGGCAGCAGGTAAAGCACAGGCACTGGGCGTTGAATTTTCAACCGATGGTCCGACCGGTGAACGTCTGTCAGCAGAACTGGCACCTGGTGGTGAAGTTATTATGTGTGCCGGTGCAGTTCATACCCCGTTTCTGCTGAAACATAGCGGTGTTGGTCCGAGCGCAGAACTGAAAGAATTTGGTATTCCGGTTGTTAGCAATCTGGCAGGCGTTGGCCAGAATCTGCAAGATCAGCCTGCATGTCTGACCGCAGCACCGGTTAAAGAAAAATATGATGGTATTGCCATCAGCGACCACATCTATAATGAAAAAGGTCAGATTCGCAAACGTGCCATTGCAAGCTATCTGCTTGGTGGTCGTGGTGGCCTGACCAGTACCGGTTGTGATCGCGGTGCATTTGTTCGTACCGCAGGTCAGGCACTGCCGGATCTGCAAGTTCGTTTTGTTCCTGGTATGGCACTGGATCCGGATGTTGTTAGCACCTATGTGCGTTTTGCCAAATTTCAGAGCCAGGGCCTGAAATGGCCGAGCGGTATTACCATGCAGCTGATTGCATGTCGTCCGCAGAGTACCGGTAGCGTGGGTCTGAAAAGCGCAGATCCGTTTGCACCGCCTAAACTGAGTCCGGGTTATCTGACCGATAAAGATGGTGCAGATCTGGCGACCCTGCGTAAAGGTATTCATTGGGCACGTGATGTTGCACGTAGCTCAGCACTGAGCGAATATCTGGATGGTGAACTGTTTCCTGGTAGTGGTGTTGTTAGTGATGATCAGATCGATGAATATATCCGTCGTAGCATTCATAGCAGCAATGCAATTACCGGCACCTGTAAAATGGGTAATGCCGGTGATAGCAGCTCAGTTGTTGATAACCAGCTGCGTGTGCATGGTGTGGAAGGTCTGCGTGTTGTTGATGCAAGCGTTGTTCCGAAAATTCCTGGTGGTCAGACAGGTGCTCCGGTGGTGATGATTGCAGAACGTGCAGCAGCCCTGCTGACCGGTAAAGCAACCATTGGTGCAAGCGCAGCCGCACCGGCAACCGTTGCAGCAGGTTCTGCGGGTTCTGCGGCCGGTTCTGGCGAATTTATGGCGAGTAGCGAAGACGTTATCAAAGAGTTCATGCGTTTCAAAGTTCGTATGGAAGGTTCCGTTAACGGTCACGAGTTCGAAATCGAAGGTGAAGGTGAAGGTCGTCCGTACGAAGGTACCCAGACCGCTAAACTGAAAGTTACCAAAGGTGGTCCGCTGCCGTTCGCTTGGGACATCCTGTCCCCGCAGTTCCAGTACGGTTCCAAAGCTTACGTTAAACACCCGGCTGACATCCCGGACTACCTGAAACTGTCCTTCCCGGAAGGTTTCAAATGGGAACGTGTTATGAACTTCGAAGACGGTGGTGTTGTTACCGTTACCCAGGACTCCTCCCTGCAAGACGGTGAGTTCATCTACAAAGTTAAACTGCGTGGTACCAACTTCCCGTCCGACGGTCCGGTTATGCAGAAAAAAACCATGGGTTGGGAAGCTTCCACCGAACGTATGTACCCGGAAGACGGTGCTCTGAAAGGTGAAATCAAAATGCGTCTGAAACTGAAAGACGGTGGTCACTACGACGCTGAAGTTAAAACCACCTACATGGCTAAAAAACCGGTTCAGCTGCCGGGTGCTTACAAAACCGACATCAAACTGGACATCACCTCCCACAACGAAGACTACACCATCGTTGAACAGTACGAACGTGCTGAAGGTCGTCACTCCACCGGTGCT**TAA**TTAACCTAGGCTGCTGCCACCGCTGAGCAATAAAGCCCATGCAAGCTTGCGGCCGCGTCGTGACTGGGAAAACCCTGGCGACTAGTCTTGGACTCCTGTTGATAGATCCAGTAATGACCTCAGAACTCCATCTGGATTTGTTCAGAACGCTCGGTTGCCGCCGGGCGTTTTTTATTGGTGAGAATCCAGACGTTGTGTCTCAAAATCTCTGATGTTACATTGCACAAGATAAAAATATATCATCATGAACAATAAAACTGTCTGCTTACATAAACAGTAATACAAGGGGTGTTATGAGCCATATTCAACGGGAAACGTCTTGCTCGAGGCCGCGATTAAATTCCAACATGGATGCTGATTTATATGGGTATAAATGGGCTCGCGATAATGTCGGGCAATCAGGTGCGACAATCTATCGATTGTATGGGAAGCCCGATGCGCCAGAGTTGTTTCTGAAACATGGCAAAGGTAGCGTTGCCAATGATGTTACAGATGAGATGGTCAGACTAAACTGGCTGACGGAATTTATGCCTCTTCCGACCATCAAGCATTTTATCCGTACTCCTGATGATGCATGGTTACTCACCACTGCGATCCCCGGGAAAACAGCATTCCAGGTATTAGAAGAATATCCTGATTCAGGTGAAAATATTGTTGATGCGCTGGCAGTGTTCCTGCGCCGGTTGCATTCGATTCCTGTTTGTAATTGTCCTTTTAACAGCGATCGCGTATTTCGTCTCGCTCAGGCGCAATCACGAATGAATAACGGTTTGGTTGATGCGAGTGATTTTGATGACGAGCGTAATGGCTGGCCTGTTGAACAAGTCTGGAAAGAAATGCATAAGCTTTTGCCATTCTCACCGGATTCAGTCGTCACTCATGGTGATTTCTCACTTGATAACCTTATTTTTGACGAGGGGAAATTAATAGGTTGTATTGATGTTGGACGAGTCGGAATCGCAGACCGATACCAGGATCTTGCCATCCTATGGAACTGCCTCGGTGAGTTTTCTCCTTCATTACAGAAACGGCTTTTTCAAAAATATGGTATTGATAATCCTGATATGAATAAATTGCAGTTTCATTTGATGCTCGATGAGTTTTTCTAATCAGAATTGGTTAATTGGTTGTAACACTGGCAGAGCATTACGCTGACTTGACGGGACGGCGGCTTTGTTGAATAAATCGAACTTTTGCTGAGTTGAAGGATCAGATCACGCATCTTCCCGACAACGCAGACCGTTCCGTGGCAAAGCAAAAGTTCAAAATCACCAACTGGTCCACCTACAACAAAGCTCTCATCAACCGTGGCTCCCTCACTTTCTGGCTGGATGATGGGGCGATTCAGGCCTGGTATGAGTCAGCAACACCTTCTTCACGAGGCAGACCTCAGCGCTATTCTGACCTTGCCATCACGACTGTGCTGGTCATTAAACGCGTATTCAGGCTGACCCTGCGCGCTGCGCAGGGCTTTATTGATTCCATTTTTACACTGATGAATGTTCCGTTGCGCTGCCCGGATTACAGCCGGATCCTCTAGAGTCGACCTGCAGGCATGCTGATCGGCACGTAAGAGGTTCCAACTTTCACCATAATGAAATAAGATCACTACCGGGCGTATTTTTTGAGTTATCGAGATTTTCAGGAGCTAAGGAAGCTAAAATGCGCTCACGCAACTGGTCCAGAACCTTGACCGAACGCAGCGGTGGTAACGGCGCAGTGGCGGTTTTCATGGCTTGTTATGACTGTTTTTTTGGGGTACAGTCTATGCCTCGGGCATCCAAGCAGCAAGCGCGTTACGCCGTGGGTCGATGTTTGATGTTATGGAGCAGCAACGATGTTACGCAGCAGGGCAGTCGCCCTAAAACAAAGTTAAACATCATGAGGGAAGCGGTGATCGCCGAAGTATCGACTCAACTATCAGAGGTAGTTGGCGTCATCGAGCGCCATCTCGAACCGACGTTGCTGGCCGTACATTTGTACGGCTCCGCAGTGGATGGCGGCCTGAAGCCACACAGTGATATTGATTTGCTGGTTACGGTGACCGTAAGGCTTGATGAAACAACGCGGCGAGCTTTGATCAACGACCTTTTGGAAACTTCGGCTTCCCCTGGAGAGAGCGAGATTCTCCGCGCTGTAGAAGTCACCATTGTTGTGCACGACGACATCATTCCGTGGCGTTATCCAGCTAAGCGCGAACTGCAATTTGGAGAATGGCAGCGCAATGACATTCTTGCAGGTATCTTCGAGCCAGCCACGATCGACATTGATCTGGCTATCTTGCTGACAAAAGCAAGAGAACATAGCGTTGCCTTGGTAGGTCCAGCGGCGGAGGAACTCTTTGATCCGGTTCCTGAACAGGATCTATTTGAGGCGCTAAATGAAACCTTAACGCTATGGAACTCGCCGCCCGACTGGGCTGGCGATGAGCGAAATGTAGTGCTTACGTTGTCCCGCATTTGGTACAGCGCAGTAACCGGCAAAATCGCGCCGAAGGATGTCGCTGCCGACTGGGCAATGGAGCGCCTGCCGGCCCAGTATCAGCCCGTCATACTTGAAGCTAGACAGGCTTATCTTGGACAAGAAGAAGATCGCTTGGCCTCGCGCGCAGATCAGTTGGAAGAATTTGTCCACTACGTGAAAGGCGAGATCACCAAGGTAGTCGGCAAATAAACTAGTAAATAATAAAAAAGCCGGATTAATAATCTGGCTTTTTATATTCTCTGCATAACCCTGCTTCGGGGTCATTATAGCGATTTTTTCGGTATATCCATCCTTTTTCGCACGATATACAGGATTTTGCCAAAGGGTTCGTGTAGACTTTCCTTGGTGTATCCAACGGCGTCAGCCGGGCAGGATAGGTGAAGTAGGCCCACCCGCGAGCGGGTGTTCCTTCTTCACTGTCCCTTATTCGCACCTGGCGGTGCTCAACGGGAATCCTGCTCTGCGAGGCTGGCCGTAGGCCGGCCGATAATCTCATGACCAAAATCCCTTAACGTGAGTTTTCGTTCCACTGAGCGTCAGACCCCGTAGAAAAGATCAAAGGATCTTCTTGAGATCCTTTTTTTCTGCGCGTAATCTGCTGCTTGCAAACAAAAAAACCACCGCTACCAGCGGTGGTTTGTTTGCCGGATCAAGAGCTACCAACTCTTTTTCCGAAGGTAACTGGCTTCAGCAGAGCGCAGATACCAAATACTGTTCTTC

**References**

1. Amer, M. Wojcik, E.Z. Sun, C. Hoeven, R. Hughes, J.M.X. Faulkner, M. Yunus, I.S. Tait, S. Johannissen, L.O. Hardman, S.J.O. Heyes, D.J. Chen, G.-Q. Smith, M.H. Jones, P.R. Toogood, H.S. and Scrutton, N.S. (2020) Low carbon strategies for sustainable bio-alkane gas production and renewable energy. *Energy. Environ. Sci.,* 29, https://doi.org/10.1039/D1030EE00095G.

2. Lee, T.S. Krupa, R.A. Zhang, F. Hajimorad, M. Holtz, W.J. Prasad, N. Lee, S.K. and Keasling, J.D. (2011) BglBrick vectors and datasheets: A synthetic biology platform for gene expression. *J. Biol. Eng.,* 5, 15-17.

3. Li, T. Li, T. Ji, W. Wang, Q. Zhang, H. Chen, G.-Q. Lou, C. and Ouyang, Q. (2016) Engineering of core promoter regions enables the construction of constitutive and inducible promoters in *Halomonas* sp. *Biotechnol. J.,* 11, 219-227.
